# The global death and disability burden associated with a high BMI in children and adolescents, 1990–2019

**DOI:** 10.3389/fendo.2024.1463002

**Published:** 2024-10-08

**Authors:** Ying Song, Yuan Zhou, Xiaojin Feng, Jieting Fu, Yongping Liu

**Affiliations:** ^1^ Laboratory for Stem Cell and Regenerative Medicine, Affiliated Hospital of Shandong Second Medical University, Weifang, China; ^2^ Department of Hematology, Affiliated Hospital of Shandong Second Medical University, Weifang, China; ^3^ Department of Endocrinology and Metabolism, Affiliated Hospital of Shandong Second Medical University, Weifang, China; ^4^ Department of Clinical Research Center, Affiliated Hospital of Shandong Second Medical University, Weifang, China

**Keywords:** BMI, obesity, children, adolescent, global burden

## Abstract

**Objective:**

Exploring changing trends in the burden caused by overweight and obesity among children and adolescents from 1990 to 2019 at the global, regional, and national levels, based on data from the Global Burden of Disease study (GBD) 2019.

**Methods:**

The annual number and rate of deaths and disability-adjusted life years (DALYs) associated with a high BMI among children and adolescents at global, regional, and national levels by age groups, sexes, and the sociodemographic index from 1990 to 2019 were collected from the GBD study 2019. Change percentage for number, and the estimated annual percentage changes (EAPCs) for rate were calculated to determine the temporal trends.

**Results:**

From 1990 to 2019, global high BMI-related deaths decreased by 34% but DALYs increased by 48%. Death rates in females were higher than in males, although both showed decreasing trends. For the rate of DALYs, both sexes showed increasing trends, but since 1999, the rate in males has surpassed that in females. A high BMI had the greatest impact on children under 5 years of age, and the burden in other age groups continued to increase. Regionally, High-income Asia Pacific experienced the fastest decrease in death rate (EAPC=−9.57), and East Asia saw the fastest increase in the DALYs rate (EAPC= 3.47). Globally, as age increases, the proportion of disease burden attributed to a high BMI in females generally increases.

**Conclusions:**

Our findings emphasize the urgent need to improve efforts to prevent children and adolescents becoming overweight and obese.

## 1 Introduction

Epidemiological evidence has confirmed that the early nutritional status plays a crucial role in determining health status throughout the life course (1). With the rising prevalence of obesity, it has escalated into a global public health concern. Excess weight and obesity among children and adolescents has become increasingly alarming, impinging on their healthy development (2). In 2020, approximately 2.6 billion children and adolescents worldwide were overweight, accounting for approximately 38% of the world’s population, and this is expected to reach 51% by 2035. Recent estimates have indicated that by 2050, approximately 57% of children and adolescents aged 2 to 19 years of age will be obese (3).

Despite the current severity of the situation, the risk factors associated with overweight and obesity have not received the attention they deserve, especially among children and adolescents. Being overweight or obese can lead to a range of consequences for children and adolescents, including a higher susceptibility to emotional and behavioral disorders (4) and chronic diseases such as asthma (5), high blood pressure, hyperlipidemia, diabetes, and cardiovascular disease (6). Furthermore, individuals who have been overweight or obese from childhood face greater challenges in the treatment of metabolic disorders in adulthood (7). These disease dynamics place a heavy burden on patients and their families, underscoring the necessity to prevent overweight and obesity as early in life as possible.

Overweight and obesity are estimated based on the body mass index (BMI). Therefore, understanding trends in the BMI in children and adolescents is a key strategy for the early prevention of overweight and obesity. Regular evaluation of global patterns of change in the BMI among this age group, including updated risk assessments, is vital in preventing long-term complications caused by obesity. To the best of our knowledge, no comprehensive long-term trends in disease burden due to a high BMI in children and adolescents have been reported.

In this study, we utilized the Global Burden of Disease (GBD) Study 2019 (GBD 2019) database to examine changing trends in disease burden [including deaths and disability-adjusted life years (DALYs)] associated with a high BMI in children and adolescents globally 1990 to 2019 globally. It is hoped that this interpretation of the GBD 2019 estimates will assist policymakers in constructing a more targeted health system, designed to implement specialized effective interventions aimed at reducing the burden of obesity and other diseases and health risks associated with a high BMI in children and adolescents.

## 2 Materials and methods

### 2.1 Data sources

The data utilized in this study were obtained from the GBD 2019 database in the Global Health Online Data Exchange Query Tool (http://ghdx.healthdata.org/gbdresults-tool). The GBD 2019 incorporates diverse sources of data input, including census, vital statistics, and investigations. It employs standardized methodologies to estimate disease prevalence and mortality rates across all countries and regions worldwide, with all data visualized online (8, 9). The tool provides global, regional, and national data on the burden of diseases related to a high BMI. In the GBD 2019, adults with a BMI of over 25 kg/m^2^ or children (aged 0–19 years) exceeding the corresponding standards threshold set by the International Obesity Task Force, are classified as having a high BMI (10). We collected existing data associated with a high BMI on children and adolescents under 20 years old. To summarize the age distribution of a high BMI burden among children and adolescents, individuals were categorized into four groups: 0–4 years old, 5–9 years old, 10–14 years old, and 15–19 years old.

### 2.2 Definitions

#### 2.2.1 DALYs

Disability-adjusted life years (DALYs) provides a comprehensive assessment of disease burden, representing the cumulative loss of healthy life years due to premature death and disability (11). It is calculated by summing up the years lived with disability (YLDs) and the years of life lost (YLLs). The methodologies for calculating YLDs, YLLs, and DALYs have been described previously (12). In brief, YLLs are determined by multiplying the number of deaths at each age by the standard life expectancy, whereas YLDs are derived from multiplying the prevalence of each sequela by its corresponding disability weight. One DALY can be considered equivalent to one year of fully healthy life (13).

#### 2.2.2 SDI

The sociodemographic index (SDI), ranging from 0 to 1, serves as a composite index to assess the social development status calculated based on information at state level, including the average education level among individuals over 15 years old, total fertility rate among individuals below 25 years old, and lag-distributed income per capita income. Higher values indicate greater levels of social development. This study categorized 204 countries and territories worldwide into five SDI regions (low, low-middle, middle, high-middle, and high) to investigate the association between a high BMI burden and socioeconomic development among children and adolescents.

### 2.3 Statistical analysis

The number and rate of deaths and DALYs are key indicators used to depict the burden of disease associated with the BMI in children and adolescents worldwide, either overall or by year, region, age, and sex. The estimation for the burden of disease is described with a statistically significant 95% uncertain interval (95% UI), representing the true value of the parameter with a 95% probability, taking several uncertainties in parameter estimation into account, such as data collection, model selection, and other uncertainties. We calculated the mean estimated annual percentage change (EAPC) and obtained a 95% confidence interval (95% CI) for EAPC to analyze time trends in disease burden due to a high BMI in children and adolescents (14, 15). When both the EAPC and its lower limit at a 95% CI were greater than 0, the time trend was considered to be increasing. Conversely, when both the EAPC and its upper limit at a 95% CI were less than 0, the time trend was considered to be declining. In addition, population attribution scores (PAF) were also calculated to measure the proportion of deaths associated with a high BMI (10).

In this study, we systematically and meticulously analyzed trends in deaths and DALYs associated with a high BMI among children and adolescents at the global, geographic, national, and SDI region levels by sex and age group. Finally, we analyzed the influential factors of the EAPC. All analysis and calculations were conducted using R software (version 4.2.2). *P*<0.05 was considered statistically significant.

## 3 Results

### 3.1 Global burden due to a high BMI in children and adolescents

#### 3.1.1 Overall trend

**Figure 1:**
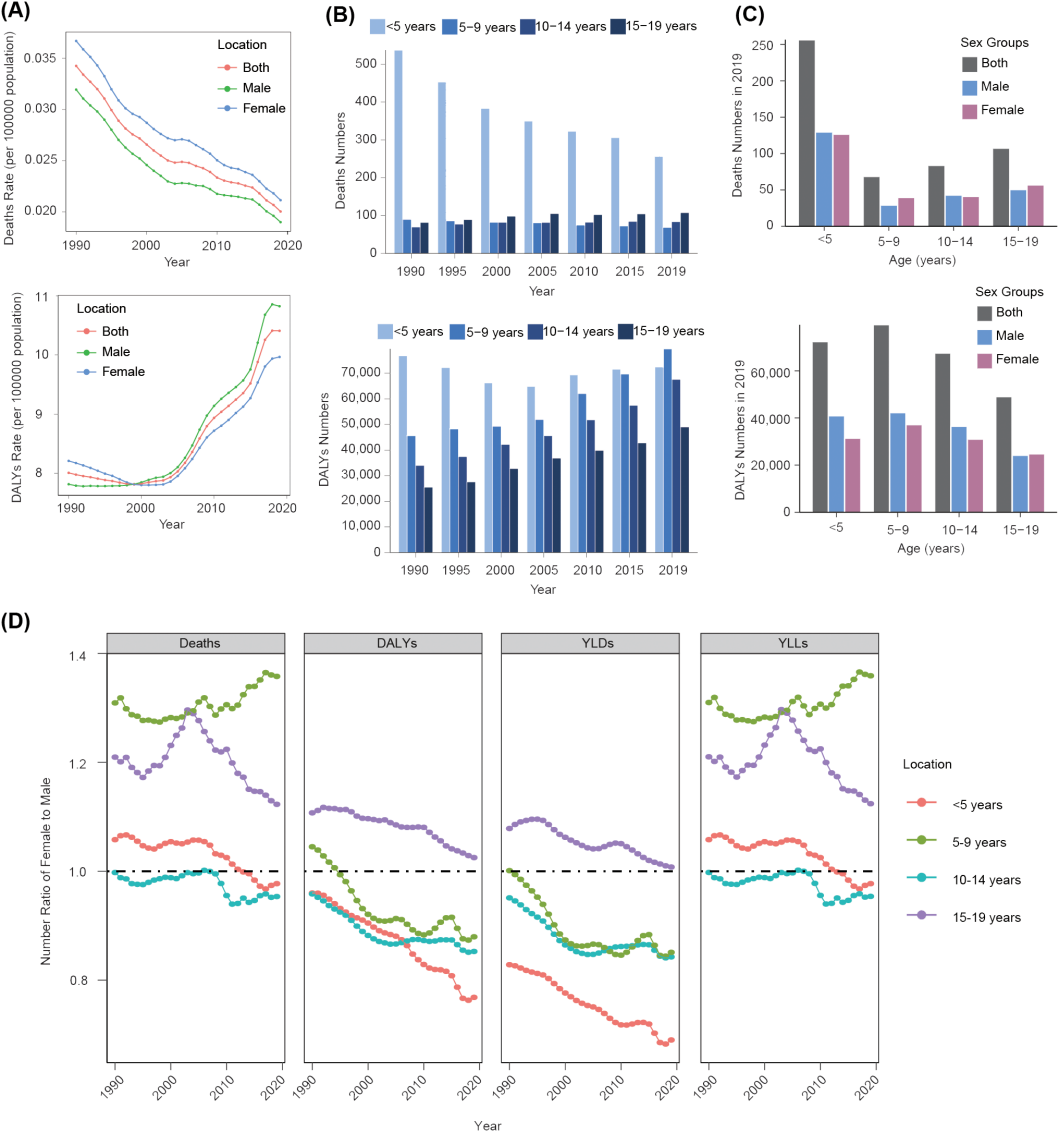
Global trends of disease burden attributable to high BMI among children and adolescents aged under 20 years from 1990 to 2019 by sex and age group. **(A)** Deaths and DALYs rate changing trends by sex. **(B)** Deaths and DALYs numbers in different age groups. **(C)** Deaths and DALYs numbers in different age groups by sex in 2019. **(D)** Number ratio of female to male trends from 1990 to 2019 by age group.

In 2019, there were 517 (95% UI: 279 to 812) deaths linked to a high BMI, with a reduction of 34% from 779 (95% UI: 369 to 1257) in 1990 (**Table 1**; **Supplementary Figure S1**). The corresponding rates have decreased from 0.03 to 0.02 per 100,000 people, with an associated EAPC of −1.62 (95% CI: −1.74 to −1.5) (**Figure 1A**; **Supplementary Table S1**). Interestingly, despite the decline of deaths, the global DALYs showed an increasing trend over this period, rising from 182,007 (95% UI: 91,485 to 312,535) to 268,490 (95% UI: 125,573 to 481,558) (**Table 1**). The YLDs rate showed the same trend (**Supplementary Figure S2**, **Supplementary Table S2**), whereas the YLLs rate displayed a downward trend from 1990 to 2019 (**Supplementary Figure S3**, **Supplementary Table S3**). Over this period, the DALYs rate increased from 8.01 (95% UI: 4.02 to 13.75) to 10.41 (95% UI: 4.87 to 18.67) per 100,000 people, with an associated EAPC of 0.98 (95% CI: 0.79 to 1.17) (**Figure 1A**, **Supplementary Table S2**).

**Table 1:** Deaths and DALYs linked to a high BMI in children and adolescents at global, SDI, and geographical regional levels from 1990 to 2019.

	Deaths			DALYs		
Location	Death numbers in 1990 (95%UI)	Death numbers in 2019 (95%UI)	Death numbers change, 1990-2019 (%)	DALYs numbers in 1990 (95%UI)	DALYs numbers in 2019 (95%UI)	DALYs numbers change, 1990-2019 (%)
**Global**	779 (369 to 1257)	517 (279 to 812)	-0.34 (-0.5 to 0.01)	182007 (91485 to 312535)	268490 (125573 to 481558)	0.48 (0.24 to 0.74)
SDI						
High SDI	42 (24 to 61)	28 (16 to 41)	-0.34 (-0.41 to -0.24)	40364 (18264 to 76049)	63117 (28952 to 117269)	0.56 (0.42 to 0.75)
High-middle SDI	53 (28 to 84)	20 (11 to 31)	-0.62 (-0.72 to -0.47)	24902 (11561 to 45573)	34803 (15281 to 66078)	0.4 (0.23 to 0.57)
Middle SDI	270 (132 to 431)	126 (69 to 193)	-0.53 (-0.65 to -0.31)	56205 (28113 to 95988)	76755 (35168 to 140785)	0.37 (0.09 to 0.69)
Low-middle SDI	201 (89 to 339)	124 (67 to 196)	-0.38 (-0.55 to -0.02)	31524 (15376 to 52767)	43025 (20464 to 75779)	0.36 (0.03 to 0.81)
Low SDI	212 (84 to 379)	217 (107 to 376)	0.03 (-0.31 to 0.93)	28786 (13316 to 49113)	50511 (25083 to 87351)	0.75 (0.32 to 1.49)
Regions						
Central Asia	4 (2 to 6)	2 (1 to 2)	-0.59 (-0.7 to -0.33)	1749 (805 to 3224)	2698 (1134 to 5203)	0.54 (0.3 to 0.78)
Central Europe	2 (1 to 3)	0 (0 to 1)	-0.84 (-0.88 to -0.79)	2547 (1109 to 4880)	2529 (1064 to 4936)	-0.01 (-0.11 to 0.1)
Eastern Europe	5 (2 to 7)	1 (1 to 1)	-0.8 (-0.84 to -0.71)	4901 (2097 to 9400)	4274 (1789 to 8563)	-0.13 (-0.23 to -0.01)
Australasia	2 (1 to 3)	1 (1 to 2)	-0.5 (-0.62 to -0.34)	2090 (963 to 3752)	2341 (1073 to 4563)	0.12 (-0.12 to 0.45)
High-income Asia Pacific	7 (4 to 10)	0 (0 to 1)	-0.94 (-0.96 to -0.91)	4082 (1844 to 7682)	2961 (1241 to 5747)	-0.27 (-0.39 to -0.16)
High-income North America	17 (10 to 25)	20 (12 to 29)	0.19 (0.03 to 0.37)	22814 (9942 to 43871)	42566 (19452 to 76830)	0.87 (0.57 to 1.28)
Southern Latin America	1 (1 to 2)	1 (0 to 1)	-0.36 (-0.55 to -0.13)	1344 (602 to 2527)	2929 (1290 to 5806)	1.18 (0.77 to 1.73)
Western Europe	13 (7 to 19)	4 (2 to 7)	-0.67 (-0.71 to -0.59)	11680 (5352 to 21787)	14583 (6673 to 27913)	0.25 (0.1 to 0.39)
Andean Latin America	48 (19 to 89)	4 (2 to 8)	-0.91 (-0.95 to -0.78)	9405 (4231 to 16480)	7055 (3069 to 13968)	-0.25 (-0.49 to 0.07)
Caribbean	20 (8 to 37)	12 (5 to 23)	-0.38 (-0.59 to -0.05)	4357 (2059 to 7689)	4322 (2005 to 7851)	-0.01 (-0.2 to 0.18)
Central Latin America	57 (26 to 88)	13 (7 to 22)	-0.77 (-0.84 to -0.61)	15192 (7316 to 26545)	15565 (6946 to 29785)	0.02 (-0.19 to 0.27)
Tropical Latin America	26 (11 to 46)	11 (6 to 18)	-0.59 (-0.73 to -0.29)	12230 (5534 to 22160)	19427 (8534 to 36745)	0.59 (0.25 to 1.02)
North Africa and Middle East	140 (66 to 235)	55 (29 to 94)	-0.6 (-0.74 to -0.31)	22782 (11662 to 37900)	30133 (13884 to 54581)	0.32 (-0.04 to 0.75)
South Asia	85 (35 to 152)	49 (26 to 79)	-0.42 (-0.61 to 0.01)	12100 (5557 to 20960)	15494 (7048 to 28389)	0.28 (-0.12 to 0.84)
East Asia	19 (8 to 31)	3 (2 to 5)	-0.83 (-0.89 to -0.66)	8182 (3637 to 15406)	15520 (6288 to 30891)	0.9 (0.58 to 1.16)
Oceania	3 (1 to 6)	5 (2 to 9)	0.57 (0.02 to 1.33)	425 (209 to 715)	776 (383 to 1389)	0.83 (0.36 to 1.36)
Southeast Asia	95 (42 to 161)	89 (47 to 136)	-0.07 (-0.34 to 0.54)	12396 (5966 to 20903)	20253 (10070 to 35365)	0.63 (0.24 to 1.27)
Central Sub-Saharan Africa	35 (10 to 75)	32 (14 to 62)	-0.08 (-0.48 to 1.15)	4254 (1589 to 8195)	6975 (3356 to 12515)	0.64 (0.02 to 1.88)
Eastern Sub-Saharan Africa	141 (55 to 256)	123 (57 to 226)	-0.13 (-0.46 to 0.88)	19709 (9080 to 33187)	30898 (15114 to 53835)	0.57 (0.15 to 1.3)
Southern Sub-Saharan Africa	22 (9 to 38)	10 (5 to 16)	-0.54 (-0.69 to -0.28)	3714 (1722 to 6327)	4446 (1990 to 8216)	0.2 (-0.13 to 0.59)
Western Sub-Saharan Africa	37 (17 to 65)	81 (38 to 147)	1.15 (0.52 to 2.13)	6055 (2843 to 10361)	22744 (10618 to 40688)	2.76 (1.98 to 3.71)

BMI, body mass index; DALYs, disability-adjusted life-years; 95% UI, 95% uncertainty interval.

#### 3.1.2 Gender difference and the corresponding changing trend

In terms of gender disparities, the global rate of deaths attributed to a high BMI among children and adolescents was consistently higher in females than in males over the past three decades, albeit with both showing a declining trend (a decrease of 42% in females and 41% in males) (**Figure 1A**). Regarding the DALYs rate, both males and females exhibited an upward trend, with males surpassing females since 1999 and subsequently widening the gap over time (**Figure 1A**). Correspondingly, the YLDs rate demonstrated an increasing trend for both sexes, whereas the YLLs rate showed a decreasing trend from 1990 to 2019 (**Supplementary Figure S3**). These findings indicated that there was an unequal burden of disease caused by a high BMI between sexes among children and adolescents. Without prompt intervention, the disparity will continue to widen.

#### 3.1.3 Age difference and the corresponding changing trend

To illustrate the trend in disease burden in different age groups, we categorized individuals under 20 years of age into four distinct cohorts: 0–4, 5–9, 10–14, and 15–19 years of age. We found that the number of deaths in children aged 0–4 years was greatest from 1990 to 2019, even though this group has shown the most substantial reductions in both the number of deaths and death rate, with declines of approximately 52% and 54%, respectively (**Figure 1B**; **Supplementary Figure S2**). Although, in total, more females died than males in 2019 (**Supplementary Figure S1**), males exhibited a higher number of deaths in the 0–4-year-old and 10–14-year-old age groups (**Figure 1C**). Unlike the decreasing trend in the number of deaths, from 1990 to 2019, the DALYs numbers showed an increasing trend in all the age groups, except in the 0–4-year-old group (**Figure 1B**). In 2019, males demonstrated a higher DALYs burden across three other age groups except for a slight elevation in the female burden compared with males in the 15–19 year-old group (**Figure 1C**). In children aged under 15 years, the YLDs burden was mainly concentrated in males, but the YLLs burden was balanced among the two sexes over all the age groups (**Supplementary Figure S4**).

We further calculated the number ratio of females-to-males (F/M) to explore the gender difference and changing trend at various ages. As shown in **Figure 1D**, the disease burden due to a high BMI has been skewed toward males year by year, except death and YLLs burden among children aged 5–9 years. For the death burden and YLLs burden, nevertheless, only children aged 10–14 years exhibited a male-dominated pattern (F/M <1). In the 0–4, 5–9, and 10–14 age groups, for DALYs and YLDs, the disease burden was mainly concentrated in males. However, in the 15–19-year-old age group, it was mainly concentrated in females, even though there was a gradual shift to males (**Figure 1D**). These findings collectively suggested an imbalanced distribution of a high BMI-related disease burden among children and adolescents across several age cohorts.

### 3.2 Geographical regional burden due to a high BMI in children and adolescents

#### 3.2.1 Overall trend

**Figure 2:**
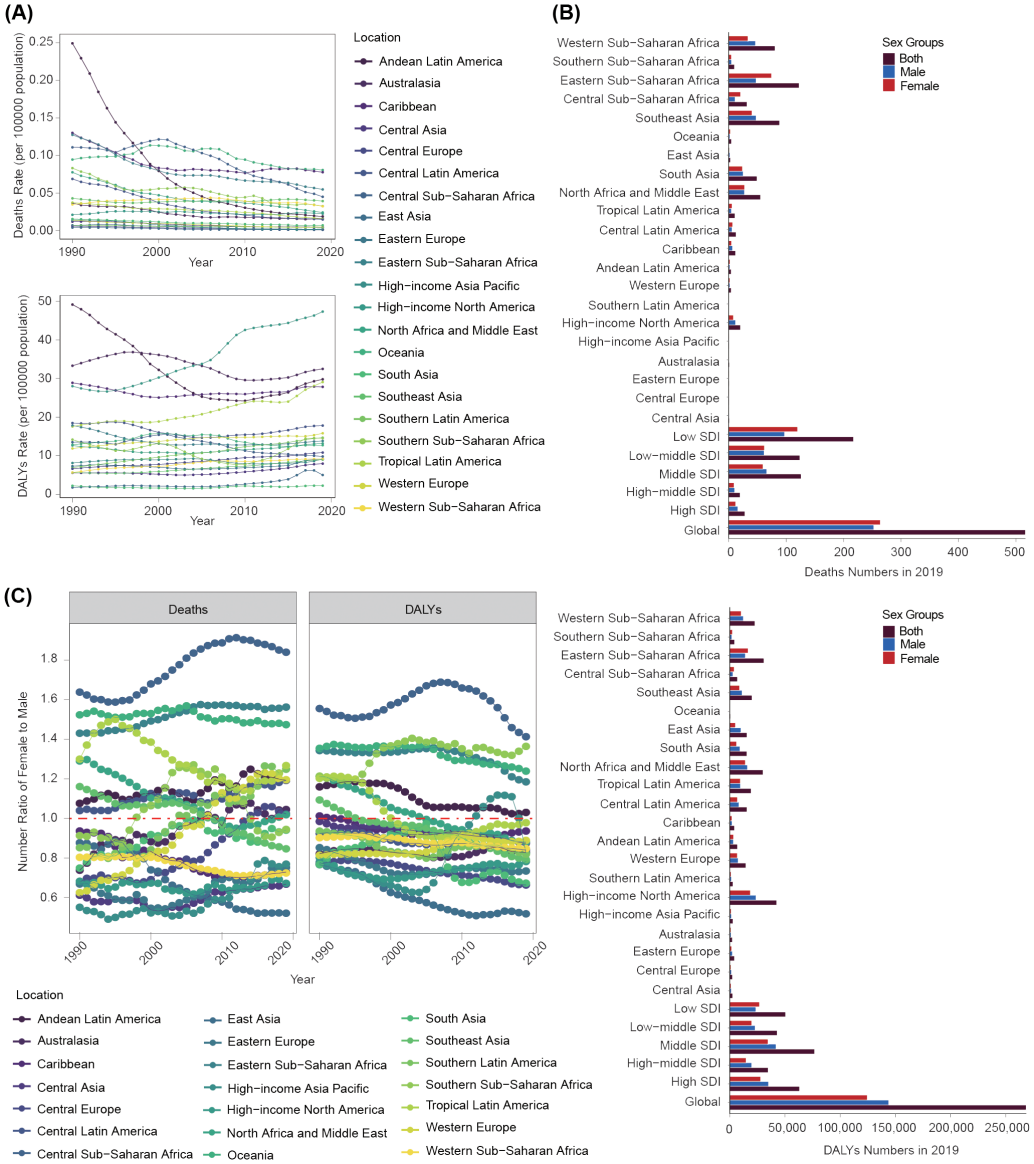
Disease burden linked to a high BMI among children and adolescents aged under 20 years at a regional level. **(A)** Deaths and DALYs rate trends from 1990 to 2019. **(B)** Deaths and DALYs numbers of both males and females in 2019. **(C)** Number ratio of female to male for both deaths and DLYAs from 1990 to 2019.

The number and rate of deaths remained generally stable or decreased across 21 geographical regions from 1990 to 2019 (**Table 1**; **Supplementary Table S1**; **Figure 2A**; **Supplementary Figure S5**). The most significant decline occurred in High-income Asia Pacific (a decrease of 94%; 95% UI: 91% to 96%), despite an upward trend in High income North America, Oceania, and Western Sub-Saharan Africa, and the most prominent increase was observed in Western Sub-Saharan Africa (an increase of 115%; 95% UI: 52% to 213%) (**Supplementary Figure S5**, **Supplementary Table S1**). Over this period, deaths rates decreased in all regions except Southeast Asia, which showed an upward trend (EAPC=0.13). The swiftest decline took place in High-income Asia Pacific and Andean Latin America, with associated EAPCs of −9.57 and −8.9, respectively (**Figure 2A**; **Supplementary Table S1**). The DALYs number and rate generally displayed a constant or escalating tendency, with the largest increase range of the DALYs number occurring in Western Sub-Saharan Africa (276%; 95% UI: 198% to 371%) and the fastest increase in the DALYs rate taking place in East Asia (EAPC= 3.47; 95% CI: 2.59 to 4.35) (**Figure 2A**; **Supplementary Figure S5**, **Supplementary Table S1**). However, some regions demonstrated a downward trend, with the most significant decrease in Andean Latin America (EAPC= −2.16; 95% CI: −2.72 to −1.59) (**Figure 2A**; **Supplementary Table S1**). By 2019, Eastern Sub-Saharan Africa showed the highest deaths number (123; 95% UI: 57 to 226) and Central Europe and High-income Asia Pacific showed the lowest (**Table 1**, **Figure 2B**). High-income North America had the highest number of DALYs in 2019 (42,566; 95% UI: 19,452 to 76,830) and Oceania had the lowest (776; 95% UI: 383 to 1,389) (**Table 1**, **Figure 2B**).

#### 3.2.2 Gender difference and the corresponding changing trend

We further analyzed gender disparities in disease burden across all 21 geographical regions in 2019. Eastern Sub-Saharan Africa exhibited the highest number of female deaths (75; 95% UI: 31 to 158), whereas High-income Asia Pacific had the lowest (0.2; 95% UI: 0.0 to 0.3) (**Figure 2B**). The highest number of male deaths was observed in Southeast Asia (48; 95% UI: 24 to 76), with the lowest number in Eastern Europe (0.5; 95% UI: 0.3 to 1.0) (**Figure 2B**). Oceania recorded the highest female death rate and YLLs rate, whereas East Asia had the lowest. Caribbean had the highest male death rate and YLLs rate, with East Asia having the lowest (**Supplementary Figures S6**, **S8**). High-income North America registered the highest male and female DALYs numbers (18,680; 95% UI: 8,498 to 34,866 for females; 23,885; 95% UI: 10,426 to 44,417 for males), and Oceania had the lowest (430; 95% UI: 188 to 822 for females; 347; 95% UI: 154 to 619 for males) (**Figure 2B**). The female and male DALYs and YLDs rates were highest in High-income North America, with the lowest in South Asia (**Supplementary Figures S6**, **S8**).

The ratio of F/M for deaths and YLLs numbers demonstrated a consistent increasing trend. The ratio of F/M for DALYs and YLDs numbers exhibited a consistent decreasing trend, with more than half of the regions exhibiting a markedly higher proportion of males (F/M <1) (**Figure 2C**; **Supplementary Figure S9**). The F/M ratio of death numbers in High-income Asia Pacific was the lowest. East Asia exhibited the lowest F/M ratio of DALYs number. Notably, over this period, disease burden among females in Central Sub-Saharan Africa was consistently 1.5 times that of males (**Figure 2C**).

#### 3.2.3 Age difference and the corresponding changing trend

**Figure 3:**
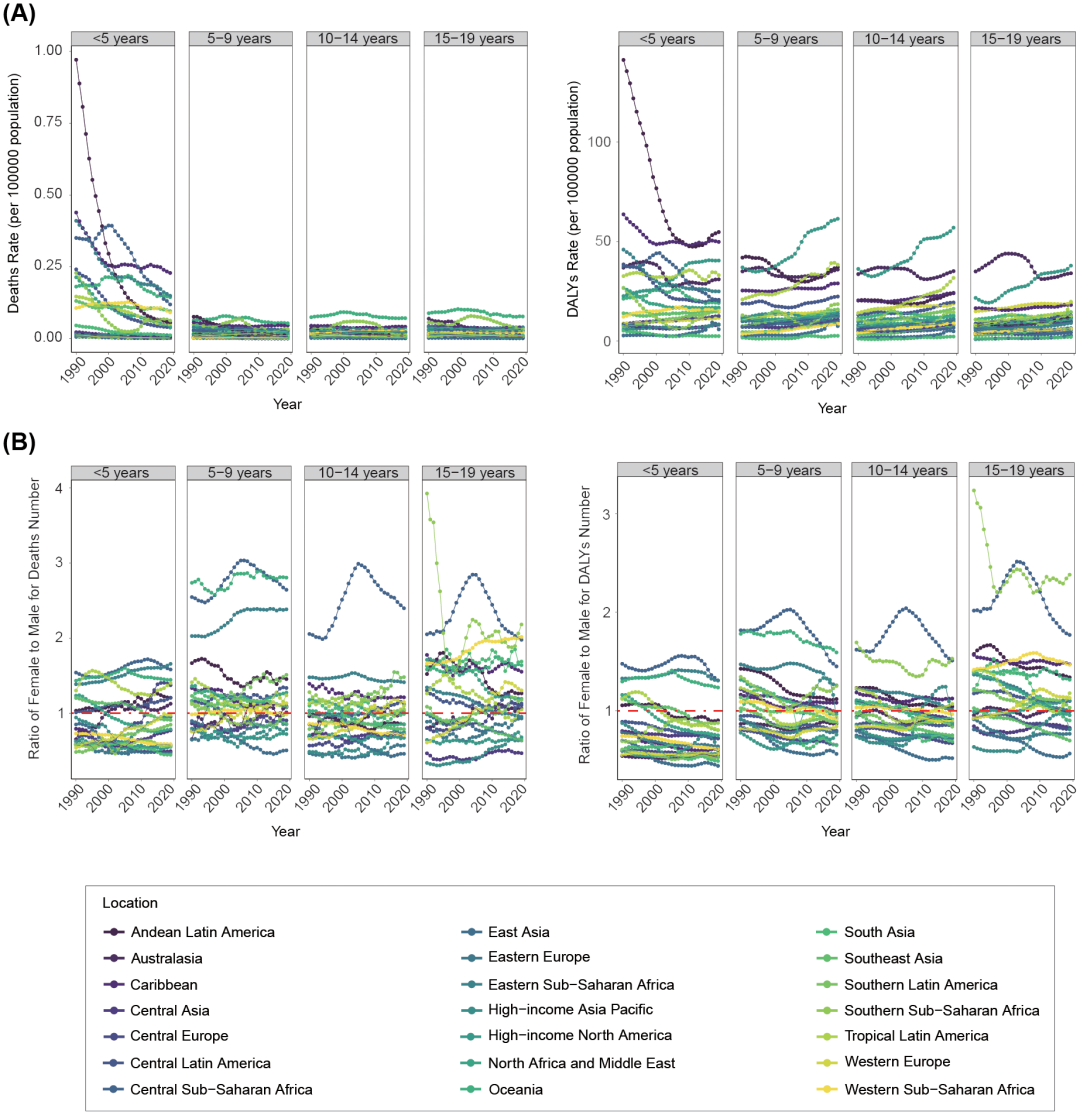
Changing trends of disease burden linked to a high BMI among children and adolescents from 1990 to 2019 at a regional level by age groups. **(A)** Deaths and DALYs rate. **(B)** Number ratio of female to male for both deaths and DALYs.

The highest high-BMI-related burden was found in children aged 0–4 years, although the disease burden in this age group shown rapid declining trend in most regions. It was worth noting that the disease burden in the 0–4-year-old group mainly decreased or fluctuated, but in the other three age groups, the burden of DALYs and YLDs showed a significant upward trend (**Figure 3A**; **Supplementary Figures S7**, **S10**).

We further analyzed gender difference in disease burden across age groups. Generally, disease burden in Central Sub-Saharan Africa was predominately concentrated in females across all age groups, fluctuating at high levels (**Figure 3B**). In children aged 0–4 years, the disease burden was more skewed toward males in most geographical regions, although it was mainly concentrated in females in Central Sub-Saharan Africa, Oceania, Eastern Sub-Saharan Africa, and Andean Latin America. For the 5–9-year-old and 10–14-year-old groups, gender differences in the overall geographical disease burden distribution were not prominent. However, the 15–19-year-old group exhibited a greater female disease burden in most geographical regions (**Figure 3B**; **Supplementary Figure S11**).

### 3.3 National trend of disease burden due to a high BMI in children and adolescents

#### 3.3.1 Overall trend

**Figure 4:**
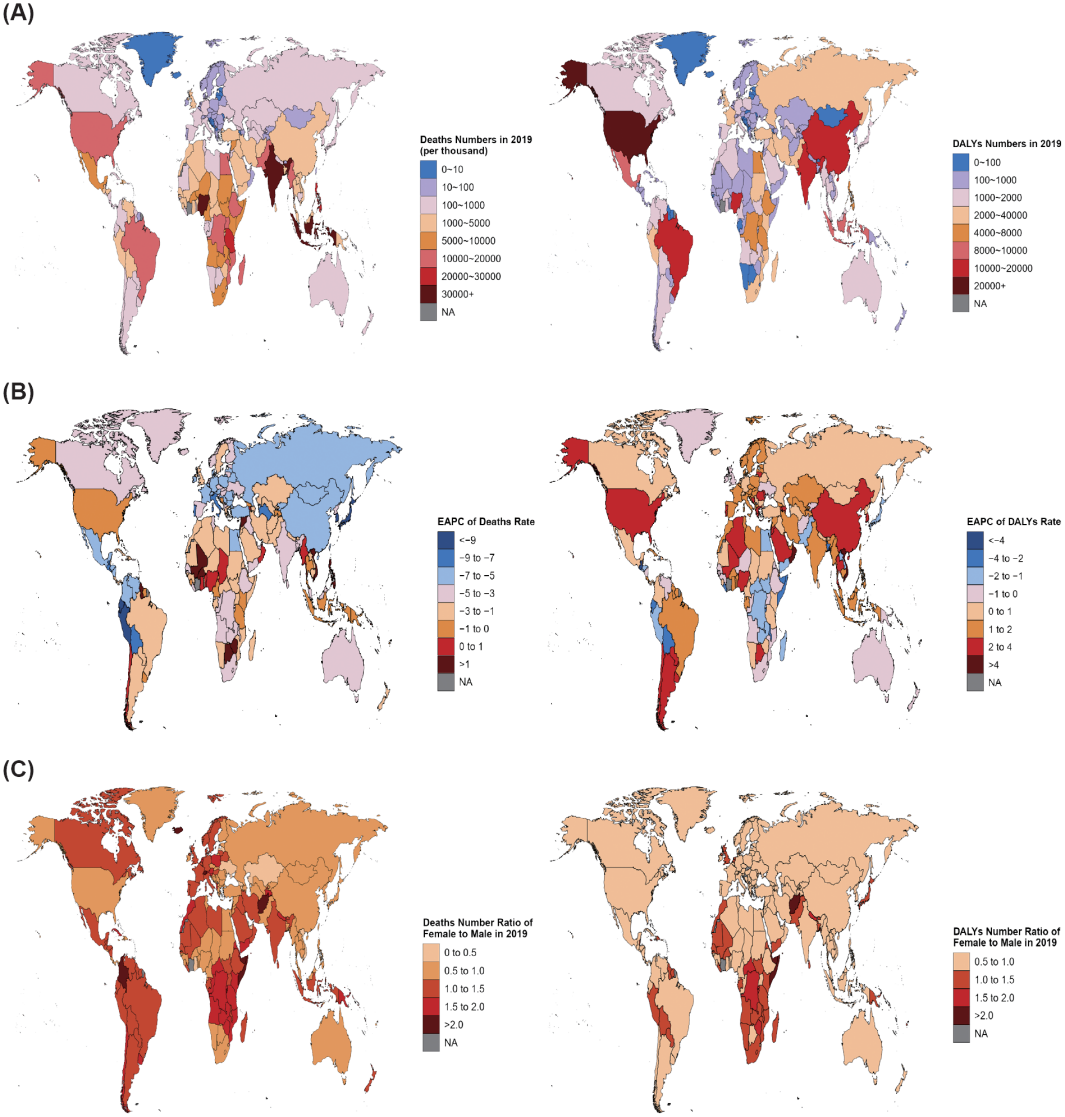
Global map of disease burden attributable to a high BMI among children and adolescents aged under 20 years from 1990 to 2019. **(A)** Deaths and DALYs numbers in 2019. **(B)** EAPC of deaths and DALYs rates from 1990 to 2019. **(C)** Number ratio of female to male for both deaths and DALYs in 2019.

The highest number of deaths in 2019 occurred in Indonesia (36; 95% UI: 19 to 59) (**Figure 4A**; **Supplementary Table S4**). From 1990 to 2019, 165 countries showed decreased numbers of deaths, of which the Republic of Korea had the greatest reduction (decreased 95%; 95% UI: 90% to 97%). Thirty-nine countries showed increased deaths, with the largest increase in Mali (increased 307%; 95% UI: 129% to 686%) (**Supplementary Figure S12**, **Supplementary Table S4**). In terms of mortality rates, 180 countries experienced a downward trend, of which the fastest decline was in Japan (EAPC=−9.76; 95% CI: −10.23 to −9.3). Twenty-four countries experienced an upward trend, with the fastest increase in Dominica (EAPC= 2.34; 95% CI: 1.88 to 2.81) (**Figure 4B**; **Supplementary Table S4**). By 2019, the highest death rate was found in Haiti (0.14; 95% UI: 0.04 to 0.3). Notably, the highest PAF of a high BMI was found in the Syrian Arab Republic at 96.2 (95% UI: 45.72 to 156.5), whereas the lowest was in Tajikistan at 0.19 (95% UI: 0.08 to 0.38) (**Supplementary Table S4**).

In 2019, the highest DALYs number was in the USA (40,829; 95% UI: 18,700 to 73,685) (**Figure 4A**; **Supplementary Table S5**). In 161 countries, the DALYs experienced an upward trend from 1990 to 2019, with the highest in Mali (increased 546%; 95% UI: 309% to 784%). Forty-three countries decreased, with the biggest decrease in Japan (decreased 47%; 95% UI: 37% to 56%) (**Supplementary Figure S13**, **Supplementary Table S5**). Regarding the DALYs rate, 149 countries showed an increasing trend, and Oman had the fastest increase in DALYs rate (EAPC= 5.95; 95% CI: 5.66 to 6.24). Fifty-five countries showed a downward trend, and Guatemala recorded the largest decrease (EAPC= −4.35; 95% CI: −5.29 to −3.39) (**Figure 4B**; **Supplementary Table S5**). Generally, the global DALYs rate in 2019 was 10.41 (95% UI: 4.87 to 18.67), with 136 countries above the global average and 68 countries below it (**Supplementary Table S5**).

#### 3.3.2 Gender difference and the corresponding changing trend

Subsequently, we conducted an analysis on the sex ratio (F/M) of deaths and DALYs numbers in 204 countries, which is shown in **Figure 4C**. In 2019, 104 countries worldwide registered higher death numbers among females than among males (F/M >1). Conversely, 100 countries had a higher male population than female (F/M <1). The country with the highest number of female deaths was Grenada (F/M = 3.09), and Palau had the highest number of male deaths (F/M = 0.25). Fifty-three countries reported higher numbers of DALYs among females than among males (F/M >1), whereas 151 countries had a male majority (F/M <1). The nation with the highest proportion of female DALYs was Afghanistan (F/M = 2.26), and Slovakia had the highest proportion of males (F/M = 0.48).

### 3.4 SDI regional burden due to a high BMI in children and adolescents

#### 3.4.1 Overall trend

**Figure 5:**
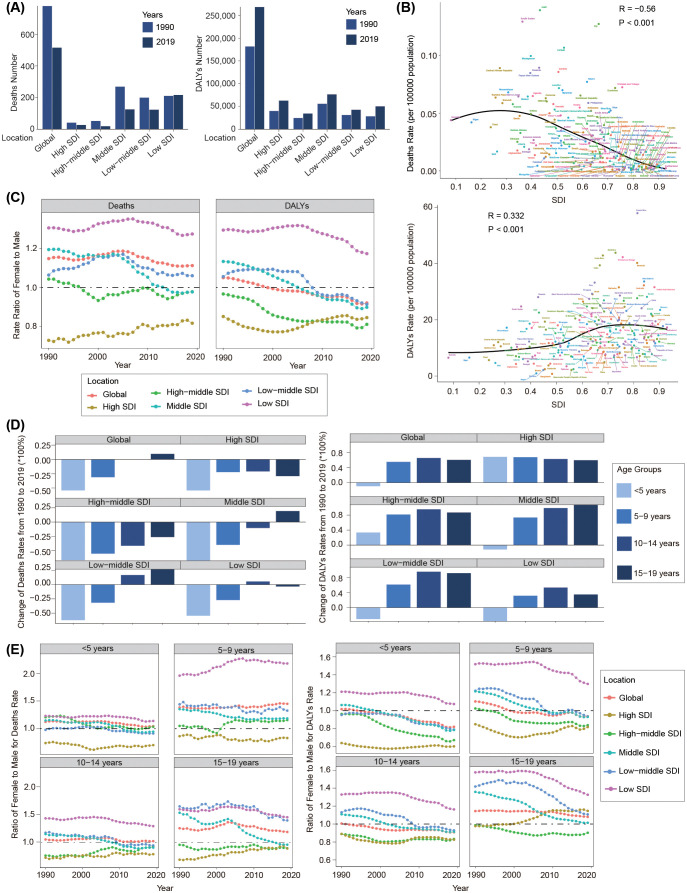
Disease burden associated with a high BMI among children and adolescents aged under 20 years from 1990 to 2019 by SDI regions. **(A)** Deaths and DALYs numbers in 1990 and 2019. **(B)** Correlation between deaths and DALYs rates with SDI. **(C)** Ratio of female to male for both deaths and DALYs rate from 1990 to 2019. **(D)** Change of rates from 1990 to 2019 in SDI regions by age groups. **(E)** Ratio of female to male for both deaths and DALYs rate in SDI regions by age groups from 1990 to 2019.

Overall, compared with 1990, the number of deaths among children and adolescents linked to a high BMI in 2019 decreased in all SDI regions, except in the low SDI region. The high and high-middle SDI regions were generally lower than the other three SDI regions (**Table 1**, **Figure 5A**). In 1990, the middle SDI region exhibited the highest number of deaths (270; 95% UI: 132 to 431), whereas the high SDI region demonstrated the lowest (42; 95% UI: 24 to 361). By 2019, the lowest number occurred in the high-middle SDI region (20; 95% UI: 11 to 31), and the low SDI region had the highest (217; 95% UI: 107 to 376) (**Table 1**, **Figure 5A**). The death rate showed a declining trend across all SDI regions, with the fastest decrease in the high-middle SDI region (EAPC=−2.9; 95% CI: −3.06 to −2.75). Over the three decades, the death rate in the low SDI region was the highest and the high-middle SDI region had the lowest rate (**Supplementary Figure S14**, **Supplementary Table S1**). We further explored the relationship between SDI values and the death rate in the 204 countries in 2019. Results revealed that the death rate was negatively related to the SDI values (Pearson r = −0.56; *P*<0.01) (**Figure 5B**).

Compared with 1990, the DALYs number, on the contrary, increased across all five SDI regions in 2019, with the highest in the middle SDI region (76,755; 95% UI: 35,168 to 140,785) and the lowest in the high-middle SDI region (34,803; 95% UI: 15,281 to 66,078) (**Table 1**, **Figure 5A**). Although the DALYs rate decreased in the low SDI region (EAPC= −0.43; 95% UI: −0.6 to −0.26), it increased in most SDI regions, with the fastest rate in the high SDI region (EAPC= 1.95; 95% UI: 1.86 to 2.03). Notably, from 1990 to 2019, the high SDI region always had the highest DALYs rate (**Supplementary Figure S14**, **Supplementary Table S1**). Pearson correlation analysis revealed that the DALYs rate was positively related to the SDI values (Pearson r =0.332, *P*<0.001) (**Figure 5B**).

#### 3.4.2 Gender difference and the corresponding changing trend

From 1990 to 2019, the proportions of both sexes remained stable for death burden across all the five SDI regions and was mainly concentrated in females in the low SDI region and concentrated in males in the high SDI region (**Figure 5C**). Females exhibited a higher DALYs burden than males in the low SDI region, whereas males contributed more in other SDI regions. Over this period, the DALYs burden changed to males across all the five SDI regions (**Figure 5C**; **Supplementary Figure S15**).

#### 3.4.3 Age difference and the corresponding changing trend

We further explored the changing trends in disease burden among various age groups in the five SDI regions from 1990 to 2019. Generally, death rates in children under 10 years of age showed a downward trend in all five SDI regions. The deaths rate among children aged 10–14 years decreased in the high, high-middle, and middle SDI regions, but increased in the low-middle and middle SDI regions. In the 15–19-year-old group, the death rate decreased in the high, high-middle, and low SDI regions, but increased in the middle and low-middle SDI regions (**Figure 5D**). The DALYs rate showed an increasing trend in all the five SDI regions in people aged over 5 years old, but showed a decreasing trend in children aged 0–4 years in the middle, low-middle, and low SDI regions (**Figure 5D**). The YLDs rate increased in all the age groups across all the five SDI regions, but generally showed a decreasing trend in most age groups (**Supplementary Figure S16**).

We further investigated the relationship between F/M ratios and SDI levels in different age groups. As is shown in **Figure 5E**, the overall trend indicated that as the SDI level increased, the proportion of males in the death rate and DALYs rate increased. In the low SDI region, deaths and the DALYs burden due to a high BMI across all the age groups were mainly concentrated in females; however, in the high SDI region, the disease burden was mainly concentrated in males, except the DALYs rate in the 15–19-year-old group.

### 3.5 The influential factors for EAPC

We then conducted Pearson correlation analysis to explore the influencing factors of the EAPC. The rates of disease burden associated with a high BMI in 1990 served as a baseline disease library, and the human development index (HDI) in 2019 could be utilized as an index for each country’s healthcare level. As shown in **Supplementary Figure S17A** in the Supplement, the relationship between the death rate and the EAPC was positive when the death rate was under 0.1 per 100,000 people (Pearson r = 0.23; *P*=0.003), but negative when the death rate was over 0.1 per 100,000 people (Pearson r =−0.436; *P*=0.009). The DALYs and YLDs rates showed a significant negative correlation with their respective EAPCs (Pearson r = −0.541, *P*<0.001 for DALYs; Pearson r = −0.485, *P*<0.001 for YLDs). Similar to the death burden, the relationship between EAPC and the YLLs rate was also an inverted V-shape. The EAPC was positively related to the YLLs rate when the YLLs rate was below 8 per 100,000 people (Pearson r = 0.246; *P* =0.001), but negatively related when the YLLs rate was above 8 per 100,000 people (Pearson r=−0.424; *P*=0.009).

There was a notable negative correlation between EAPCs and the HDI in terms of deaths and YLLs (Pearson r=−0.354, *P*<0.001 for deaths; Pearson r=−0.343, *P*<0.001 for YLLs). In contrast, the EAPC for DALYs was positively related to the HDI (Pearson r = 0.363; *P*<0.001). A significant negative association was detected between the EAPC and YLDs rate (Pearson r =−0.239; *P*=0.011) when the YLDs rate was over 0.7 per 100,000 people, but this association disappeared when the YLDs rate was below 0.7 per 100,000 people (**Supplementary Figure S17B**).

## 4 Discussion

Over the past three decades, overweight and obesity among children and adolescents worldwide has become a major public health problem due to its increasing medical and social costs. Here, we investigated shifting trends in the disease burden associated with a high BMI among children and adolescents aged 0 to 19 years in all GBD regions and countries from 1990 to 2019. Our study found that the younger the individual, the greater the disease burden associated with a high BMI, which may be due to differences in physical resilience and tolerance. With advancing age, individuals exhibited an increasingly robust ability to resist disease pressure. In 1990, females suffered a much higher burden than males due to a high BMI. However, the burden of disease associated with a high BMI varied across age groups by sex, with a gradual increase in males at all life stages. The reasons for this phenomenon remain incompletely understood, albeit similarities can be drawn with the gender disparity in the disease burden due to a high BMI in adults (15, 16).

In addition, based on the 2019 analysis of correlations between disease burden and SDI levels in countries, we found that the higher the SDI level, the lower the death rate. Furthermore, the largest reduction in related death occurred in the middle-high SDI region, which may be related to enhanced medical services, allowing diseases to be diagnosed earlier and treated better. In contrast, areas with a low SDI or low-income countries have a limited capacity to identify high-risk groups and provide timely interventions, potentially resulting in adverse outcomes and high deaths. Given the complex non-linear relationship between overweight and obesity with SDI, the temporal trend in the high BMI-related disease burden cannot be explained solely by income level, but is also related to education level and the prevalence of local health policies.

Our study underscored the importance of a high BMI as a risk factor for increased deaths and DALYs among children and adolescents. However, it is crucial to acknowledge the multifactorial nature of disease causation, in which a high BMI may interact with several other potential confounding factors to influence health outcomes. The relationship between the BMI and health outcomes is likely modified by a complex interplay of socioeconomic, environmental, behavioral, and biological factors that may vary across populations and over time. First, socioeconomic status plays a significant role, with a lower socioeconomic status being associated with higher rates of obesity and poorer health outcomes, possibly due to limited access to healthy food options and recreational facilities. A study in pediatrics has demonstrated the impact of socioeconomic factors on childhood obesity, emphasizing the need for targeted interventions in vulnerable populations (17). Second, there are the influences of biological factors, encompassing genetics, epigenetics, and neuroendocrine factors. Many studies have identified genetic variations associated with childhood obesity. For example, genetic susceptibility, in conjunction with genes such as *FTO*, affects appetite regulation and energy expenditure, ultimately leading to obesity (18). Although obesity has a high heritability, the genetic variants identified to date account for only a small proportion of the phenotypic variation. The origin of the obesity epidemic cannot be fully explained by genetic factors alone. In recent years, epigenetic research has provided valuable insights into the dramatic increase in global obesity rates. Existing evidence indicates that environmental exposures can induce changes in the epigenome, leading to the intergenerational transmission of obesity risk (19). The endocrine and nervous systems interact to manage individual physiological functions and maintain internal balance. The nervous system regulates hormone release through the hypothalamus, participates in various metabolic processes, and is influenced by feedback mechanisms involving neurons from different brain regions, environmental factors, peripheral organs, and mediators. Abnormalities in neuroendocrine pathways are associated with the development of pediatric metabolic syndrome, which includes conditions such as visceral obesity, insulin resistance, type 2 diabetes, and hypertension (20). Finally, changes in dietary patterns, such as an excessive intake of high-calorie foods, a decrease in physical activity, and an increase in screen time, are also other drivers of weight gain, leading to an increase in the BMI (21–24). Overweight and obesity were considered major risk factors for diabetes, cardiovascular disease, and kidney disease, along with their respective complications (25, 26). The escalating prevalence of overweight and obesity in children and adolescents worldwide necessitates the development of a consistent habit of monitoring individuals’ BMI from a young age. Early intervention in behavioral health can yield significant health benefits, as changes in risk factors for unhealthy behaviors during childhood may help mitigate or counteract the rise in obesity and overweight.

The combined effect of a high BMI with these factors may lead to synergistic increases in health risks, beyond what would be expected from each factor alone. For instance, the impact of a high BMI on cardiovascular diseases may be more pronounced in individuals with a sedentary lifestyle or poor dietary habits. It is important to recognize that the causal pathways linking a high BMI to adverse health outcomes are not unidirectional and can be influenced by bidirectional relationships and feedback loops. For example, chronic diseases may, in turn, affect an individual’s weight and physical activity levels. Future studies should aim to disentangle the complex interactions between the BMI and other risk factors by employing longitudinal study designs and advanced statistical methods. This will provide a more nuanced understanding of the pathways through which a high BMI contributes to the global burden of disease in children and adolescents.

This study presented global estimates across nearly three decades to assess the efficacy of interventions. Public health policymakers, decision-makers, and relevant stakeholders should design and implement evidence-based interventions and allocate appropriate financial support, aimed at raising awareness of the potential health risks associated with overweight and obesity, while promoting physical activity (27).

The study has some limitations. First, the analysis relied on the GBD database. For some countries or regions, the data may be estimated based on a subset of samples and not fully representative of the real world of the entire country or region studied. Second, our primary focus was on a risk factor, not the specific disease type. Data on the incidence and prevalence of overweight and obesity were therefore not available, and the specific data of the BMI were not available, either. Third, there may be bias when the definition of overweight and obesity used the BMI. The BMI is a widely accepted indicator for assessing overweight and obesity. It has inherent limitations that must be considered when interpreting our findings. The BMI is calculated as weight in kilograms divided by the square of height in meters (kg/m²), and it does not differentiate between fat mass and lean mass, such as muscle. This can lead to misclassification, particularly among individuals with high muscle mass but low fat, who may be incorrectly categorized as overweight or obese. Additionally, the BMI does not account for the distribution of fat, which is an important factor in health outcomes; for example, central obesity (excess fat around the waist) is more strongly associated with health risks than peripheral fat. And finally, the GBD study provides a comprehensive dataset for estimating the burden of diseases and risk factors, including a high BMI. However, establishing causality from observational data such as those in the GBD is inherently challenging. The GBD relies on statistical associations and modeling to estimate the burden associated with several risk factors, including a high BMI. Although these methods are robust and widely used, they cannot definitively prove causality. There may be confounding factors not accounted for in the models that could influence the observed associations. The apparent association between a high BMI and disease burden could be due to the influence of other health conditions on the BMI rather than the BMI causing the health outcomes. To address these limitations, future research should incorporate more nuanced measures of body composition and consider longitudinal studies that can better elucidate the temporal relationships between BMI, health behaviors, and disease outcomes. Additionally, further investigation into the biological mechanisms linking a high BMI to specific health conditions is needed to strengthen the causal inference.

## 5 Conclusion

In this study, we noted that the global death numbers of children and adolescents attributed to a high BMI has declined somewhat between 1990 and 2019, but the disability burden substantially increased. The disease burden had the greatest impact on children under 5 years of age. The death burden among females was higher than that of males, whereas since 1999, the disability burden suffered by males has surpassed that of females. Over the years, the DALYs increased in all the five SDI regions, and the deaths decreased except in the low SDI region. In future, targeted measures should be formulated and implemented according to each country’s specific development context to prevent and reduce the burden of multiple diseases in advance.

## Data Availability

The original contributions presented in the study are included in the article/**Supplementary Material**. Further inquiries can be directed to the corresponding author.
