# Supplementary material for: The global death and disability burden associated with a high BMI in children and adolescents, 1990–2019
Source: Front Endocrinol (Lausanne). 2024 Oct 8;15:1463002. doi: 10.3389/fendo.2024.1463002 (PMC11493657; doi:10.3389/fendo.2024.1463002)
Supplement: Supplementary file 1 [file DataSheet1.pdf]

Supplement to: Global death and disability burden of overweight and obesity in children and adolescents, 1990-2019

Table of Contents

Supplementary Figure 1. Numbers of deaths and disability-adjusted life years (DALYs) attribuTable to high body mass index (BMI) among children and adolescents aged under 20 years from 1990 to 2019 by sex. *3*

Supplementary Figure 2. Rates of deaths and disability-adjusted life years (DALYs) attribuTable to high body mass index (BMI) among children and adolescents aged under 20 years from 1990 to 2019 by age group. *4*

Supplementary Figure 3. Rates of years lived with disability (YLDs) and years of life lost prematurely (YLLs) attribuTable to high body mass index (BMI) among children and adolescents aged under 20 years from 1990 to 2019 by sex. YLDs, years lived with disability; YLLs, years of life lost prematurely; BMI, body mass index *5*

Supplementary Figure 4. Numbers of years lived with disability (YLDs) and years of life lost prematurely (YLLs) attribuTable to high body mass index (BMI) among children and adolescents aged under 20 years in 2019 by sex and age group. YLDs, years lived with disability; YLLs, years of life lost prematurely; BMI, body mass index *6*

Supplementary Figure 5. Numbers of deaths and disability-adjusted life years (DALYs) attribuTable to high body mass index (BMI) among children and adolescents aged under 20 years from 1990 to 2019 by geographical region *7*

Supplementary Figure 6. Rates of deaths and disability-adjusted life years (DALYs) attribuTable to high body mass index (BMI) among children and adolescents aged under 20 years in 2019 by sex and region. *8*

Supplementary Figure 7. Numbers of deaths and disability-adjusted life years (DALYs) attribuTable to high body mass index (BMI) among children and adolescents aged under 20 years from 1990 to 2019 by geographical region *9*

Supplementary Figure 8. Rates of years lived with disability (YLDs) and years of life lost prematurely (YLLs) attribuTable to high body mass index (BMI) among children and adolescents aged under 20 years in 2019 by sex and region. *10*

Supplementary Figure 9. Number ratio of females to males for years lived with disability (YLDs) and years of life lost prematurely (YLLs) among children and adolescents aged under 20 years from 1990 to 2019 by geographical region *11*

Supplementary Figure 10. Numbers of years lived with disability (YLDs) and years of life lost prematurely (YLLs) among children and adolescents aged under 20 years in 2019 by age group and region. 12

Supplementary Figure 11. Number ratio of females to males for years lived with disability (YLDs) and years of life lost prematurely (YLLs) among children and adolescents aged under 20 years from 1990 to 2019 by age group and region........ 13

Supplementary Figure 12. Global map of deaths numbers change percent among children and adolescents aged under 20 years from 1990 to 2019. 14

Supplementary Figure 13. Global map of disability-adjusted life years (DALYs) numbers change percent among children and adolescents aged under 20 years from 1990 to 2019........ 15

Supplementary Figure 14. Rates of deaths and disability-adjusted life years (DALYs) attribuTable to high body mass index (BMI) among children and adolescents aged under 20 years from 1990 to 2019 by sociodemographic index (SDI) region......... 16

Supplementary Figure 15. Numbers of deaths and disability-adjusted life years (DALYs) attribuTable to high body mass index (BMI) among children and adolescents aged under 20 years in 2019 by sex and sociodemographic index (SDI) region......... 17

Supplementary Figure 16. Change percent of years lived with disability (YLDs) and years of life lost prematurely (YLLs) rates attribuTable to high body mass index (BMI) among children and adolescents aged under 20 years in 2019 by age group and sociodemographic index (SDI) region. 18

Supplementary Figure 17. The relationship between estimated annual percentage change (EAPC) with deaths rate, disability-adjusted life years (DALYs) rate, the years lived with disability (YLDs) and the years of life lost (YLLs) in 1990 (A) or with human development index (HDI) in 2019 (B)......... 19

Supplementary Table 1. Death rate and DALYs rate attribuTable to high BMI in children and adolescents at Global, SDI and geographical regional level from 1990 to 2019........ 20

Supplementary Table 2. YLLs burden attribuTable to high BMI in children and adolescents at Global, SDI and geographical regional level from 1990 to 2019 22

Supplementary Table 3. YLDs burden attribuTable to high BMI in children and adolescents at Global, SDI and geographical regional level from 1990 to 2019 25

Supplementary Table 4. Death burden attribuTable to high BMI in children and adolescents at national level from 1990 to 2019........ 28

Supplementary Table 5. DALYs burden attribuTable to high BMI in children and adolescents at national level from 1990 to 2019........ 43

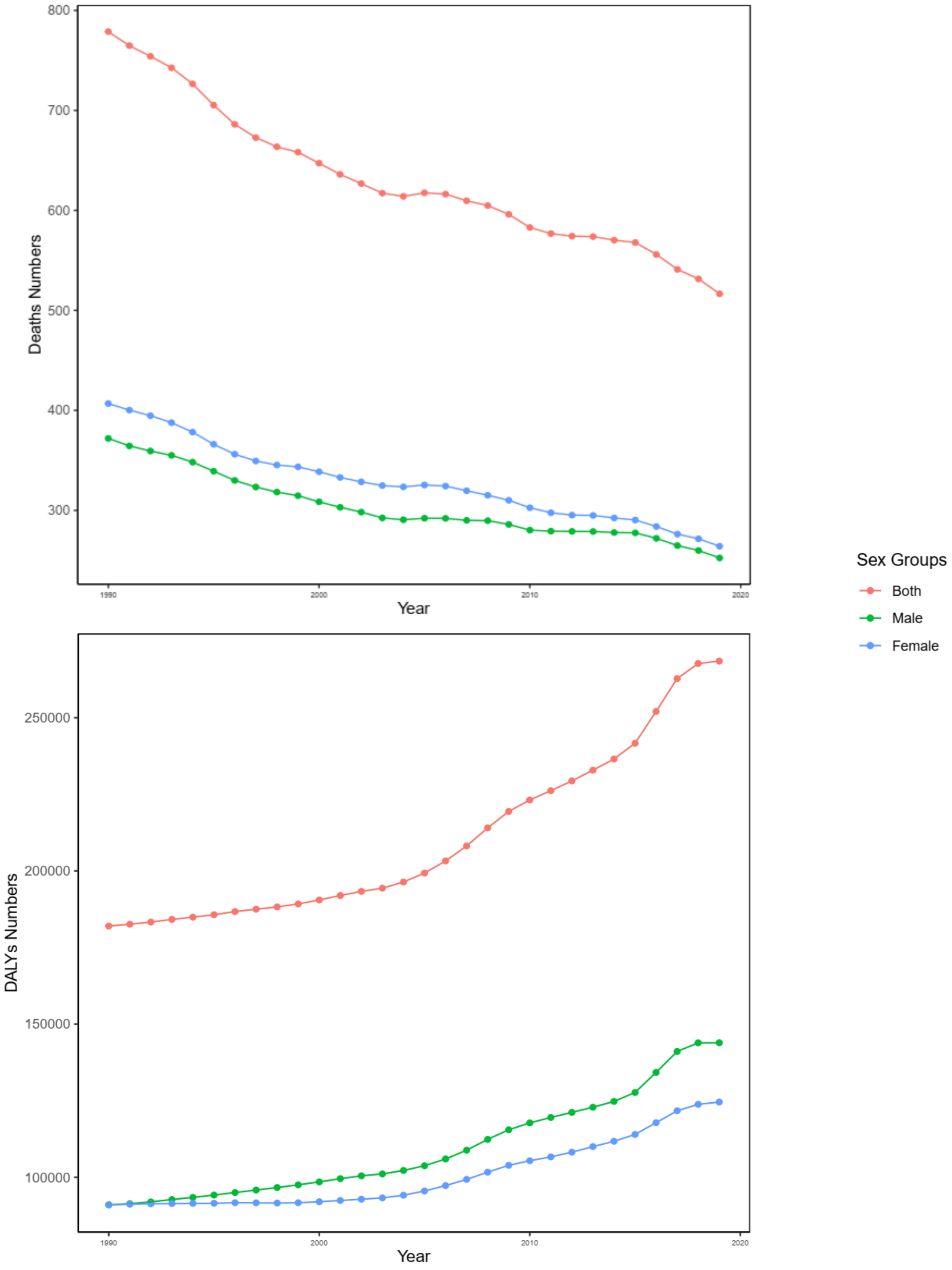

Supplementary Figure 1. Numbers of deaths and disability-adjusted life years (DALYs) attribuTable to high body mass index (BMI) among children and adolescents aged under 20 years from 1990 to 2019 by sex.

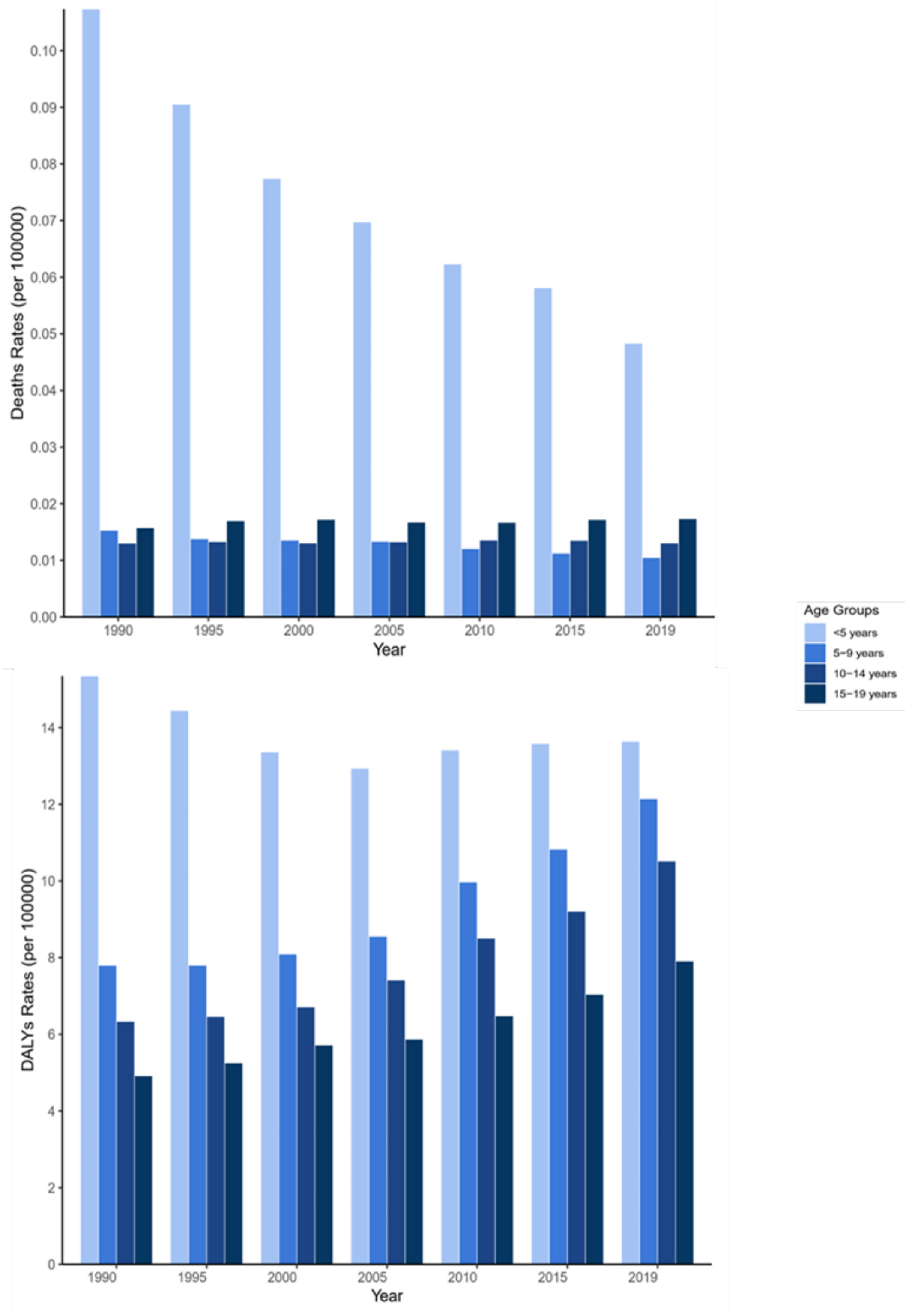

Supplementary Figure 2. Rates of deaths and disability-adjusted life years (DALYs) attribuTable to high body mass index (BMI) among children and adolescents aged under 20 years from 1990 to 2019 by age group.

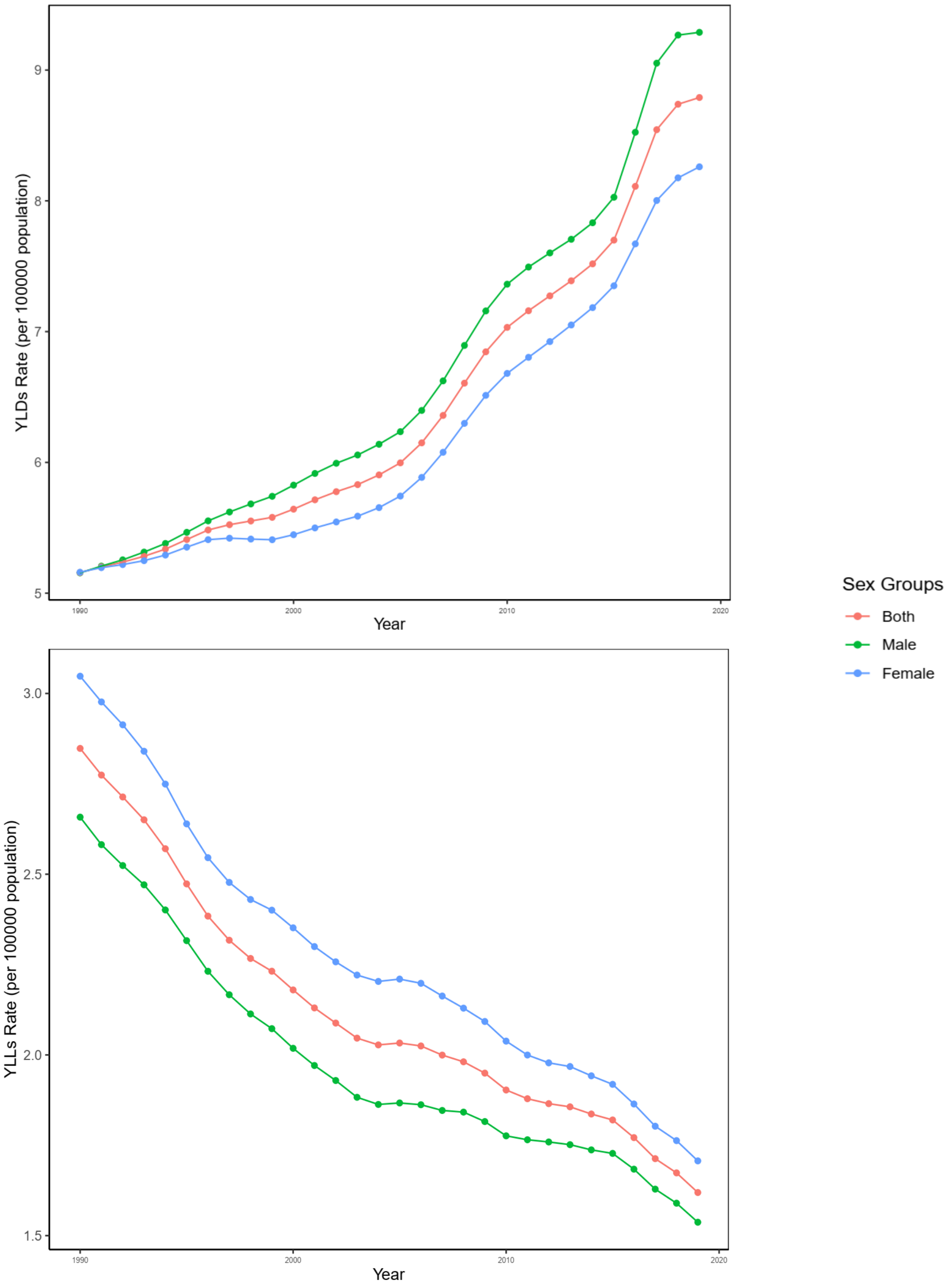

Supplementary Figure 3. Rates of years lived with disability (YLDs) and years of life lost prematurely (YLLs) attribuTable to high body mass index (BMI) among children and adolescents aged under 20 years from 1990 to 2019 by sex. YLDs, years lived with disability; YLLs, years of life lost prematurely; BMI, body mass index

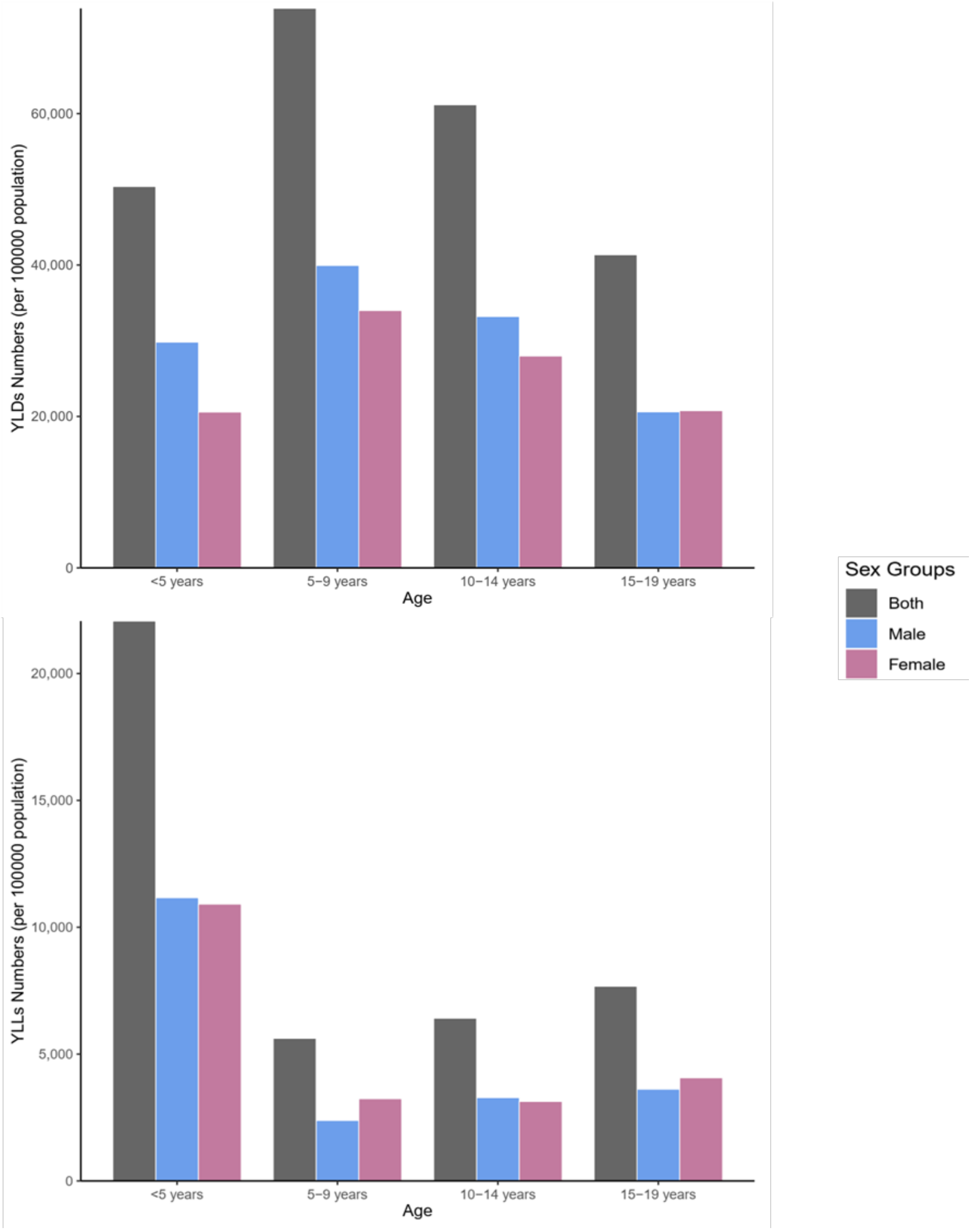

Supplementary Figure 4. Numbers of years lived with disability (YLDs) and years of life lost prematurely (YLLs) attribuTable to high body mass index (BMI) among children and adolescents aged under 20 years in 2019 by sex and age group. YLDs, years lived with disability; YLLs, years of life lost prematurely; BMI, body mass index

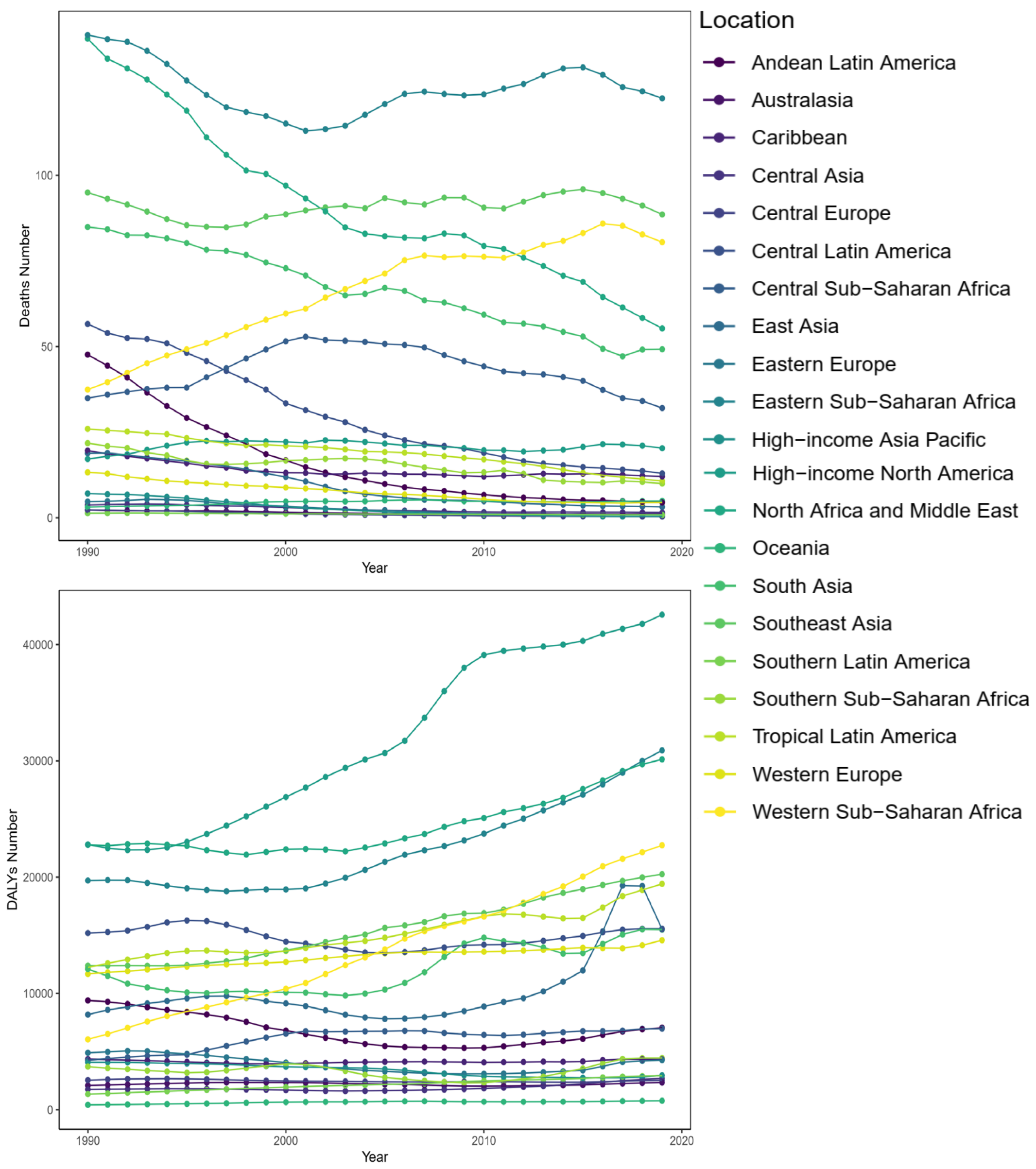

Supplementary Figure 5. Numbers of deaths and disability-adjusted life years (DALYs) attribuTable to high body mass index (BMI) among children and adolescents aged under 20 years from 1990 to 2019 by geographical region.

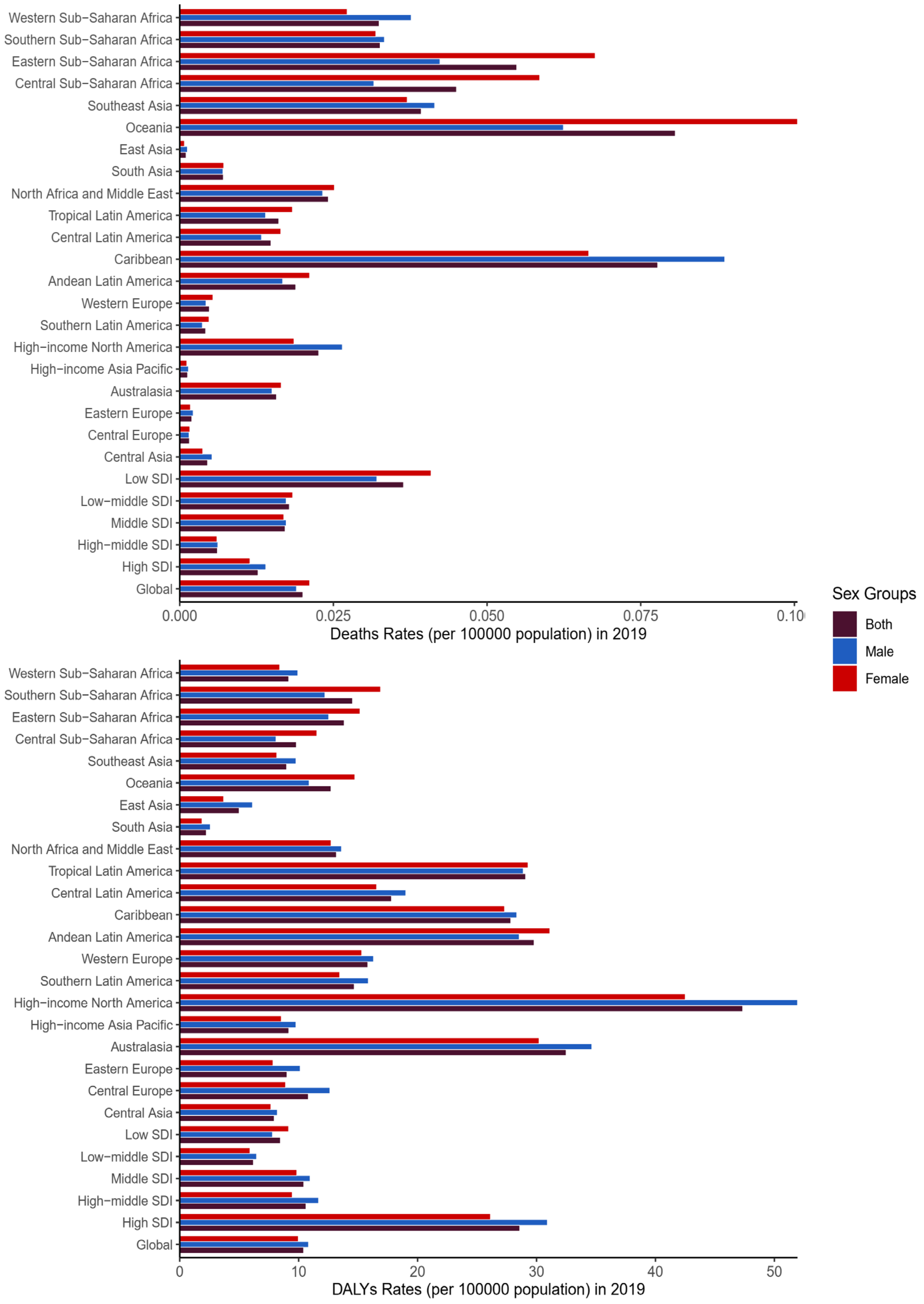

Supplementary Figure 6. Rates of deaths and disability-adjusted life years (DALYs) attribuTable to high body mass index (BMI) among children and adolescents aged under 20 years in 2019 by sex and region.

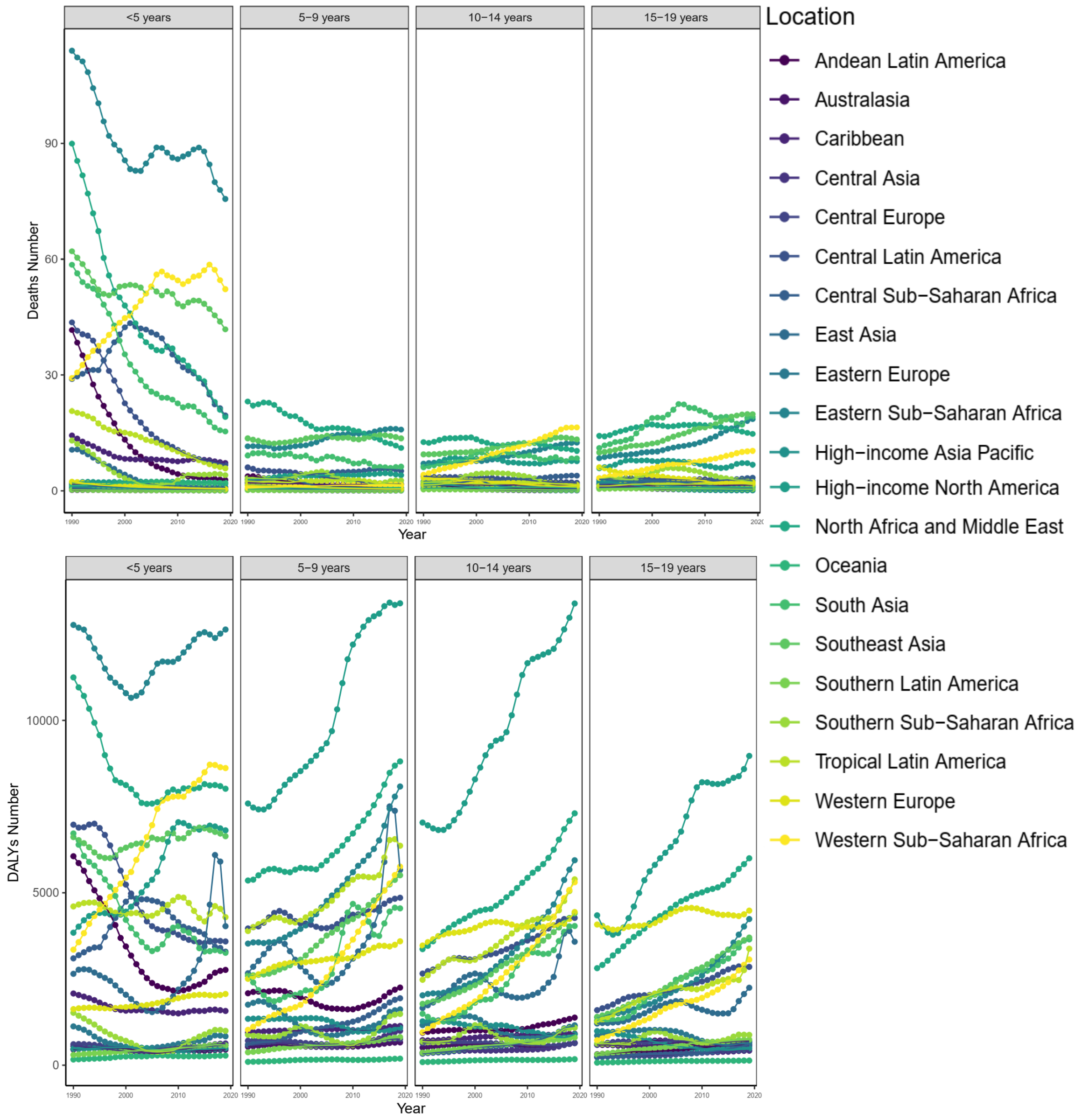

Supplementary Figure 7. Numbers of deaths and disability-adjusted life years (DALYs) attribuTable to high body mass index (BMI) among children and adolescents aged under 20 years from 1990 to 2019 by geographical region.

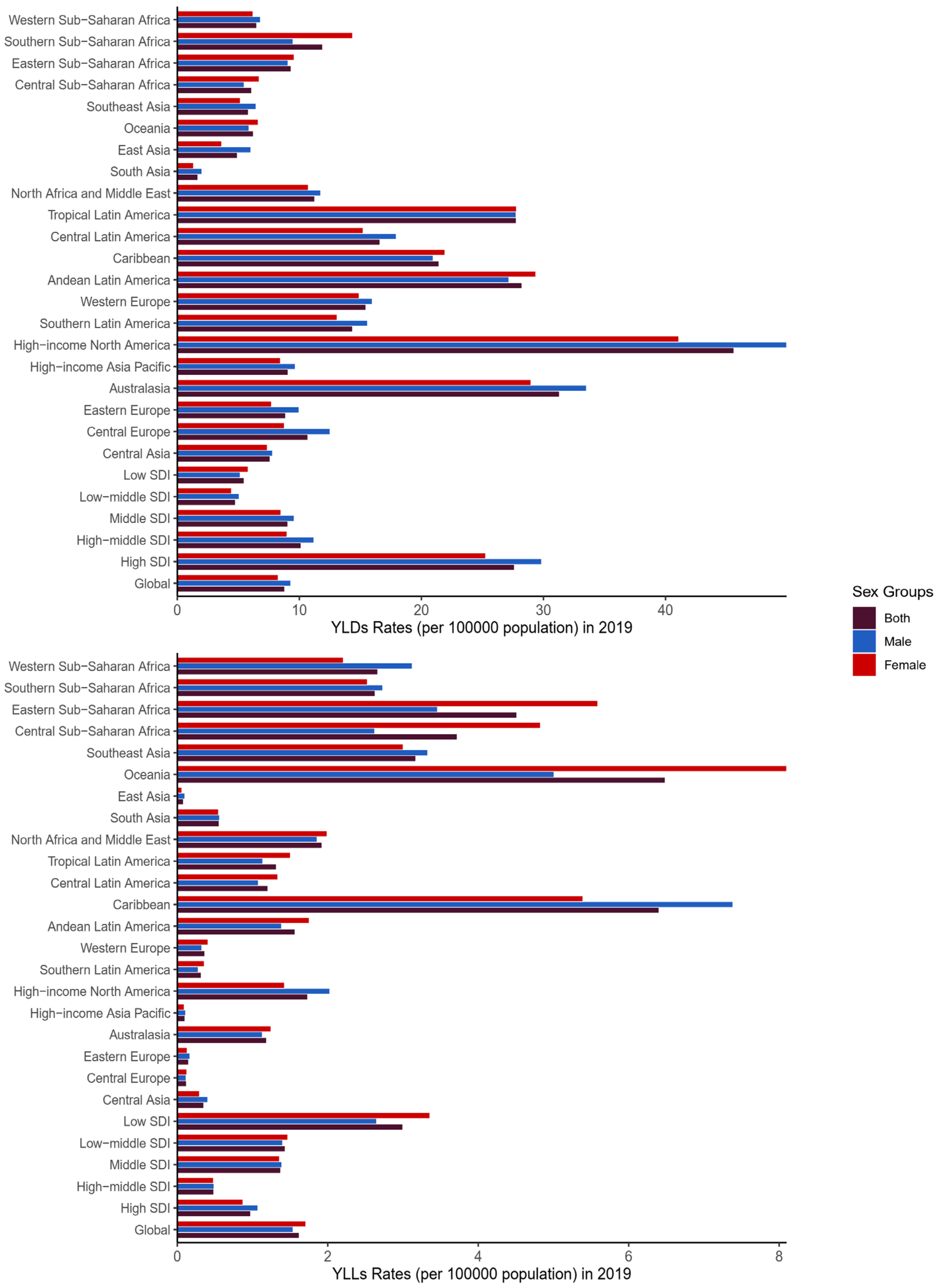

Supplementary Figure 8. Rates of years lived with disability (YLDs) and years of life lost prematurely (YLLs) attribuTable to high body mass index (BMI) among children and adolescents aged under 20 years in 2019 by sex and region.

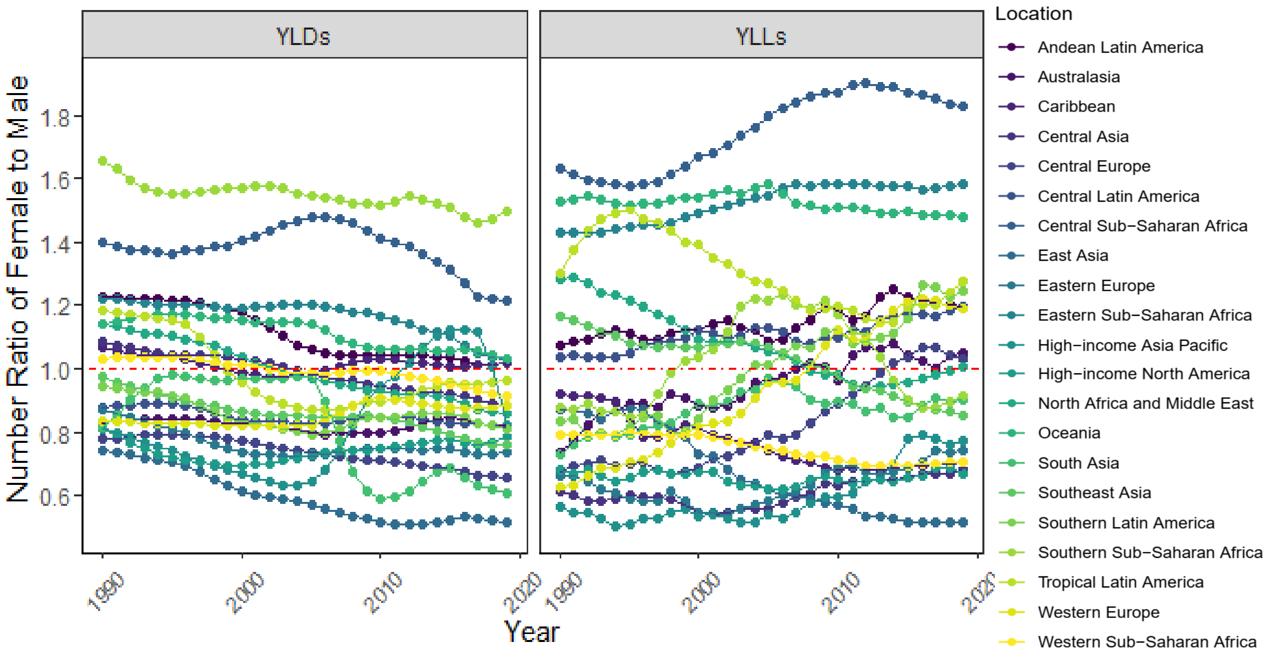

Supplementary Figure 9. Number ratio of females to males for years lived with disability (YLDs) and years of life lost prematurely (YLLs) among children and adolescents aged under 20 years from 1990 to 2019 by geographical region.

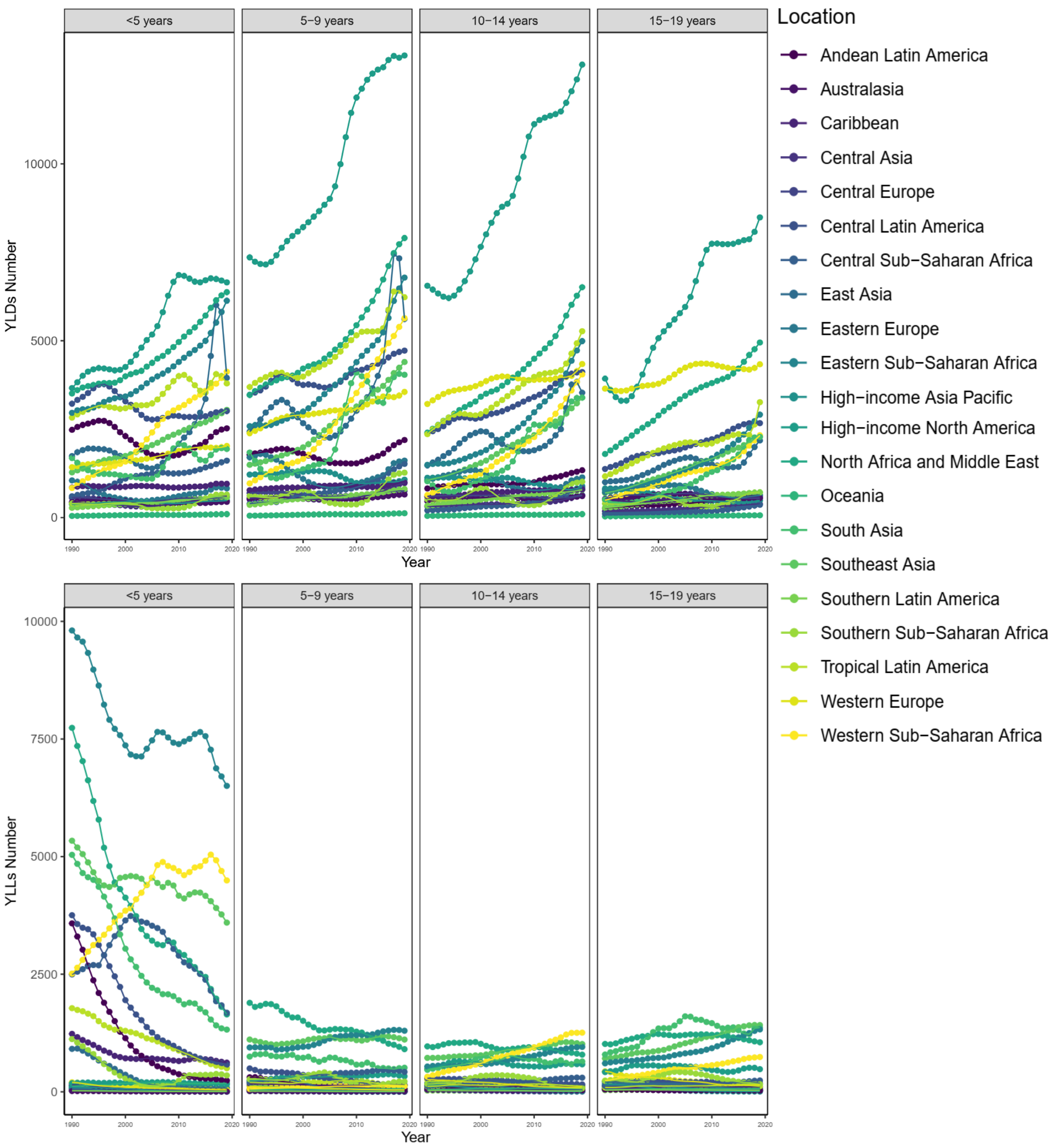

Supplementary Figure 10. Numbers of years lived with disability (YLDs) and years of life lost prematurely (YLLs) among children and adolescents aged under 20 years in 2019 by age group and region.

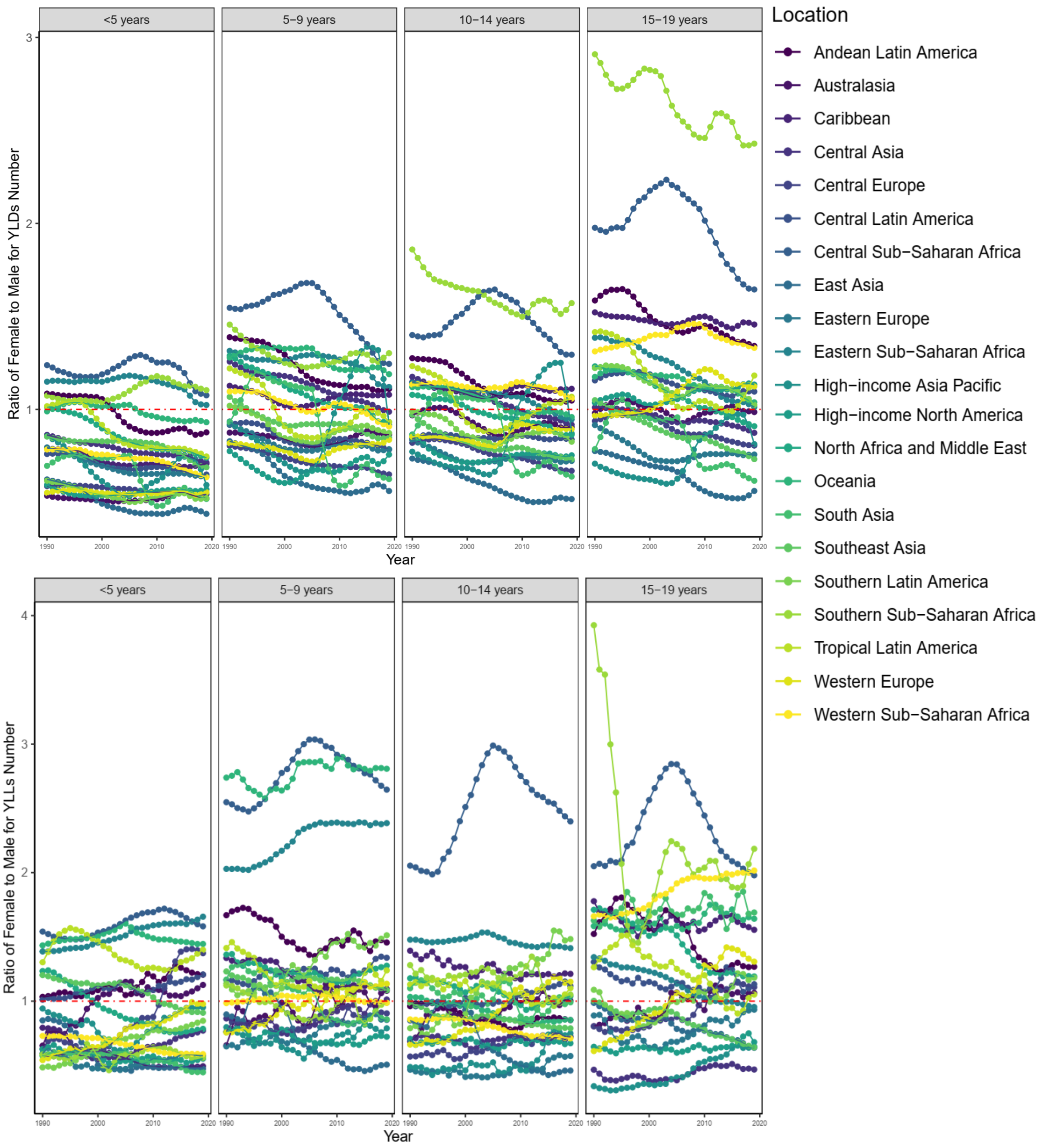

Supplementary Figure 11. Number ratio of females to males for years lived with disability (YLDs) and years of life lost prematurely (YLLs) among children and adolescents aged under 20 years from 1990 to 2019 by age group and region.

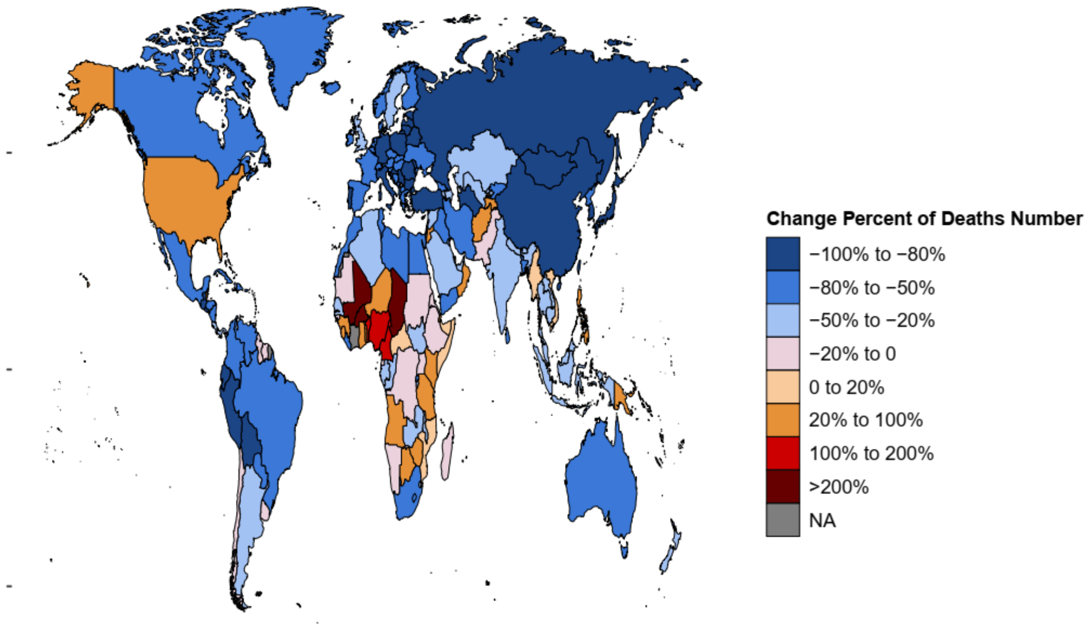

Supplementary Figure 12. Global map of deaths numbers change percent among children and adolescents aged under 20 years from 1990 to 2019.

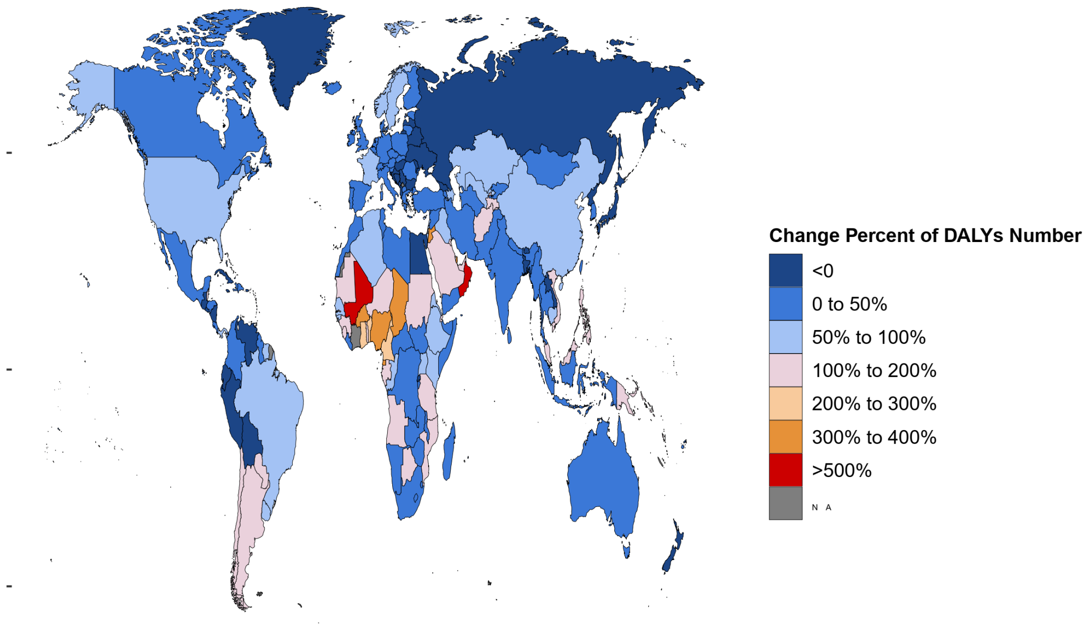

Supplementary Figure 13. Global map of disability-adjusted life years (DALYs) numbers change percent among children and adolescents aged under 20 years from 1990 to 2019.

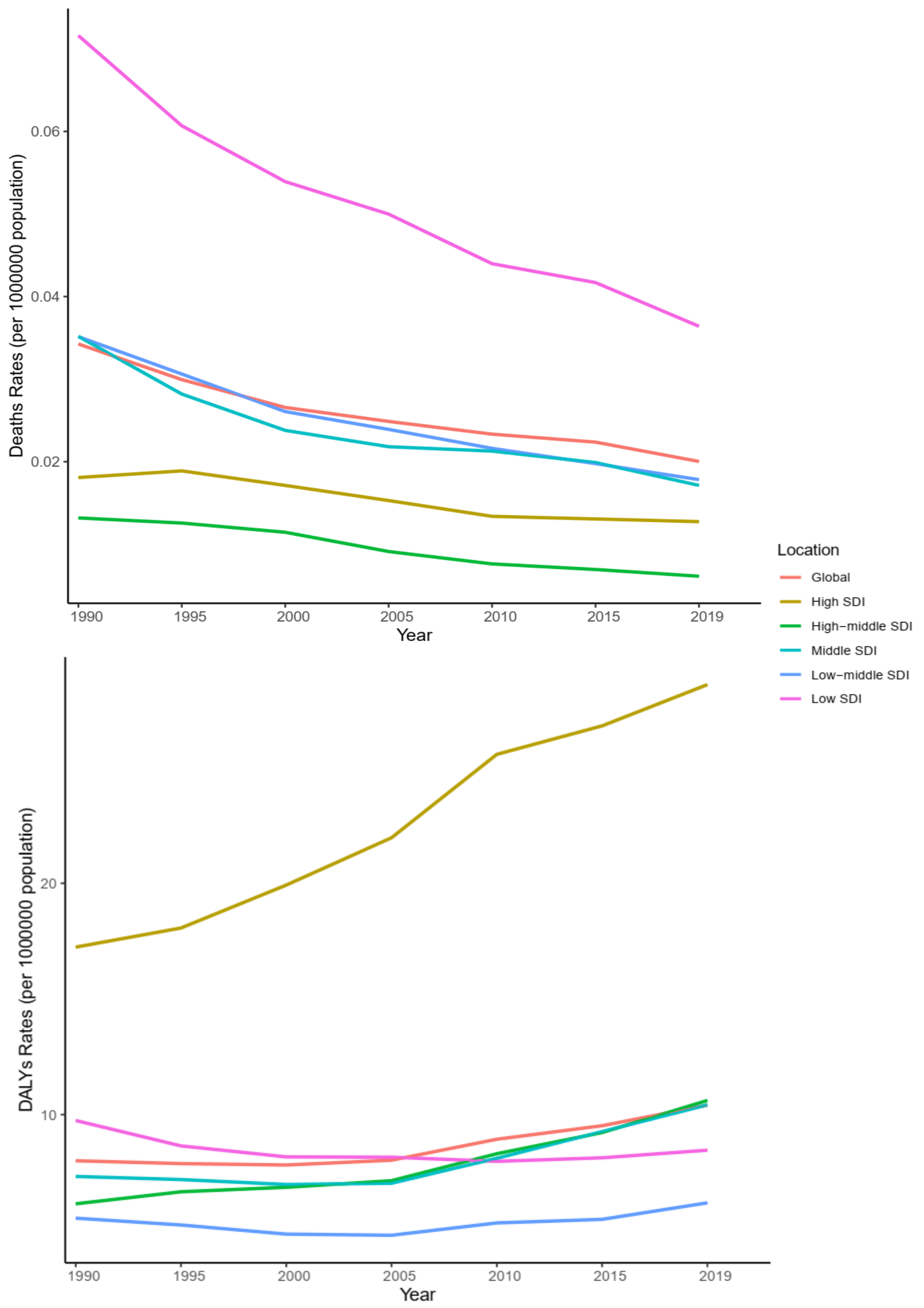

Supplementary Figure 14. Rates of deaths and disability-adjusted life years (DALYs) attribuTable to high body mass index (BMI) among children and adolescents aged under 20 years from 1990 to 2019 by sociodemographic index (SDI) region.

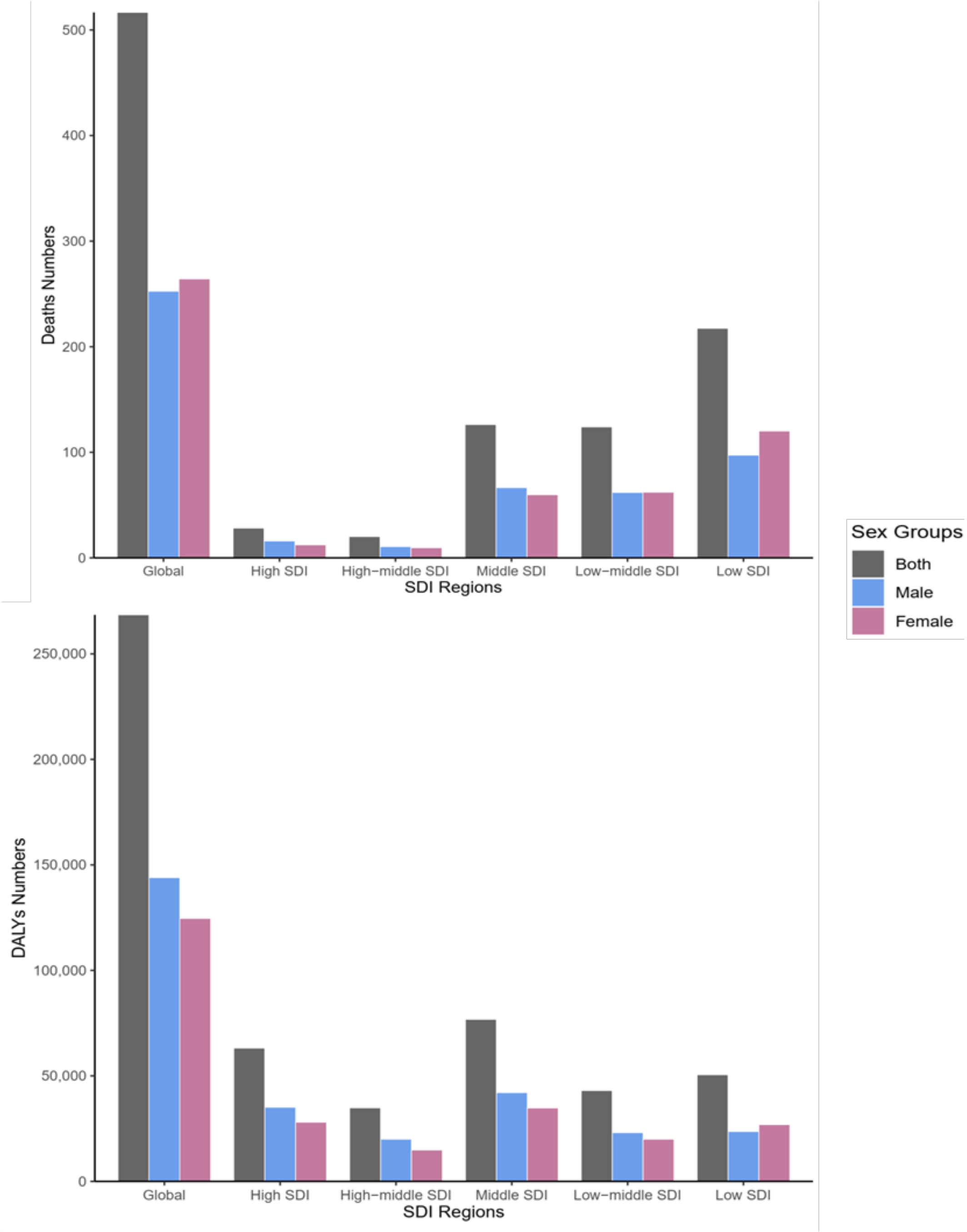

Supplementary Figure 15. Numbers of deaths and disability-adjusted life years (DALYs) attribuTable to high body mass index (BMI) among children and adolescents aged under 20 years in 2019 by sex and sociodemographic index (SDI) region.

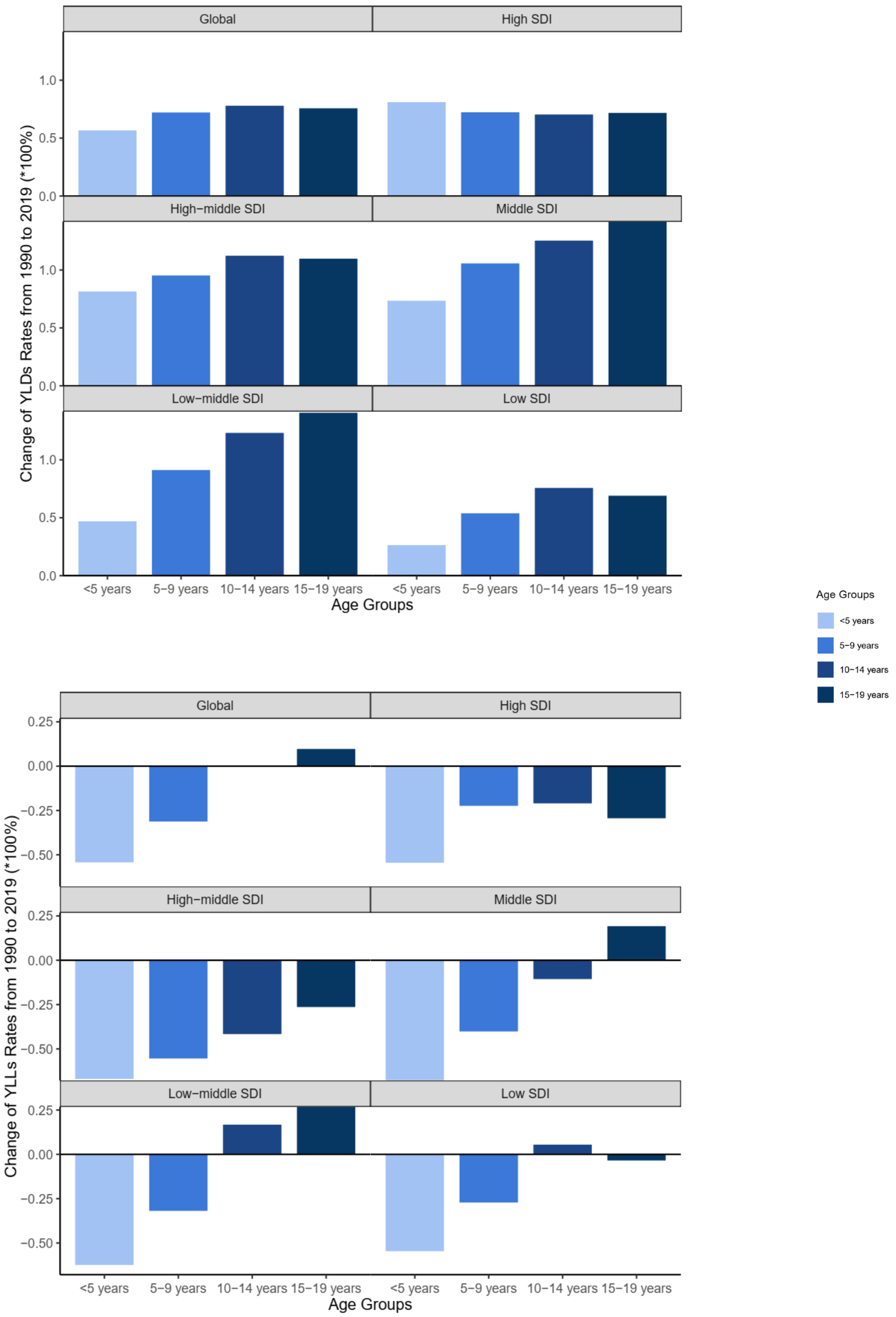

Supplementary Figure 16. Change percent of years lived with disability (YLDs) and years of life lost prematurely (YLLs) rates attribuTable to high body mass index (BMI) among children and adolescents aged under 20 years in 2019 by age group and sociodemographic index (SDI) region.

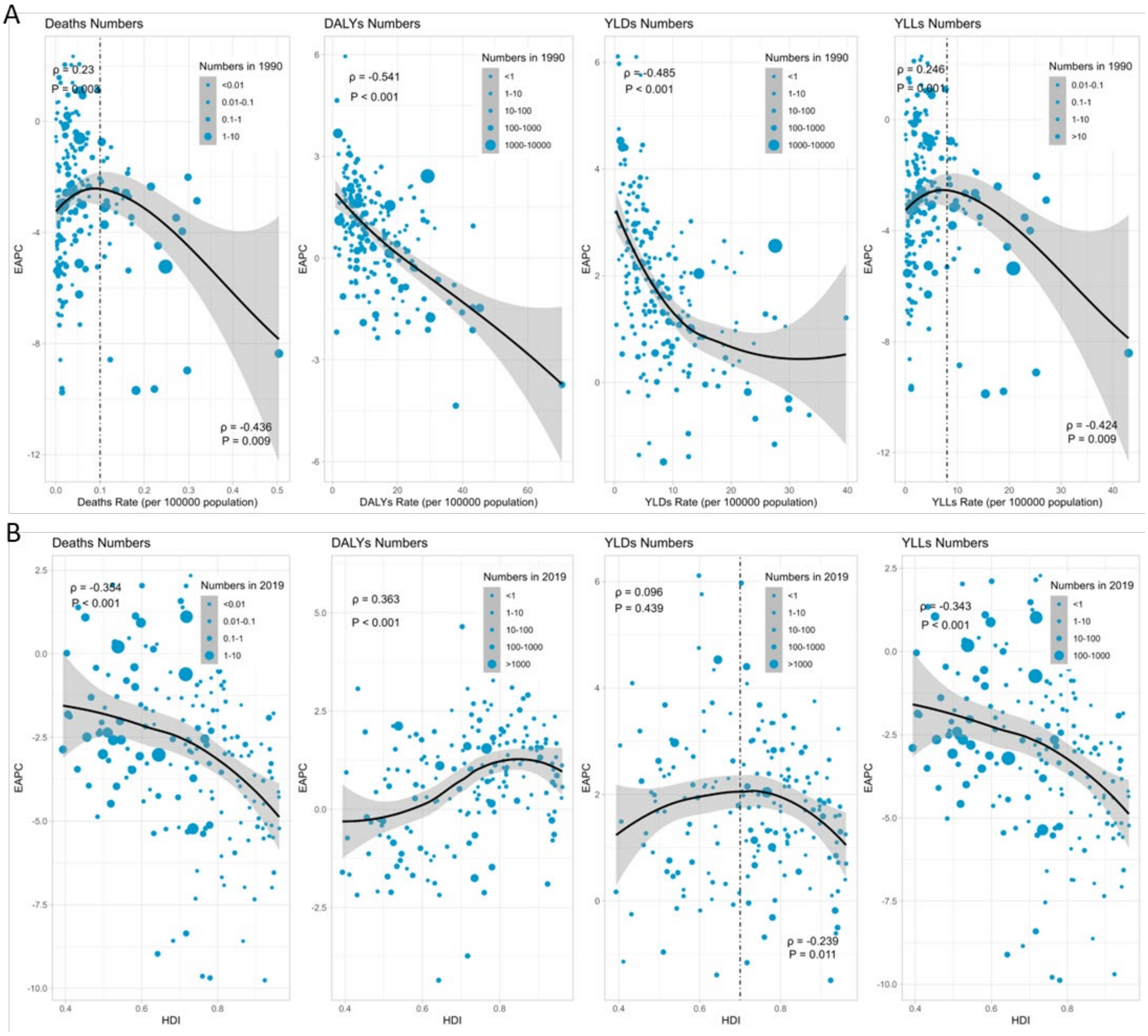

Supplementary Figure 17. The relationship between estimated annual percentage change (EAPC) with deaths rate, disability-adjusted life years (DALYs) rate, the years lived with disability (YLDs) and the years of life lost (YLLs) in 1990 (A) or with human development index (HDI) in 2019 (B).

Supplementary Table 1. Death rate and DALYs rate attribuTable to high BMI in children and adolescents at Global, SDI and geographical regional level from 1990 to 2019

	**Deaths burden**			**DALYs burden**		
**Location**	**Deaths rate in 1990 per 100000 (95%UI)**	**Deaths rate in 2019 per 100000 (95%UI)**	**EAPC of Deaths rate, 1990-2019 (%)**	**DALYs rate in 1990 per 100000 (95%UI)**	**DALYs rate in 2019 per 100000 (95%UI)**	**EAPC of DALYs rate, 1990-2019 (%)**
**Global**	0.03 (0.02 to 0.06)	0.02 (0.01 to 0.03)	-1.62 (-1.74 to -1.5)	8.01 (4.02 to 13.75)	10.41 (4.87 to 18.67)	0.98 (0.79 to 1.17)
**SDI**						
High SDI	0.02 (0.01 to 0.03)	0.01 (0.01 to 0.02)	-1.6 (-1.77 to -1.43)	17.24 (7.8 to 32.48)	28.58 (13.11 to 53.1)	1.95 (1.86 to 2.03)
High-middle SDI	0.01 (0.01 to 0.02)	0.01 (0 to 0.01)	-2.9 (-3.06 to -2.75)	6.14 (2.85 to 11.25)	10.61 (4.66 to 20.15)	1.85 (1.63 to 2.08)
Middle SDI	0.04 (0.02 to 0.06)	0.02 (0.01 to 0.03)	-2 (-2.24 to -1.76)	7.33 (3.67 to 12.52)	10.42 (4.77 to 19.11)	1.3 (0.97 to 1.63)
Low-middle SDI	0.04 (0.02 to 0.06)	0.02 (0.01 to 0.03)	-2.21 (-2.31 to -2.11)	5.52 (2.69 to 9.25)	6.19 (2.94 to 10.9)	0.38 (0.1 to 0.65)
Low SDI	0.07 (0.03 to 0.13)	0.04 (0.02 to 0.06)	-2.12 (-2.22 to -2.01)	9.75 (4.51 to 16.63)	8.46 (4.2 to 14.63)	-0.43 (-0.6 to -0.26)
**Regions**						
Central Asia	0.01 (0.01 to 0.02)	0 (0 to 0.01)	-3.92 (-4.18 to -3.65)	5.55 (2.55 to 10.22)	7.94 (3.34 to 15.31)	1.11 (0.72 to 1.51)
Central Europe	0.01 (0 to 0.01)	0 (0 to 0)	-5.09 (-5.28 to -4.89)	6.61 (2.87 to 12.66)	10.81 (4.55 to 21.1)	1.66 (1.61 to 1.71)
Eastern Europe	0.01 (0 to 0.01)	0 (0 to 0)	-5.64 (-6.03 to -5.23)	7.28 (3.11 to 13.96)	9.01 (3.77 to 18.06)	0.29 (-0.07 to 0.65)
Australasia	0.04 (0.02 to 0.05)	0.02 (0.01 to 0.02)	-3.18 (-3.52 to -2.84)	33.3 (15.35 to 59.79)	32.48 (14.89 to 63.32)	-0.67 (-0.88 to -0.45)
High-income Asia Pacific	0.01 (0.01 to 0.02)	0 (0 to 0)	-9.57 (-10.02 to -9.11)	8.1 (3.66 to 15.25)	9.17 (3.84 to 17.8)	-0.23 (-0.44 to -0.01)
High-income North America	0.02 (0.01 to 0.03)	0.02 (0.01 to 0.03)	-0.27 (-0.52 to -0.01)	28.01 (12.21 to 53.86)	47.33 (21.63 to 85.42)	2.34 (2.14 to 2.53)
Southern Latin America	0.01 (0 to 0.01)	0 (0 to 0.01)	-1.73 (-1.82 to -1.64)	6.94 (3.11 to 13.04)	14.67 (6.46 to 29.07)	2.56 (2.47 to 2.65)
Western Europe	0.01 (0.01 to 0.02)	0 (0 to 0.01)	-3.94 (-4.12 to -3.75)	11.87 (5.44 to 22.14)	15.81 (7.23 to 30.26)	0.84 (0.74 to 0.93)
Andean Latin America	0.25 (0.1 to 0.47)	0.02 (0.01 to 0.04)	-8.9 (-9.39 to -8.41)	49.15 (22.11 to 86.12)	29.79 (12.96 to 58.99)	-2.16 (-2.72 to -1.59)
Caribbean	0.13 (0.05 to 0.25)	0.08 (0.03 to 0.15)	-1.4 (-1.78 to -1.03)	28.88 (13.65 to 50.96)	27.83 (12.91 to 50.55)	-0.03 (-0.2 to 0.13)
Central Latin America	0.07 (0.03 to 0.11)	0.01 (0.01 to 0.03)	-5.6 (-5.88 to -5.32)	18.46 (8.89 to 32.26)	17.8 (7.94 to 34.06)	-0.4 (-0.72 to -0.07)

Tropical Latin America	0.04 (0.02 to 0.07)	0.02 (0.01 to 0.03)	-2.57 (-2.78 to -2.35)	17.56 (7.95 to 31.82)	29.08 (12.77 to 55)	1.59 (1.42 to 1.75)
North Africa and Middle East	0.08 (0.04 to 0.13)	0.02 (0.01 to 0.04)	-3.49 (-3.72 to -3.26)	12.64 (6.47 to 21.02)	13.17 (6.07 to 23.86)	0.26 (-0.01 to 0.52)
South Asia	0.02 (0.01 to 0.03)	0.01 (0 to 0.01)	-2.87 (-2.97 to -2.77)	2.21 (1.01 to 3.82)	2.23 (1.01 to 4.09)	0.64 (0.15 to 1.13)
East Asia	0 (0 to 0.01)	0 (0 to 0)	-5.37 (-5.8 to -4.93)	1.76 (0.78 to 3.31)	4.99 (2.02 to 9.94)	3.47 (2.59 to 4.35)
Oceania	0.09 (0.04 to 0.17)	0.08 (0.04 to 0.15)	-0.79 (-1.15 to -0.43)	12.83 (6.31 to 21.61)	12.71 (6.28 to 22.74)	-0.37 (-0.69 to -0.04)
Southeast Asia	0.04 (0.02 to 0.07)	0.04 (0.02 to 0.06)	0.13 (-0.02 to 0.29)	5.6 (2.7 to 9.45)	8.98 (4.47 to 15.69)	1.9 (1.76 to 2.05)
Central Sub-Saharan Africa	0.11 (0.03 to 0.24)	0.05 (0.02 to 0.09)	-2.98 (-3.56 to -2.4)	13.5 (5.04 to 26)	9.8 (4.72 to 17.59)	-1.35 (-1.71 to -0.99)
Eastern Sub-Saharan Africa	0.13 (0.05 to 0.23)	0.05 (0.03 to 0.1)	-2.56 (-2.83 to -2.29)	17.81 (8.21 to 30)	13.82 (6.76 to 24.08)	-0.8 (-1.1 to -0.5)
Southern Sub-Saharan Africa	0.08 (0.04 to 0.14)	0.03 (0.02 to 0.05)	-2.78 (-3.09 to -2.47)	14.17 (6.57 to 24.14)	14.52 (6.5 to 26.84)	-0.5 (-1.32 to 0.34)
Western Sub-Saharan Africa	0.03 (0.02 to 0.06)	0.03 (0.02 to 0.06)	-0.26 (-0.57 to 0.04)	5.64 (2.65 to 9.65)	9.16 (4.28 to 16.39)	1.54 (1.37 to 1.71)

DALYs, disability-adjusted life-years;
BMI, body mass index;
EAPC, estimated annual percentage change;
95% UI, 95% uncertainty interval

Supplementary Table 2. YLLs burden attribuTable to high BMI in children and adolescents at Global, SDI and geographical regional level from 1990 to 2019

Location	YLLs numbers in 1990 (95% UI)	YLLs rate in 1990 per 100000 (95% UI)	YLLs numbers in 2019 (95% UI)	YLLs rate in 2019 per 100000 (95% UI)	YLLs numbers change, 1990-2019 (%)	EAPC of YLLs rate, 1990-2019 (95% CI)
**Global**	64751 (30098 to 104961)	2.85 (1.32 to 4.62)	41767 (22471 to 66059)	1.62 (0.87 to 2.56)	-0.35 (-0.51 to -0.01)	-1.7 (-1.83 to -1.57)
**SDI**						
High SDI	3258 (1872 to 4666)	1.39 (0.8 to 1.99)	2149 (1257 to 3100)	0.97 (0.57 to 1.4)	-0.34 (-0.41 to -0.24)	-1.62 (-1.78 to -1.45)
High-middle SDI	4329 (2282 to 6806)	1.07 (0.56 to 1.68)	1585 (891 to 2436)	0.48 (0.27 to 0.74)	-0.63 (-0.73 to -0.48)	-2.97 (-3.12 to -2.82)
Middle SDI	22442 (10833 to 36112)	2.93 (1.41 to 4.71)	10113 (5537 to 15514)	1.37 (0.75 to 2.11)	-0.55 (-0.67 to -0.33)	-2.12 (-2.37 to -1.86)
Low-middle SDI	16806 (7342 to 28492)	2.95 (1.29 to 4.99)	9960 (5357 to 15784)	1.43 (0.77 to 2.27)	-0.41 (-0.57 to -0.05)	-2.35 (-2.45 to -2.24)
Low SDI	17819 (6966 to 32083)	6.03 (2.36 to 10.86)	17886 (8723 to 31018)	3 (1.46 to 5.2)	0 (-0.33 to 0.88)	-2.19 (-2.29 to -2.08)
**Regions**						
Central Asia	302 (158 to 474)	0.96 (0.5 to 1.5)	119 (65 to 193)	0.35 (0.19 to 0.57)	-0.61 (-0.71 to -0.35)	-4.02 (-4.29 to -3.75)
Central Europe	179 (96 to 272)	0.46 (0.25 to 0.71)	28 (16 to 43)	0.12 (0.07 to 0.18)	-0.84 (-0.88 to -0.79)	-5.13 (-5.33 to -4.93)
Eastern Europe	364 (189 to 541)	0.54 (0.28 to 0.8)	71 (39 to 108)	0.15 (0.08 to 0.23)	-0.81 (-0.85 to -0.71)	-5.66 (-6.04 to -5.28)
Australasia	172 (94 to 257)	2.74 (1.5 to 4.1)	85 (46 to 134)	1.19 (0.64 to 1.86)	-0.5 (-0.62 to -0.34)	-3.19 (-3.53 to -2.85)

High-income Asia Pacific	549 (297 to 809)	1.09 (0.59 to 1.61)	32 (16 to 55)	0.1 (0.05 to 0.17)	-0.94 (-0.96 to -0.91)	-9.53 (-9.99 to -9.06)
High-income North America	1316 (767 to 1893)	1.62 (0.94 to 2.32)	1557 (916 to 2248)	1.73 (1.02 to 2.5)	0.18 (0.02 to 0.37)	-0.26 (-0.5 to -0.01)
Southern Latin America	102 (53 to 160)	0.53 (0.27 to 0.83)	63 (33 to 103)	0.32 (0.17 to 0.52)	-0.38 (-0.56 to -0.15)	-1.82 (-1.9 to -1.73)
Western Europe	1014 (564 to 1471)	1.03 (0.57 to 1.5)	336 (188 to 493)	0.36 (0.2 to 0.53)	-0.67 (-0.71 to -0.59)	-3.96 (-4.14 to -3.78)
Andean Latin America	4059 (1596 to 7589)	21.21 (8.34 to 39.66)	370 (173 to 697)	1.56 (0.73 to 2.94)	-0.91 (-0.95 to -0.78)	-9 (-9.49 to -8.51)
Caribbean	1640 (639 to 3145)	10.87 (4.23 to 20.85)	994 (412 to 1940)	6.4 (2.65 to 12.49)	-0.39 (-0.6 to -0.05)	-1.44 (-1.83 to -1.06)
Central Latin America	4762 (2201 to 7416)	5.79 (2.67 to 9.01)	1052 (540 to 1802)	1.2 (0.62 to 2.06)	-0.78 (-0.85 to -0.62)	-5.74 (-6.03 to -5.46)
Tropical Latin America	2188 (965 to 3860)	3.14 (1.39 to 5.54)	879 (463 to 1461)	1.32 (0.69 to 2.19)	-0.6 (-0.74 to -0.31)	-2.68 (-2.9 to -2.47)
North Africa and Middle East	11613 (5401 to 19521)	6.44 (3 to 10.83)	4396 (2291 to 7514)	1.92 (1 to 3.28)	-0.62 (-0.75 to -0.33)	-3.62 (-3.86 to -3.37)
South Asia	7048 (2864 to 12655)	1.29 (0.52 to 2.31)	3847 (2007 to 6201)	0.55 (0.29 to 0.89)	-0.45 (-0.64 to -0.02)	-3.08 (-3.18 to -2.97)
East Asia	1543 (637 to 2585)	0.33 (0.14 to 0.56)	251 (131 to 419)	0.08 (0.04 to 0.13)	-0.84 (-0.89 to -0.67)	-5.52 (-5.98 to -5.05)
Oceania	253 (113 to 466)	7.63 (3.4 to 14.07)	396 (170 to 752)	6.48 (2.78 to 12.3)	0.57 (0 to 1.34)	-0.8 (-1.16 to -0.44)
Southeast Asia	7881 (3426 to 13441)	3.56 (1.55 to 6.07)	7143 (3791 to 11027)	3.17 (1.68 to 4.89)	-0.09 (-0.36 to 0.53)	0.04 (-0.12 to 0.2)
Central Sub-Saharan Africa	2953 (826 to 6403)	9.37 (2.62 to 20.31)	2647 (1147 to 5169)	3.72 (1.61 to 7.26)	-0.1 (-0.5 to 1.13)	-3.05 (-3.64 to -2.45)
Eastern Sub-Saharan Africa	11893 (4575 to 21624)	10.75 (4.14 to 19.55)	10083 (4661 to 18685)	4.51 (2.09 to 8.36)	-0.15 (-0.47 to 0.87)	-2.64 (-2.91 to -2.36)

Southern Sub-Saharan Africa	1792 (751 to 3143)	6.84 (2.87 to 11.99)	804 (415 to 1331)	2.63 (1.36 to 4.35)	-0.55 (-0.69 to -0.28)	-2.86 (-3.15 to -2.57)
Western Sub-Saharan Africa	3129 (1409 to 5435)	2.92 (1.31 to 5.06)	6612 (3062 to 12034)	2.66 (1.23 to 4.85)	1.11 (0.48 to 2.08)	-0.31 (-0.62 to -0.01)

YLLs, years of life lost prematurely;
BMI, body mass index;
SDI, sociodemographic index;
EAPC, estimated annual percentage change;
95% UI, 95% uncertainty interval;
95% CI, certain interval.

Supplementary Table 3. YLDs burden attribuTable to high BMI in children and adolescents at Global, SDI and geographical regional level from 1990 to 2019

Location	YLDs numbers in 1990 (95% UI)	YLDs rate in 1990 per 100000 (95% UI)	YLDs numbers in 2019 (95% UI)	YLDs rate in 2019 per 100000 (95% UI)	YLDs numbers change, 1990-2019 (%)	EAPC of YLDs rate, 1990-2019 (95% CI)
**Global**	117256 (51352 to 222293)	5.16 (2.26 to 9.78)	226723 (99180 to 426444)	8.79 (3.85 to 16.53)	0.93 (0.83 to 1.07)	1.89 (1.71 to 2.06)
**SDI**						
High SDI	37106 (16213 to 71361)	15.85 (6.92 to 30.47)	60968 (27723 to 114516)	27.61 (12.55 to 51.86)	0.64 (0.49 to 0.86)	2.16 (2.06 to 2.26)
High-middle SDI	20573 (8591 to 40041)	5.08 (2.12 to 9.88)	33218 (14305 to 64006)	10.13 (4.36 to 19.52)	0.61 (0.49 to 0.78)	2.39 (2.18 to 2.61)
Middle SDI	33764 (14355 to 64407)	4.4 (1.87 to 8.4)	66643 (28270 to 127558)	9.05 (3.84 to 17.32)	0.97 (0.79 to 1.19)	2.45 (2.15 to 2.75)
Low-middle SDI	14718 (6365 to 28049)	2.58 (1.12 to 4.92)	33066 (14027 to 62913)	4.76 (2.02 to 9.05)	1.25 (0.93 to 1.56)	2.1 (1.81 to 2.4)
Low SDI	10966 (4642 to 21558)	3.71 (1.57 to 7.3)	32625 (13993 to 62168)	5.47 (2.34 to 10.41)	1.98 (1.65 to 2.35)	1.36 (1.17 to 1.56)
**Regions**						
Central Asia	1447 (601 to 2792)	4.59 (1.9 to 8.85)	2579 (1042 to 5049)	7.59 (3.06 to 14.86)	0.78 (0.57 to 1.02)	1.71 (1.33 to 2.1)
Central Europe	2368 (985 to 4660)	6.14 (2.55 to 12.09)	2501 (1042 to 4901)	10.69 (4.45 to 20.95)	0.06 (-0.04 to 0.17)	1.88 (1.82 to 1.93)
Eastern Europe	4537 (1859 to 8939)	6.74 (2.76 to 13.28)	4203 (1746 to 8479)	8.86 (3.68 to 17.88)	-0.07 (-0.18 to 0.05)	0.56 (0.21 to 0.92)
Australasia	1918 (854 to 3509)	30.56 (13.62 to 55.92)	2255 (1022 to 4451)	31.3 (14.19 to 61.77)	0.18 (-0.08 to 0.53)	-0.52 (-0.75 to -0.3)

High-income Asia Pacific	3533 (1475 to 6928)	7.01 (2.93 to 13.75)	2929 (1222 to 5702)	9.07 (3.78 to 17.65)	-0.17 (-0.28 to -0.05)	0.28 (0.04 to 0.51)
High-income North America	21498 (9130 to 42177)	26.39 (11.21 to 51.78)	41008 (18447 to 74737)	45.59 (20.51 to 83.1)	0.91 (0.59 to 1.36)	2.48 (2.26 to 2.7)
Southern Latin America	1242 (542 to 2399)	6.41 (2.8 to 12.38)	2866 (1243 to 5726)	14.35 (6.23 to 28.67)	1.31 (0.88 to 1.89)	2.76 (2.65 to 2.87)
Western Europe	10666 (4722 to 20297)	10.84 (4.8 to 20.63)	14246 (6436 to 27483)	15.44 (6.98 to 29.79)	0.34 (0.19 to 0.49)	1.06 (0.95 to 1.18)
Andean Latin America	5345 (2261 to 10336)	27.93 (11.81 to 54.01)	6684 (2806 to 13422)	28.23 (11.85 to 56.68)	0.25 (-0.01 to 0.57)	-0.59 (-1.03 to -0.16)
Caribbean	2717 (1190 to 5266)	18.01 (7.88 to 34.9)	3328 (1410 to 6414)	21.43 (9.08 to 41.29)	0.22 (0.11 to 0.36)	0.55 (0.47 to 0.63)
Central Latin America	10430 (4447 to 20374)	12.67 (5.4 to 24.76)	14514 (6271 to 28529)	16.6 (7.17 to 32.62)	0.39 (0.19 to 0.62)	0.65 (0.39 to 0.92)
Tropical Latin America	10041 (4164 to 19449)	14.42 (5.98 to 27.93)	18548 (8057 to 35305)	27.76 (12.06 to 52.84)	0.85 (0.5 to 1.3)	2.08 (1.91 to 2.24)
North Africa and Middle East	11170 (4791 to 21240)	6.2 (2.66 to 11.78)	25737 (11065 to 49126)	11.25 (4.84 to 21.47)	1.3 (1.04 to 1.61)	2.04 (1.88 to 2.19)
South Asia	5052 (2149 to 9815)	0.92 (0.39 to 1.79)	11647 (4728 to 23122)	1.68 (0.68 to 3.33)	1.31 (0.81 to 1.91)	3.77 (2.96 to 4.58)
East Asia	6639 (2648 to 13248)	1.43 (0.57 to 2.85)	15270 (6143 to 30545)	4.91 (1.98 to 9.83)	1.3 (1.16 to 1.49)	4.16 (3.32 to 5.01)
Oceania	172 (74 to 323)	5.2 (2.25 to 9.76)	380 (163 to 723)	6.23 (2.68 to 11.84)	1.21 (0.93 to 1.53)	0.18 (-0.11 to 0.48)
Southeast Asia	4515 (1933 to 8565)	2.04 (0.87 to 3.87)	13110 (5621 to 25349)	5.82 (2.49 to 11.24)	1.9 (1.57 to 2.31)	3.74 (3.68 to 3.81)
Central Sub-Saharan Africa	1301 (542 to 2530)	4.13 (1.72 to 8.03)	4328 (1885 to 8560)	6.08 (2.65 to 12.03)	2.33 (1.75 to 3.04)	0.97 (0.73 to 1.21)
Eastern Sub-Saharan Africa	7815 (3375 to 15319)	7.06 (3.05 to 13.85)	20816 (8912 to 39904)	9.31 (3.99 to 17.85)	1.66 (1.37 to 2.01)	0.9 (0.64 to 1.16)

Southern Sub-Saharan Africa	1922 (749 to 3876)	7.33 (2.86 to 14.79)	3642 (1496 to 7172)	11.9 (4.89 to 23.43)	0.89 (0.61 to 1.26)	0.63 (-0.62 to 1.9)
Western Sub-Saharan Africa	2925 (1229 to 5601)	2.73 (1.15 to 5.22)	16132 (6758 to 30764)	6.5 (2.72 to 12.39)	4.51 (3.86 to 5.33)	2.87 (2.72 to 3.01)

YLDs, years lived with disability;
BMI, body mass index;
SDI, sociodemographic index;
EAPC, estimated annual percentage change;
95% UI, 95% uncertainty interval;
95% CI, certain interval.

Supplementary Table 4. Death burden attribuTable to high BMI in children and adolescents at national level from 1990 to 2019

Location	Deaths cases (95% UI), 1990	Deaths rate per 100000 (95% UI), 1990	Deaths cases (95% UI), 2019	Deaths rate per 100000 (95% UI), 2019	Deaths PAF per 100000 (95% UI), 2019	Deaths cases change (95% UI) (%), 1990-2019	EAPC of Deaths rate (95% CI), 1990-2019
Afghanistan	3 (1 to 5)	0.04 (0.02 to 0.08)	4 (2 to 7)	0.02 (0.01 to 0.03)	3.99 (1.68 to 7.41)	0.4 (-0.26 to 1.82)	-2.38 (-3.02 to -1.74)
Albania	0 (0 to 1)	0.02 (0.01 to 0.04)	0 (0 to 0)	0.01 (0 to 0.02)	7.81 (2.81 to 15.56)	-0.83 (-0.91 to -0.68)	-4.74 (-5.45 to -4.03)
Algeria	5 (2 to 9)	0.04 (0.02 to 0.06)	3 (1 to 5)	0.02 (0.01 to 0.03)	11.61 (5.75 to 20.63)	-0.49 (-0.69 to -0.14)	-2.22 (-2.34 to -2.09)
American Samoa	0 (0 to 0)	0.06 (0.03 to 0.1)	0 (0 to 0)	0.03 (0.01 to 0.05)	38.95 (19.98 to 66.1)	-0.48 (-0.7 to -0.16)	-2.48 (-2.77 to -2.18)
Andorra	0 (0 to 0)	0.02 (0.01 to 0.03)	0 (0 to 0)	0.01 (0 to 0.01)	31.53 (15.8 to 53.64)	-0.69 (-0.83 to -0.45)	-4.27 (-4.65 to -3.9)
Angola	7 (2 to 17)	0.12 (0.03 to 0.3)	9 (3 to 18)	0.05 (0.02 to 0.1)	12.14 (4.39 to 24.47)	0.26 (-0.45 to 2.41)	-3.06 (-3.9 to -2.21)
Antigua and Barbuda	0 (0 to 0)	0.01 (0 to 0.01)	0 (0 to 0)	0.01 (0 to 0.02)	13.51 (6.94 to 22.52)	0.38 (-0.15 to 1.24)	0.7 (0.21 to 1.18)
Argentina	1 (0 to 2)	0.01 (0 to 0.01)	1 (0 to 1)	0 (0 to 0.01)	5.12 (2.45 to 9.01)	-0.43 (-0.63 to -0.14)	-2.48 (-2.58 to -2.37)
Armenia	0 (0 to 0)	0 (0 to 0)	0 (0 to 0)	0 (0 to 0)	0.81 (0.36 to 1.91)	-0.46 (-0.69 to -0.06)	0.08 (-0.61 to 0.78)
Australia	2 (1 to 3)	0.03 (0.02 to 0.05)	1 (0 to 1)	0.01 (0.01 to 0.02)	46.33 (23.79 to 75.74)	-0.51 (-0.65 to -0.31)	-3.28 (-3.75 to -2.8)
Austria	0 (0 to 0)	0.01 (0 to 0.01)	0 (0 to 0)	0 (0 to 0)	6.86 (3.29 to 11.85)	-0.74 (-0.83 to -0.59)	-5.22 (-5.6 to -4.84)
Azerbaijan	0 (0 to 0)	0 (0 to 0.01)	0 (0 to 0)	0 (0 to 0.01)	1.78 (0.86 to 3.07)	-0.21 (-0.54 to 0.4)	-1.55 (-2.18 to -0.91)

Bahamas	0 (0 to 0)	0.02 (0.01 to 0.04)	0 (0 to 0)	0.02 (0.01 to 0.03)	19.01 (9.51 to 32.04)	-0.25 (-0.55 to 0.2)	-1.05 (-1.35 to -0.76)
Bahrain	0 (0 to 0)	0.06 (0.03 to 0.1)	0 (0 to 0)	0.02 (0.01 to 0.04)	50.65 (26.45 to 83.67)	-0.35 (-0.55 to -0.04)	-3.04 (-3.32 to -2.77)
Bangladesh	7 (3 to 13)	0.01 (0.01 to 0.02)	2 (1 to 4)	0 (0 to 0.01)	1.96 (0.88 to 3.99)	-0.7 (-0.84 to -0.38)	-4.25 (-4.5 to -4.01)
Barbados	0 (0 to 0)	0.03 (0.02 to 0.05)	0 (0 to 0)	0.02 (0.01 to 0.04)	26.75 (14.08 to 43.59)	-0.46 (-0.64 to -0.16)	-1.69 (-1.95 to -1.43)
Belarus	0 (0 to 0)	0 (0 to 0.01)	0 (0 to 0)	0 (0 to 0)	1.58 (0.65 to 4.02)	-0.88 (-0.94 to -0.72)	-6.92 (-7.24 to -6.61)
Belgium	0 (0 to 1)	0.01 (0.01 to 0.02)	0 (0 to 0)	0 (0 to 0.01)	13.28 (6.39 to 22.54)	-0.72 (-0.81 to -0.59)	-5.45 (-5.75 to -5.15)
Belize	0 (0 to 0)	0.04 (0.02 to 0.07)	0 (0 to 0)	0.03 (0.02 to 0.06)	30.48 (15.57 to 50.66)	0.3 (-0.18 to 1.12)	-1.32 (-1.76 to -0.88)
Benin	1 (0 to 3)	0.05 (0.02 to 0.09)	3 (1 to 7)	0.05 (0.02 to 0.1)	7.19 (2.83 to 14)	1.57 (0.25 to 3.97)	0.34 (-0.1 to 0.79)
Bermuda	0 (0 to 0)	0.03 (0.01 to 0.05)	0 (0 to 0)	0.01 (0.01 to 0.02)	31.28 (16.37 to 50.76)	-0.68 (-0.79 to -0.51)	-2.97 (-3.28 to -2.65)
Bhutan	0 (0 to 0)	0.02 (0 to 0.04)	0 (0 to 0)	0.01 (0 to 0.02)	5.99 (2.63 to 11)	-0.43 (-0.76 to 1.45)	-1.33 (-1.56 to -1.1)
Bolivia (Plurinational State of)	17 (5 to 37)	0.5 (0.15 to 1.09)	2 (1 to 5)	0.05 (0.02 to 0.1)	21.41 (8.4 to 40.88)	-0.86 (-0.94 to -0.65)	-8.36 (-8.71 to -8.02)
Bosnia and Herzegovina	0 (0 to 0)	0 (0 to 0)	0 (0 to 0)	0 (0 to 0)	3.15 (1.6 to 5.18)	-0.65 (-0.78 to -0.42)	-0.72 (-1.02 to -0.42)
Botswana	0 (0 to 0)	0.04 (0.01 to 0.06)	0 (0 to 1)	0.05 (0.02 to 0.11)	18.07 (5.27 to 35.72)	0.89 (-0.26 to 2.61)	2.03 (1.73 to 2.33)
Brazil	25 (11 to 45)	0.04 (0.02 to 0.07)	11 (6 to 17)	0.02 (0.01 to 0.03)	11.74 (6.37 to 19.1)	-0.59 (-0.73 to -0.29)	-2.54 (-2.76 to -2.33)

Brunei Darussalam	0 (0 to 0)	0.01 (0 to 0.02)	0 (0 to 0)	0 (0 to 0.01)	5.7 (2.66 to 9.9)	-0.5 (-0.69 to -0.13)	-2.93 (-3.26 to -2.6)
Bulgaria	0 (0 to 0)	0.01 (0 to 0.01)	0 (0 to 0)	0 (0 to 0)	2.1 (1.01 to 3.76)	-0.88 (-0.92 to -0.8)	-6.05 (-6.57 to -5.54)
Burkina Faso	3 (1 to 5)	0.05 (0.02 to 0.09)	8 (3 to 18)	0.06 (0.02 to 0.14)	7.43 (2.7 to 16.07)	2.18 (0.51 to 5.74)	1.09 (0.82 to 1.36)
Burundi	5 (2 to 12)	0.17 (0.06 to 0.37)	4 (1 to 9)	0.06 (0.02 to 0.13)	11.17 (4.39 to 24.96)	-0.28 (-0.7 to 0.96)	-3.45 (-3.79 to -3.11)
Cabo Verde	0 (0 to 0)	0.01 (0.01 to 0.03)	0 (0 to 0)	0.01 (0 to 0.01)	4.56 (1.92 to 9.03)	-0.53 (-0.78 to 0.14)	-3.31 (-3.6 to -3.02)
Cambodia	1 (0 to 3)	0.02 (0.01 to 0.05)	1 (0 to 2)	0.01 (0.01 to 0.03)	6.41 (2.97 to 11.5)	-0.31 (-0.65 to 1)	-1.52 (-1.65 to -1.39)
Cameroon	3 (1 to 6)	0.06 (0.02 to 0.11)	7 (3 to 13)	0.04 (0.02 to 0.09)	8.86 (3.76 to 16.98)	1.04 (0.07 to 2.86)	-0.99 (-1.33 to -0.65)
Canada	1 (1 to 2)	0.01 (0.01 to 0.02)	0 (0 to 1)	0.01 (0 to 0.01)	16.95 (8.57 to 28.71)	-0.57 (-0.69 to -0.39)	-3.52 (-3.78 to -3.25)
Central African Republic	2 (1 to 5)	0.16 (0.05 to 0.36)	3 (1 to 6)	0.09 (0.03 to 0.2)	9 (2.98 to 20.28)	0.03 (-0.48 to 1)	-1.87 (-2.23 to -1.52)
Chad	1 (0 to 2)	0.03 (0.01 to 0.07)	4 (1 to 8)	0.04 (0.01 to 0.08)	4.03 (1.55 to 8.28)	2.45 (0.73 to 5.86)	0.02 (-0.52 to 0.57)
Chile	0 (0 to 0)	0 (0 to 0.01)	0 (0 to 0)	0 (0 to 0.01)	6.71 (3.17 to 11.55)	-0.15 (-0.44 to 0.29)	0.3 (0.03 to 0.58)
China	18 (7 to 29)	0 (0 to 0.01)	3 (2 to 5)	0 (0 to 0)	1.55 (0.81 to 2.67)	-0.83 (-0.89 to -0.65)	-5.38 (-5.83 to -4.93)
Colombia	4 (2 to 6)	0.02 (0.01 to 0.04)	1 (0 to 2)	0.01 (0 to 0.01)	5.28 (2.25 to 11.72)	-0.77 (-0.87 to -0.55)	-5.22 (-5.74 to -4.69)
Comoros	0 (0 to 1)	0.17 (0.04 to 0.38)	0 (0 to 0)	0.06 (0.02 to 0.13)	18.97 (7.28 to 37.99)	-0.58 (-0.8 to 0.53)	-3.7 (-4.14 to -3.25)

Congo	1 (0 to 2)	0.07 (0.03 to 0.14)	1 (0 to 1)	0.03 (0.01 to 0.05)	9.32 (4.11 to 17.7)	-0.26 (-0.62 to 0.48)	-3.42 (-3.93 to -2.9)
Cook Islands	0 (0 to 0)	0.04 (0.02 to 0.07)	0 (0 to 0)	0.01 (0 to 0.02)	28.43 (10.53 to 55.35)	-0.86 (-0.94 to -0.71)	-5.89 (-6.41 to -5.38)
Costa Rica	0 (0 to 1)	0.02 (0.01 to 0.04)	0 (0 to 0)	0.01 (0.01 to 0.02)	18.54 (9.19 to 34.35)	-0.44 (-0.66 to -0.08)	-2.56 (-3.07 to -2.04)
C 么 te d'Ivoire	2 (1 to 4)	0.03 (0.01 to 0.05)	5 (2 to 10)	0.04 (0.01 to 0.07)	6.36 (2.64 to 12.41)	1.5 (0.27 to 3.66)	0.95 (0.4 to 1.5)
Croatia	0 (0 to 0)	0 (0 to 0)	0 (0 to 0)	0 (0 to 0)	2.99 (1.44 to 5.02)	-0.81 (-0.88 to -0.71)	-5.05 (-5.35 to -4.74)
Cuba	1 (0 to 2)	0.03 (0.01 to 0.04)	0 (0 to 1)	0.02 (0.01 to 0.03)	41.79 (21.18 to 71.58)	-0.57 (-0.71 to -0.35)	-1.69 (-1.96 to -1.42)
Cyprus	0 (0 to 0)	0 (0 to 0)	0 (0 to 0)	0 (0 to 0)	3.24 (1.53 to 6.53)	-0.56 (-0.75 to -0.22)	-4.41 (-5.27 to -3.54)
Czechia	0 (0 to 0)	0.01 (0 to 0.01)	0 (0 to 0)	0 (0 to 0)	9.84 (4.67 to 16.57)	-0.74 (-0.84 to -0.6)	-3.63 (-3.81 to -3.45)
Democratic People's Republic of Korea	1 (0 to 1)	0.01 (0 to 0.01)	0 (0 to 0)	0 (0 to 0)	1.69 (0.73 to 3.05)	-0.79 (-0.9 to -0.5)	-4.08 (-4.39 to -3.77)
Democratic Republic of the Congo	24 (6 to 58)	0.11 (0.03 to 0.26)	20 (8 to 42)	0.04 (0.02 to 0.09)	9.78 (4.02 to 19.51)	-0.18 (-0.63 to 1.17)	-3.1 (-3.62 to -2.58)
Denmark	0 (0 to 0)	0.01 (0 to 0.02)	0 (0 to 0)	0 (0 to 0.01)	11.46 (5.23 to 20.71)	-0.68 (-0.8 to -0.49)	-5.49 (-6.07 to -4.92)
Djibouti	0 (0 to 0)	0.07 (0.03 to 0.15)	0 (0 to 1)	0.06 (0.02 to 0.13)	15.92 (6.19 to 32.3)	0.64 (-0.15 to 2.35)	-0.63 (-1.2 to -0.05)
Dominica	0 (0 to 0)	0.04 (0.02 to 0.06)	0 (0 to 0)	0.07 (0.03 to 0.12)	46.56 (24.09 to 76.64)	0.15 (-0.32 to 0.82)	2.34 (1.88 to 2.81)
Dominican Republic	6 (2 to 11)	0.16 (0.07 to 0.3)	3 (1 to 5)	0.07 (0.03 to 0.12)	35.96 (16.89 to 64.11)	-0.55 (-0.75 to -0.21)	-2.72 (-3.25 to -2.19)

Ecuador	11 (4 to 21)	0.22 (0.08 to 0.41)	1 (0 to 2)	0.01 (0 to 0.02)	9.24 (4.34 to 19.71)	-0.93 (-0.96 to -0.7)	-9.64 (-10.74 to -8.54)
Egypt	69 (29 to 128)	0.25 (0.1 to 0.46)	20 (9 to 40)	0.05 (0.02 to 0.09)	41.87 (19.95 to 85.87)	-0.72 (-0.86 to -0.36)	-5.23 (-5.63 to -4.84)
El Salvador	3 (1 to 6)	0.12 (0.04 to 0.23)	0 (0 to 0)	0.01 (0 to 0.02)	8.63 (3.69 to 16.15)	-0.94 (-0.97 to -0.83)	-8.58 (-9.49 to -7.65)
Equatorial Guinea	0 (0 to 1)	0.12 (0.03 to 0.29)	0 (0 to 1)	0.04 (0.02 to 0.09)	15.06 (6.25 to 30.76)	-0.02 (-0.61 to 1.9)	-4.41 (-5.07 to -3.74)
Eritrea	2 (1 to 4)	0.1 (0.03 to 0.22)	2 (1 to 4)	0.05 (0.02 to 0.1)	14.32 (6.03 to 27.99)	-0.02 (-0.57 to 1.3)	-2.05 (-2.34 to -1.77)
Estonia	0 (0 to 0)	0 (0 to 0.01)	0 (0 to 0)	0 (0 to 0)	4.74 (2.18 to 8.54)	-0.82 (-0.9 to -0.7)	-4.41 (-4.87 to -3.96)
Eswatini	1 (0 to 1)	0.12 (0.05 to 0.23)	0 (0 to 1)	0.07 (0.03 to 0.13)	19.34 (9.43 to 33)	-0.31 (-0.59 to 0.17)	-1.32 (-1.57 to -1.07)
Ethiopia	18 (6 to 38)	0.06 (0.02 to 0.13)	15 (7 to 29)	0.03 (0.01 to 0.05)	6.71 (3.06 to 12.69)	-0.18 (-0.6 to 1.33)	-3 (-3.24 to -2.77)
Fiji	0 (0 to 1)	0.12 (0.06 to 0.21)	0 (0 to 1)	0.13 (0.06 to 0.21)	79.37 (42.17 to 125.3)	-0.03 (-0.41 to 0.52)	-0.93 (-1.62 to -0.23)
Finland	0 (0 to 0)	0.01 (0 to 0.01)	0 (0 to 0)	0 (0 to 0)	9.43 (4.73 to 15.8)	-0.65 (-0.76 to -0.48)	-3.27 (-3.58 to -2.96)
France	2 (1 to 3)	0.01 (0.01 to 0.02)	1 (0 to 1)	0 (0 to 0.01)	12.19 (5.97 to 20.45)	-0.7 (-0.8 to -0.53)	-5.17 (-5.65 to -4.7)
Gabon	0 (0 to 0)	0.04 (0.01 to 0.1)	0 (0 to 0)	0.02 (0.01 to 0.04)	10.87 (4.57 to 20.19)	-0.21 (-0.57 to 0.54)	-1.98 (-2.29 to -1.67)
Gambia	0 (0 to 0)	0.03 (0.01 to 0.05)	0 (0 to 0)	0.02 (0.01 to 0.04)	7.03 (2.8 to 14.24)	0.52 (-0.26 to 2.55)	-1.61 (-2.12 to -1.1)
Georgia	1 (0 to 1)	0.04 (0.02 to 0.06)	0 (0 to 1)	0.03 (0.01 to 0.06)	43.19 (19.12 to 73.35)	-0.56 (-0.71 to -0.34)	-0.94 (-1.31 to -0.57)
Germany	3 (2 to 5)	0.02 (0.01 to 0.03)	0 (0 to 1)	0 (0 to 0.01)	11.98 (5.79 to 20.62)	-0.83 (-0.89 to -0.71)	-6.54 (-6.91 to -6.18)

Ghana	2 (1 to 4)	0.03 (0.01 to 0.05)	4 (2 to 8)	0.03 (0.01 to 0.05)	7.51 (3.06 to 14)	0.9 (0.03 to 2.49)	0.14 (-0.29 to 0.58)
Greece	0 (0 to 0)	0 (0 to 0.01)	0 (0 to 0)	0 (0 to 0)	6.04 (2.87 to 10.59)	-0.7 (-0.79 to -0.57)	-2.93 (-3.26 to -2.6)
Greenland	0 (0 to 0)	0.04 (0.02 to 0.06)	0 (0 to 0)	0.01 (0.01 to 0.03)	18.35 (8.92 to 34.64)	-0.67 (-0.8 to -0.41)	-4.44 (-5.13 to -3.74)
Grenada	0 (0 to 0)	0.02 (0.01 to 0.03)	0 (0 to 0)	0.01 (0.01 to 0.03)	16.06 (7.74 to 27.16)	-0.36 (-0.61 to 0.06)	-0.29 (-0.68 to 0.11)
Guam	0 (0 to 0)	0.02 (0.01 to 0.04)	0 (0 to 0)	0.01 (0.01 to 0.02)	13.92 (7.11 to 23.37)	-0.25 (-0.53 to 0.17)	-1.53 (-2.36 to -0.68)
Guatemala	13 (5 to 23)	0.3 (0.11 to 0.51)	2 (1 to 4)	0.02 (0.01 to 0.05)	14.78 (7.17 to 25.59)	-0.86 (-0.92 to -0.7)	-8.97 (-10.43 to -7.48)
Guinea	3 (1 to 6)	0.08 (0.03 to 0.17)	4 (2 to 8)	0.06 (0.02 to 0.12)	7.79 (3.19 to 15.79)	0.45 (-0.27 to 1.94)	-1.3 (-1.48 to -1.11)
Guinea-Bissau	0 (0 to 1)	0.06 (0.02 to 0.13)	0 (0 to 1)	0.03 (0.01 to 0.07)	6.14 (2.57 to 12.07)	-0.08 (-0.57 to 1.04)	-2.29 (-3.09 to -1.49)
Guyana	0 (0 to 0)	0.04 (0.02 to 0.06)	0 (0 to 0)	0.04 (0.02 to 0.07)	23.74 (12.5 to 39.08)	-0.18 (-0.47 to 0.3)	1.39 (0.86 to 1.92)
Haiti	10 (2 to 23)	0.3 (0.07 to 0.7)	8 (2 to 17)	0.14 (0.04 to 0.3)	27.95 (8.46 to 57.71)	-0.23 (-0.59 to 0.5)	-2.01 (-2.43 to -1.58)
Honduras	4 (2 to 9)	0.16 (0.06 to 0.33)	2 (1 to 3)	0.04 (0.01 to 0.08)	29.33 (11.49 to 59.26)	-0.64 (-0.83 to -0.24)	-5.37 (-5.56 to -5.18)
Hungary	0 (0 to 0)	0.01 (0 to 0.01)	0 (0 to 0)	0 (0 to 0)	7.14 (3.44 to 11.91)	-0.79 (-0.86 to -0.66)	-3.8 (-4.07 to -3.53)
Iceland	0 (0 to 0)	0.01 (0.01 to 0.02)	0 (0 to 0)	0 (0 to 0.01)	17.75 (8.46 to 31.15)	-0.66 (-0.79 to -0.46)	-4.13 (-4.53 to -3.72)
India	62 (25 to 114)	0.01 (0.01 to 0.03)	34 (17 to 58)	0.01 (0 to 0.01)	3.13 (1.55 to 5.22)	-0.45 (-0.65 to 0.07)	-3.02 (-3.15 to -2.88)
Indonesia	47 (19 to 87)	0.05 (0.02 to 0.1)	36 (19 to 59)	0.04 (0.02 to 0.07)	26.91 (13.79 to 42.31)	-0.23 (-0.52 to 0.42)	-0.61 (-0.74 to -0.47)

Iran (Islamic Republic of)	10 (5 to 19)	0.03 (0.01 to 0.06)	3 (2 to 6)	0.01 (0.01 to 0.02)	13.96 (6.99 to 24.96)	-0.67 (-0.8 to -0.42)	-2.3 (-2.97 to -1.63)
Iraq	4 (2 to 8)	0.04 (0.02 to 0.08)	2 (1 to 4)	0.01 (0.01 to 0.02)	9.59 (4.7 to 16.8)	-0.53 (-0.72 to -0.15)	-4.29 (-4.49 to -4.09)
Ireland	0 (0 to 0)	0.02 (0.01 to 0.04)	0 (0 to 0)	0.01 (0 to 0.01)	27.66 (13.2 to 48.88)	-0.69 (-0.8 to -0.51)	-5.13 (-5.81 to -4.45)
Israel	0 (0 to 1)	0.02 (0.01 to 0.03)	0 (0 to 0)	0 (0 to 0.01)	16.09 (7.75 to 27.98)	-0.62 (-0.77 to -0.34)	-5.52 (-5.89 to -5.16)
Italy	1 (1 to 2)	0.01 (0 to 0.01)	0 (0 to 0)	0 (0 to 0)	5.87 (3.01 to 10.21)	-0.87 (-0.91 to -0.81)	-7.34 (-7.78 to -6.9)
Jamaica	1 (0 to 1)	0.07 (0.03 to 0.12)	0 (0 to 0)	0.03 (0.01 to 0.05)	33.99 (17.65 to 56.11)	-0.65 (-0.78 to -0.39)	-3.42 (-3.76 to -3.07)
Japan	5 (2 to 7)	0.01 (0.01 to 0.02)	0 (0 to 0)	0 (0 to 0)	6.33 (3.05 to 10.3)	-0.94 (-0.96 to -0.92)	-9.76 (-10.23 to -9.3)
Jordan	0 (0 to 1)	0.02 (0.01 to 0.03)	1 (0 to 1)	0.01 (0.01 to 0.02)	11.46 (5.62 to 19.71)	0.32 (-0.15 to 0.98)	-2.62 (-3.09 to -2.15)
Kazakhstan	1 (0 to 1)	0.01 (0 to 0.02)	0 (0 to 1)	0.01 (0 to 0.01)	5.87 (2.84 to 10.11)	-0.45 (-0.65 to -0.12)	-2.99 (-3.4 to -2.59)
Kenya	6 (3 to 12)	0.05 (0.02 to 0.09)	8 (3 to 14)	0.03 (0.01 to 0.06)	11.02 (5.09 to 20.74)	0.21 (-0.27 to 1.34)	-0.4 (-1.07 to 0.28)
Kiribati	0 (0 to 0)	0.16 (0.08 to 0.29)	0 (0 to 0)	0.11 (0.05 to 0.2)	40.97 (18.65 to 74.28)	-0.05 (-0.43 to 0.56)	-1.71 (-2.06 to -1.37)
Kuwait	0 (0 to 1)	0.05 (0.03 to 0.08)	0 (0 to 0)	0.02 (0.01 to 0.03)	24.08 (12.64 to 39.21)	-0.49 (-0.68 to -0.18)	-3.02 (-3.63 to -2.4)
Kyrgyzstan	0 (0 to 0)	0 (0 to 0.01)	0 (0 to 0)	0 (0 to 0)	1.02 (0.45 to 2.85)	-0.65 (-0.81 to -0.22)	-5.19 (-5.58 to -4.8)
Lao People's Democratic Republic	2 (1 to 5)	0.1 (0.03 to 0.21)	2 (1 to 3)	0.05 (0.02 to 0.1)	18.28 (8.41 to 32.13)	-0.32 (-0.67 to 0.88)	-2.17 (-2.29 to -2.06)

Latvia	0 (0 to 0)	0.01 (0 to 0.01)	0 (0 to 0)	0 (0 to 0)	2.91 (1.34 to 5.29)	-0.92 (-0.95 to -0.85)	-6.98 (-7.34 to -6.61)
Lebanon	0 (0 to 1)	0.02 (0.01 to 0.03)	0 (0 to 0)	0.01 (0 to 0.02)	10.98 (5.1 to 20.38)	-0.47 (-0.69 to -0.12)	-2.84 (-3.05 to -2.63)
Lesotho	1 (0 to 1)	0.05 (0.02 to 0.1)	1 (0 to 1)	0.07 (0.03 to 0.14)	14.68 (6.43 to 26.83)	0.24 (-0.28 to 1.07)	2.06 (1.54 to 2.58)
Liberia	1 (0 to 3)	0.11 (0.03 to 0.23)	1 (0 to 1)	0.02 (0.01 to 0.06)	5.01 (1.68 to 13.55)	-0.59 (-0.84 to 0.15)	-4.87 (-5.61 to -4.12)
Libya	1 (0 to 2)	0.04 (0.02 to 0.07)	0 (0 to 1)	0.02 (0.01 to 0.03)	19.3 (9.35 to 33.16)	-0.6 (-0.78 to -0.31)	-2.55 (-2.76 to -2.33)
Lithuania	0 (0 to 0)	0 (0 to 0.01)	0 (0 to 0)	0 (0 to 0)	2.46 (1.16 to 4.45)	-0.87 (-0.92 to -0.75)	-4.6 (-4.96 to -4.25)
Luxembourg	0 (0 to 0)	0.02 (0.01 to 0.03)	0 (0 to 0)	0 (0 to 0.01)	17.92 (8.61 to 31.23)	-0.62 (-0.76 to -0.39)	-5.09 (-5.56 to -4.61)
Madagascar	15 (7 to 26)	0.21 (0.1 to 0.38)	14 (5 to 34)	0.1 (0.04 to 0.24)	23.99 (9.57 to 59.44)	-0.05 (-0.51 to 1.21)	-2.35 (-2.61 to -2.09)
Malawi	13 (3 to 28)	0.23 (0.06 to 0.51)	7 (3 to 13)	0.06 (0.03 to 0.12)	16.55 (7.5 to 30.82)	-0.48 (-0.75 to 0.69)	-4.48 (-4.74 to -4.21)
Malaysia	2 (1 to 4)	0.03 (0.01 to 0.05)	1 (1 to 2)	0.01 (0.01 to 0.02)	20.91 (10.58 to 35.19)	-0.46 (-0.68 to -0.1)	-3.54 (-4.05 to -3.02)
Maldives	0 (0 to 0)	0.04 (0.01 to 0.09)	0 (0 to 0)	0.01 (0.01 to 0.02)	10.26 (4.44 to 19.39)	-0.68 (-0.85 to -0.26)	-4.1 (-4.76 to -3.45)
Mali	1 (0 to 2)	0.01 (0 to 0.04)	2 (1 to 6)	0.02 (0.01 to 0.05)	1.77 (0.64 to 5.48)	3.07 (1.29 to 6.86)	1.39 (1.2 to 1.57)
Malta	0 (0 to 0)	0.01 (0 to 0.01)	0 (0 to 0)	0 (0 to 0.01)	7.51 (3.5 to 13.15)	-0.59 (-0.74 to -0.37)	-2.7 (-3.17 to -2.23)
Marshall Islands	0 (0 to 0)	0.04 (0.02 to 0.07)	0 (0 to 0)	0.05 (0.02 to 0.08)	30.6 (14.32 to 52.94)	0.09 (-0.29 to 0.71)	0.29 (-0.38 to 0.96)
Mauritania	0 (0 to 1)	0.03 (0.01 to 0.06)	0 (0 to 1)	0.02 (0.01 to 0.03)	5.54 (2.43 to 10.06)	-0.07 (-0.53 to 0.86)	-2.84 (-3.21 to -2.46)

Mauritius	0 (0 to 0)	0.07 (0.03 to 0.11)	0 (0 to 0)	0.05 (0.02 to 0.09)	65.55 (32.87 to 110.28)	-0.47 (-0.66 to -0.16)	-0.83 (-1.01 to -0.66)
Mexico	23 (11 to 39)	0.05 (0.03 to 0.09)	6 (3 to 10)	0.01 (0.01 to 0.02)	12.59 (6.95 to 21.19)	-0.76 (-0.84 to -0.61)	-5.12 (-5.31 to -4.93)
Micronesia (Federated States of)	0 (0 to 0)	0.07 (0.03 to 0.12)	0 (0 to 0)	0.04 (0.02 to 0.08)	36.29 (14.9 to 64.45)	-0.57 (-0.78 to -0.32)	-2.11 (-2.27 to -1.95)
Monaco	0 (0 to 0)	0.01 (0 to 0.01)	0 (0 to 0)	0 (0 to 0.01)	18.13 (8.49 to 31.83)	-0.23 (-0.51 to 0.24)	-1.82 (-1.88 to -1.76)
Mongolia	0 (0 to 1)	0.02 (0 to 0.07)	0 (0 to 0)	0 (0 to 0.01)	2.72 (1.07 to 5.98)	-0.81 (-0.91 to -0.38)	-6.49 (-7.13 to -5.85)
Montenegro	0 (0 to 0)	0 (0 to 0)	0 (0 to 0)	0 (0 to 0)	2.69 (1.37 to 4.45)	-0.65 (-0.78 to -0.49)	-2.51 (-2.96 to -2.05)
Morocco	6 (2 to 12)	0.05 (0.02 to 0.09)	3 (1 to 5)	0.02 (0.01 to 0.04)	17.91 (8.28 to 32.81)	-0.55 (-0.75 to -0.07)	-2.55 (-2.72 to -2.37)
Mozambique	10 (3 to 25)	0.14 (0.04 to 0.33)	12 (5 to 24)	0.07 (0.03 to 0.14)	12.12 (4.96 to 24.18)	0.15 (-0.55 to 3.73)	-2.49 (-2.77 to -2.21)
Myanmar	12 (5 to 25)	0.06 (0.02 to 0.12)	14 (6 to 27)	0.07 (0.03 to 0.14)	26.25 (11.99 to 46.59)	0.16 (-0.46 to 1.88)	0.93 (0.68 to 1.18)
Namibia	0 (0 to 1)	0.04 (0.02 to 0.09)	0 (0 to 1)	0.03 (0.01 to 0.05)	9.26 (3.94 to 16.45)	-0.17 (-0.59 to 0.78)	-1.06 (-1.4 to -0.71)
Nauru	0 (0 to 0)	0.15 (0.06 to 0.3)	0 (0 to 0)	0.08 (0.03 to 0.15)	39.23 (17.36 to 70.87)	-0.53 (-0.71 to -0.22)	-2.79 (-3.42 to -2.15)
Nepal	1 (0 to 2)	0.01 (0 to 0.02)	1 (0 to 1)	0 (0 to 0.01)	2.39 (0.94 to 6.2)	-0.36 (-0.76 to 1.4)	-1.63 (-2.1 to -1.16)
Netherlands	0 (0 to 0)	0.01 (0 to 0.01)	0 (0 to 0)	0 (0 to 0)	7.92 (3.77 to 13.9)	-0.66 (-0.78 to -0.5)	-3.71 (-4.14 to -3.29)
New Zealand	1 (0 to 1)	0.05 (0.03 to 0.08)	0 (0 to 0)	0.03 (0.01 to 0.04)	64.91 (35.55 to 102.73)	-0.48 (-0.61 to -0.31)	-2.85 (-3.07 to -2.64)

Nicaragua	2 (1 to 4)	0.09 (0.04 to 0.18)	1 (0 to 1)	0.02 (0.01 to 0.05)	21.98 (8.67 to 46.63)	-0.73 (-0.88 to -0.41)	-5.24 (-5.43 to -5.05)
Niger	3 (1 to 7)	0.06 (0.02 to 0.14)	6 (2 to 14)	0.04 (0.02 to 0.09)	4.64 (1.67 to 9.75)	0.98 (-0.09 to 3.32)	-1.81 (-2.23 to -1.4)
Nigeria	13 (5 to 24)	0.03 (0.01 to 0.05)	31 (13 to 59)	0.03 (0.01 to 0.05)	3.63 (1.56 to 6.46)	1.46 (0.52 to 3.16)	0.21 (-0.17 to 0.59)
Niue	0 (0 to 0)	0.06 (0.03 to 0.12)	0 (0 to 0)	0.05 (0.02 to 0.1)	40.36 (17.4 to 75.1)	-0.56 (-0.76 to -0.26)	-0.93 (-1.37 to -0.5)
North Macedonia	0 (0 to 0)	0 (0 to 0.01)	0 (0 to 0)	0 (0 to 0)	2.41 (1.18 to 4.18)	-0.78 (-0.87 to -0.62)	-4.12 (-4.39 to -3.85)
Northern Mariana Islands	0 (0 to 0)	0.03 (0.01 to 0.05)	0 (0 to 0)	0.02 (0.01 to 0.03)	20.89 (10.26 to 36.79)	-0.62 (-0.75 to -0.42)	-1.7 (-2 to -1.4)
Norway	0 (0 to 0)	0.01 (0 to 0.01)	0 (0 to 0)	0 (0 to 0)	11.07 (5.93 to 17.34)	-0.65 (-0.74 to -0.54)	-4.28 (-4.52 to -4.03)
Oman	0 (0 to 0)	0 (0 to 0.01)	0 (0 to 0)	0 (0 to 0)	3.02 (1.43 to 5.19)	0.28 (-0.23 to 1.16)	0.68 (0.31 to 1.05)
Pakistan	15 (5 to 32)	0.02 (0.01 to 0.05)	12 (6 to 21)	0.01 (0.01 to 0.02)	2.42 (1.16 to 4.1)	-0.17 (-0.55 to 0.77)	-2.57 (-2.78 to -2.37)
Palau	0 (0 to 0)	0.05 (0.02 to 0.11)	0 (0 to 0)	0.02 (0.01 to 0.05)	23.65 (10.74 to 44.01)	-0.66 (-0.83 to -0.24)	-2.07 (-2.26 to -1.88)
Palestine	0 (0 to 0)	0.01 (0.01 to 0.03)	0 (0 to 0)	0 (0 to 0.01)	5.46 (2.53 to 10.78)	-0.27 (-0.61 to 0.43)	-2.88 (-3.28 to -2.49)
Panama	1 (0 to 1)	0.06 (0.02 to 0.1)	0 (0 to 1)	0.03 (0.01 to 0.05)	25.47 (12.13 to 42.96)	-0.37 (-0.63 to 0.14)	-2.18 (-2.64 to -1.71)
Papua New Guinea	2 (1 to 4)	0.1 (0.04 to 0.2)	4 (2 to 8)	0.08 (0.03 to 0.17)	19.12 (7.99 to 37)	0.81 (0.07 to 1.98)	-0.8 (-1.14 to -0.46)
Paraguay	0 (0 to 1)	0.02 (0.01 to 0.04)	0 (0 to 0)	0.01 (0 to 0.01)	7.71 (3.95 to 12.87)	-0.57 (-0.77 to -0.2)	-2.95 (-3.4 to -2.51)

Peru	19 (7 to 40)	0.18 (0.07 to 0.38)	1 (1 to 3)	0.01 (0 to 0.02)	9.58 (4.06 to 19.64)	-0.93 (-0.97 to -0.83)	-9.69 (-10.28 to -9.08)
Philippines	18 (8 to 31)	0.06 (0.02 to 0.1)	27 (14 to 45)	0.06 (0.03 to 0.1)	33.72 (16.88 to 53.14)	0.52 (0.03 to 1.51)	1.11 (0.7 to 1.53)
Poland	1 (0 to 1)	0.01 (0 to 0.01)	0 (0 to 0)	0 (0 to 0)	3.78 (1.9 to 6.01)	-0.86 (-0.9 to -0.8)	-5.58 (-5.78 to -5.39)
Portugal	0 (0 to 1)	0.01 (0 to 0.02)	0 (0 to 0)	0 (0 to 0)	5.57 (2.49 to 10.48)	-0.92 (-0.95 to -0.86)	-8.59 (-9.18 to -8)
Puerto Rico	1 (0 to 1)	0.04 (0.02 to 0.07)	0 (0 to 0)	0.02 (0.01 to 0.03)	43.36 (22 to 72.3)	-0.74 (-0.84 to -0.6)	-4.1 (-4.6 to -3.59)
Qatar	0 (0 to 0)	0.05 (0.03 to 0.09)	0 (0 to 0)	0.02 (0.01 to 0.03)	24.62 (12.64 to 40.76)	0.07 (-0.34 to 0.82)	-3.96 (-4.23 to -3.7)
Republic of Korea	2 (1 to 3)	0.01 (0.01 to 0.02)	0 (0 to 0)	0 (0 to 0)	5.33 (2.09 to 10.33)	-0.95 (-0.97 to -0.9)	-9.62 (-10.24 to -8.99)
Republic of Moldova	0 (0 to 0)	0 (0 to 0)	0 (0 to 0)	0 (0 to 0)	0.72 (0.31 to 1.57)	-0.86 (-0.92 to -0.75)	-5.06 (-5.51 to -4.62)
Romania	0 (0 to 1)	0 (0 to 0.01)	0 (0 to 0)	0 (0 to 0)	1.29 (0.54 to 2.86)	-0.89 (-0.94 to -0.78)	-5.53 (-5.87 to -5.18)
Russian Federation	4 (2 to 6)	0.01 (0 to 0.01)	1 (0 to 1)	0 (0 to 0)	4.03 (2.17 to 6.23)	-0.81 (-0.85 to -0.72)	-5.95 (-6.39 to -5.51)
Rwanda	12 (4 to 25)	0.29 (0.1 to 0.6)	6 (2 to 13)	0.09 (0.03 to 0.2)	28.58 (10.89 to 63.11)	-0.53 (-0.78 to 0.35)	-3.96 (-4.41 to -3.52)
Saint Kitts and Nevis	0 (0 to 0)	0.03 (0.01 to 0.05)	0 (0 to 0)	0.02 (0.01 to 0.03)	15.91 (7.23 to 27.54)	-0.47 (-0.71 to -0.15)	-1.16 (-1.52 to -0.79)
Saint Lucia	0 (0 to 0)	0.04 (0.02 to 0.08)	0 (0 to 0)	0.04 (0.02 to 0.06)	38.51 (20.01 to 65.85)	-0.47 (-0.67 to -0.15)	-1.55 (-2 to -1.11)
Saint Vincent and the Grenadines	0 (0 to 0)	0.02 (0.01 to 0.04)	0 (0 to 0)	0.02 (0.01 to 0.04)	23.21 (12.01 to 38.55)	-0.31 (-0.56 to 0.09)	0.21 (-0.14 to 0.57)

Samoa	0 (0 to 0)	0.07 (0.03 to 0.14)	0 (0 to 0)	0.03 (0.01 to 0.06)	36.58 (16.16 to 67.76)	-0.54 (-0.75 to -0.22)	-2.71 (-2.84 to -2.58)
San Marino	0 (0 to 0)	0 (0 to 0.01)	0 (0 to 0)	0 (0 to 0)	8.85 (3.99 to 15.58)	-0.31 (-0.6 to 0.22)	-1.53 (-1.72 to -1.34)
Sao Tome and Principe	0 (0 to 0)	0.08 (0.03 to 0.14)	0 (0 to 0)	0.03 (0.01 to 0.05)	17.59 (8.08 to 31.81)	-0.48 (-0.71 to -0.07)	-4.06 (-4.6 to -3.52)
Saudi Arabia	3 (1 to 6)	0.04 (0.02 to 0.07)	2 (1 to 3)	0.02 (0.01 to 0.03)	34.76 (17.6 to 58.5)	-0.45 (-0.66 to -0.07)	-2.71 (-3.03 to -2.4)
Senegal	2 (1 to 4)	0.04 (0.02 to 0.09)	1 (1 to 3)	0.02 (0.01 to 0.04)	5.36 (2.3 to 10.24)	-0.27 (-0.63 to 0.53)	-3.19 (-3.51 to -2.87)
Serbia	0 (0 to 1)	0.01 (0.01 to 0.02)	0 (0 to 0)	0 (0 to 0)	9.02 (4.49 to 15.26)	-0.88 (-0.92 to -0.8)	-6.68 (-7.12 to -6.24)
Seychelles	0 (0 to 0)	0.01 (0.01 to 0.02)	0 (0 to 0)	0.01 (0.01 to 0.02)	13.99 (7.03 to 23.18)	-0.1 (-0.41 to 0.34)	-0.07 (-0.56 to 0.43)
Sierra Leone	1 (0 to 3)	0.07 (0.02 to 0.15)	3 (1 to 6)	0.06 (0.02 to 0.15)	8.56 (3.14 to 18.29)	0.96 (-0.13 to 3.46)	-0.42 (-0.76 to -0.08)
Singapore	0 (0 to 0)	0.01 (0.01 to 0.02)	0 (0 to 0)	0 (0 to 0.01)	12.21 (4.91 to 27.75)	-0.81 (-0.89 to -0.58)	-6.99 (-7.37 to -6.61)
Slovakia	0 (0 to 0)	0 (0 to 0.01)	0 (0 to 0)	0 (0 to 0)	3.31 (1.52 to 5.97)	-0.72 (-0.83 to -0.54)	-3.06 (-3.28 to -2.84)
Slovenia	0 (0 to 0)	0 (0 to 0)	0 (0 to 0)	0 (0 to 0)	4.76 (2.21 to 8.45)	-0.72 (-0.82 to -0.55)	-4.49 (-5.2 to -3.77)
Solomon Islands	0 (0 to 0)	0.02 (0.01 to 0.05)	0 (0 to 0)	0.03 (0.01 to 0.05)	11.87 (5.37 to 21.97)	0.92 (0.19 to 2.09)	0.47 (-0.01 to 0.94)
Somalia	5 (1 to 11)	0.11 (0.03 to 0.26)	5 (2 to 12)	0.04 (0.02 to 0.1)	5.58 (2.22 to 11.8)	0.06 (-0.5 to 1.58)	-2.88 (-3.21 to -2.54)
South Africa	19 (8 to 34)	0.11 (0.04 to 0.19)	7 (3 to 11)	0.03 (0.02 to 0.06)	12.69 (6.63 to 19.99)	-0.65 (-0.78 to -0.39)	-3.72 (-4.08 to -3.36)
South Sudan	10 (3 to 23)	0.32 (0.1 to 0.7)	7 (3 to 14)	0.13 (0.05 to 0.26)	18.55 (7.75 to 35.63)	-0.34 (-0.68 to 0.48)	-2.86 (-3.34 to -2.38)

Spain	1 (1 to 2)	0.01 (0 to 0.02)	0 (0 to 0)	0 (0 to 0)	12.88 (6.02 to 21.78)	-0.76 (-0.85 to -0.63)	-4.92 (-5.28 to -4.56)
Sri Lanka	3 (2 to 5)	0.04 (0.02 to 0.07)	1 (1 to 2)	0.02 (0.01 to 0.03)	27.18 (13.52 to 48.3)	-0.65 (-0.79 to -0.4)	-3.28 (-3.68 to -2.88)
Sudan	8 (2 to 19)	0.07 (0.02 to 0.17)	7 (3 to 13)	0.03 (0.01 to 0.07)	11.89 (5.24 to 21.8)	-0.16 (-0.58 to 1.72)	-2.36 (-2.55 to -2.17)
Suriname	0 (0 to 0)	0.03 (0.01 to 0.06)	0 (0 to 0)	0.03 (0.01 to 0.05)	15.97 (7.65 to 26.93)	-0.11 (-0.44 to 0.69)	-0.83 (-1.11 to -0.55)
Sweden	0 (0 to 0)	0.01 (0 to 0.01)	0 (0 to 0)	0 (0 to 0.01)	15.35 (7.7 to 25.13)	-0.42 (-0.59 to -0.17)	-1.78 (-2.15 to -1.42)
Switzerland	0 (0 to 0)	0.01 (0 to 0.01)	0 (0 to 0)	0 (0 to 0)	8.4 (3.98 to 14.43)	-0.7 (-0.82 to -0.54)	-5.22 (-5.48 to -4.96)
Syrian Arab Republic	7 (3 to 12)	0.09 (0.04 to 0.17)	6 (3 to 9)	0.09 (0.04 to 0.16)	96.2 (45.72 to 156.5)	-0.22 (-0.52 to 0.3)	1.13 (0.5 to 1.77)
Taiwan (Province of China)	1 (0 to 1)	0.01 (0 to 0.01)	0 (0 to 0)	0 (0 to 0)	6.22 (3.12 to 10.85)	-0.86 (-0.91 to -0.78)	-6.44 (-6.99 to -5.88)
Tajikistan	0 (0 to 0)	0 (0 to 0)	0 (0 to 0)	0 (0 to 0)	0.19 (0.08 to 0.38)	0.75 (-0.11 to 2.36)	-0.55 (-1.07 to -0.03)
Thailand	5 (2 to 9)	0.02 (0.01 to 0.04)	3 (1 to 5)	0.02 (0.01 to 0.03)	25.23 (12.77 to 41.84)	-0.44 (-0.62 to -0.14)	-0.51 (-0.78 to -0.23)
Timor-Leste	1 (0 to 1)	0.14 (0.03 to 0.32)	0 (0 to 1)	0.05 (0.01 to 0.09)	21.12 (5.71 to 41.53)	-0.42 (-0.83 to 1.55)	-3.28 (-3.74 to -2.83)
Togo	1 (0 to 1)	0.03 (0.01 to 0.05)	1 (0 to 2)	0.02 (0.01 to 0.04)	4.86 (2.08 to 9.28)	0.51 (-0.21 to 1.68)	-1.28 (-1.69 to -0.88)
Tokelau	0 (0 to 0)	0.06 (0.02 to 0.12)	0 (0 to 0)	0.02 (0.01 to 0.04)	32.79 (14.77 to 58.27)	-0.71 (-0.84 to -0.44)	-3.05 (-3.43 to -2.66)
Tonga	0 (0 to 0)	0.04 (0.02 to 0.07)	0 (0 to 0)	0.03 (0.01 to 0.06)	31.25 (15.06 to 55.22)	-0.31 (-0.61 to 0.09)	-1.26 (-1.81 to -0.7)

Trinidad and Tobago	1 (0 to 1)	0.11 (0.05 to 0.17)	0 (0 to 0)	0.07 (0.04 to 0.12)	74.15 (38.99 to 118.6)	-0.53 (-0.7 to -0.27)	-1.43 (-1.67 to -1.19)
Tunisia	1 (1 to 3)	0.04 (0.02 to 0.06)	0 (0 to 1)	0.01 (0.01 to 0.02)	16.31 (7.84 to 29.83)	-0.7 (-0.82 to -0.45)	-3.26 (-3.4 to -3.12)
Turkey	15 (7 to 28)	0.05 (0.02 to 0.1)	2 (1 to 5)	0.01 (0 to 0.02)	11.76 (5.23 to 26.33)	-0.84 (-0.92 to -0.67)	-6.23 (-6.78 to -5.68)
Turkmenistan	1 (0 to 2)	0.05 (0.02 to 0.1)	0 (0 to 0)	0.01 (0 to 0.01)	4.26 (2.17 to 7.08)	-0.85 (-0.91 to -0.72)	-7.32 (-7.73 to -6.91)
Tuvalu	0 (0 to 0)	0.14 (0.06 to 0.29)	0 (0 to 0)	0.05 (0.03 to 0.1)	51.86 (25.91 to 93.11)	-0.55 (-0.77 to 0)	-2.98 (-3.15 to -2.8)
Uganda	16 (6 to 35)	0.16 (0.06 to 0.34)	16 (6 to 32)	0.06 (0.03 to 0.14)	14.47 (5.89 to 29.21)	-0.05 (-0.54 to 1.11)	-2.58 (-3.01 to -2.14)
Ukraine	0 (0 to 1)	0 (0 to 0.01)	0 (0 to 0)	0 (0 to 0)	1.73 (0.74 to 3.33)	-0.73 (-0.85 to -0.46)	-4.33 (-5.61 to -3.03)
United Arab Emirates	1 (0 to 1)	0.08 (0.04 to 0.14)	0 (0 to 1)	0.03 (0.01 to 0.05)	67.43 (30.92 to 128.74)	-0.23 (-0.55 to 0.32)	-3.79 (-4.3 to -3.27)
United Kingdom	4 (2 to 6)	0.03 (0.02 to 0.04)	2 (1 to 3)	0.01 (0.01 to 0.02)	49.23 (26.73 to 72.48)	-0.44 (-0.51 to -0.35)	-1.91 (-2.25 to -1.57)
United Republic of Tanzania	16 (6 to 32)	0.1 (0.04 to 0.21)	20 (8 to 44)	0.07 (0.03 to 0.14)	15.07 (6.21 to 30.25)	0.32 (-0.35 to 2.17)	-0.74 (-1.2 to -0.27)
United States of America	16 (9 to 23)	0.02 (0.01 to 0.03)	20 (12 to 29)	0.02 (0.01 to 0.04)	48.4 (28.31 to 70.18)	0.24 (0.06 to 0.44)	-0.16 (-0.42 to 0.11)
United States Virgin Islands	0 (0 to 0)	0.03 (0.02 to 0.05)	0 (0 to 0)	0.02 (0.01 to 0.03)	34.03 (17.2 to 57.55)	-0.61 (-0.75 to -0.37)	-2.27 (-2.91 to -1.63)
Uruguay	0 (0 to 0)	0.01 (0.01 to 0.02)	0 (0 to 0)	0.01 (0.01 to 0.02)	16.98 (8 to 28.97)	-0.1 (-0.43 to 0.41)	-0.02 (-0.25 to 0.2)

Uzbekistan	1 (0 to 2)	0.01 (0 to 0.01)	1 (0 to 1)	0 (0 to 0.01)	2.66 (1.17 to 5.41)	-0.42 (-0.65 to -0.03)	-2.9 (-3.26 to -2.53)
Vanuatu	0 (0 to 0)	0.05 (0.02 to 0.1)	0 (0 to 0)	0.06 (0.03 to 0.11)	31.39 (14.28 to 58.14)	0.9 (0.17 to 1.94)	0.23 (-0.14 to 0.6)
Venezuela (Bolivarian Republic of)	6 (3 to 10)	0.07 (0.03 to 0.11)	2 (1 to 3)	0.02 (0.01 to 0.03)	14.62 (7.38 to 25.11)	-0.72 (-0.83 to -0.49)	-5.32 (-6.21 to -4.42)
Viet Nam	3 (1 to 5)	0.01 (0 to 0.01)	3 (1 to 5)	0.01 (0 to 0.02)	10.19 (4.45 to 18.33)	0.04 (-0.42 to 1.07)	1.58 (1.24 to 1.93)
Yemen	5 (1 to 11)	0.06 (0.02 to 0.13)	2 (1 to 4)	0.01 (0.01 to 0.03)	3.97 (1.87 to 7.48)	-0.53 (-0.78 to 0.33)	-3.94 (-4.66 to -3.21)
Zambia	13 (4 to 27)	0.27 (0.08 to 0.57)	8 (4 to 17)	0.09 (0.04 to 0.18)	21.6 (9.36 to 42.26)	-0.34 (-0.7 to 1)	-3.47 (-3.75 to -3.19)
Zimbabwe	1 (0 to 2)	0.02 (0.01 to 0.04)	2 (1 to 3)	0.02 (0.01 to 0.04)	5.41 (2.29 to 10.16)	0.36 (-0.23 to 1.49)	2.04 (1.06 to 3.03)

BMI, body mass index;
PAF, population attribuTable fraction;
EAPC, estimated annual percentage change;
95% UI, 95% uncertainty interval;
95% CI, certain interval.

Supplementary Table 5. DALYs burden attribuTable to high BMI in children and adolescents at national level from 1990 to 2019

Location	DALYs cases (95% UI), 1990	DALYs rate per 100000 (95% UI), 1990	DALYs cases (95% UI), 2019	DALYs rate per 100000 (95% UI), 2019	DALYs PAF per 100000 (95% UI), 2019	DALYs cases change (95% UI) (%), 1990-2019	EAPC of DALYs rate (95% CI), 1990-2019
Afghanistan	348 (152 to 623)	5.45 (2.37 to 9.75)	923 (419 to 1640)	4.27 (1.94 to 7.59)	9.7 (4.38 to 17.12)	1.65 (0.65 to 3.01)	-0.26 (-1.15 to 0.64)
Albania	92 (41 to 166)	6.31 (2.83 to 11.4)	57 (24 to 108)	8.5 (3.66 to 16.22)	66.85 (29.8 to 124.78)	-0.38 (-0.56 to -0.15)	1.06 (0.72 to 1.41)
Algeria	970 (461 to 1681)	7.18 (3.41 to 12.44)	1889 (835 to 3564)	12.78 (5.65 to 24.12)	74.9 (33.94 to 137.86)	0.95 (0.41 to 1.59)	2.27 (2.04 to 2.51)
American Samoa	4 (2 to 7)	17.23 (8.3 to 31.18)	5 (2 to 9)	20.24 (9.15 to 38.4)	186.29 (88.33 to 338.73)	0.12 (-0.1 to 0.37)	0.74 (0.57 to 0.91)
Andorra	2 (1 to 5)	18.43 (8.25 to 35.76)	3 (1 to 5)	18.41 (8.18 to 35.11)	291.03 (134.68 to 519.02)	0.11 (-0.09 to 0.32)	0.03 (-0.12 to 0.18)
Angola	797 (263 to 1739)	13.74 (4.54 to 29.98)	1996 (918 to 3685)	11.7 (5.38 to 21.61)	28.81 (13.1 to 53.01)	1.51 (0.25 to 4.09)	-0.99 (-1.52 to -0.45)
Antigua and Barbuda	3 (1 to 6)	12.02 (4.96 to 24.2)	5 (2 to 10)	20.69 (8.58 to 40.89)	192.58 (85.58 to 365.14)	0.67 (0.37 to 1.03)	1.64 (1.53 to 1.74)
Argentina	883 (382 to 1687)	6.78 (2.94 to 12.96)	1947 (835 to 3945)	13.75 (5.9 to 27.88)	121.82 (54.68 to 235.95)	1.21 (0.72 to 1.9)	2.53 (2.35 to 2.72)
Armenia	51 (20 to 100)	3.81 (1.54 to 7.57)	57 (23 to 115)	7.34 (2.99 to 14.88)	67.09 (28.44 to 135.04)	0.13 (-0.09 to 0.39)	2.66 (2.32 to 3)
Australia	1678 (757 to 2993)	32.43 (14.63 to 57.85)	1942 (873 to 3828)	32.26 (14.51 to 63.56)	432.54 (202.85 to 788.45)	0.16 (-0.14 to 0.57)	-0.64 (-0.87 to -0.4)
Austria	152 (67 to 281)	8.14 (3.57 to 15.02)	222 (98 to 442)	12.76 (5.64 to 25.38)	179.96 (85.41 to 329.02)	0.46 (0.14 to 0.88)	1.68 (1.57 to 1.79)

Azerbaijan	138 (57 to 273)	4.39 (1.82 to 8.71)	260 (111 to 504)	8.67 (3.71 to 16.81)	44.34 (18.83 to 88.13)	0.89 (0.55 to 1.3)	2.73 (2.19 to 3.28)
Bahamas	25 (11 to 48)	22.81 (10.15 to 44.56)	32 (14 to 63)	29.22 (12.82 to 57.43)	234.9 (107.79 to 444.82)	0.32 (0.09 to 0.6)	0.96 (0.81 to 1.1)
Bahrain	33 (16 to 56)	16.16 (7.72 to 28.05)	60 (28 to 114)	18.69 (8.68 to 35.52)	211.6 (101.49 to 373.94)	0.84 (0.45 to 1.3)	0.37 (0.26 to 0.48)
Bangladesh	771 (347 to 1363)	1.28 (0.58 to 2.27)	453 (205 to 837)	0.76 (0.35 to 1.41)	3.73 (1.72 to 6.65)	-0.41 (-0.64 to -0.04)	-2.18 (-2.34 to -2.02)
Barbados	22 (10 to 42)	26.35 (11.84 to 49.28)	22 (10 to 42)	31.54 (14.07 to 61.28)	268.88 (124.52 to 502.82)	-0.03 (-0.22 to 0.21)	0.56 (0.48 to 0.64)
Belarus	168 (70 to 332)	5.34 (2.21 to 10.53)	168 (69 to 334)	8.06 (3.29 to 16.02)	107.55 (46.96 to 208.46)	0 (-0.19 to 0.23)	0.88 (0.37 to 1.4)
Belgium	214 (96 to 406)	8.67 (3.89 to 16.43)	300 (133 to 594)	11.72 (5.18 to 23.23)	167.31 (78.03 to 300.25)	0.4 (0.09 to 0.82)	1.17 (0.94 to 1.41)
Belize	16 (7 to 29)	15.48 (6.97 to 28.27)	44 (20 to 83)	26.48 (11.99 to 49.44)	185.25 (87.59 to 340.11)	1.82 (1.19 to 2.61)	1.5 (1.15 to 1.85)
Benin	173 (76 to 315)	6.01 (2.65 to 10.97)	672 (302 to 1207)	9.46 (4.25 to 17)	15.06 (6.89 to 27.53)	2.89 (1.46 to 4.93)	1.53 (1.18 to 1.88)
Bermuda	5 (2 to 10)	31.42 (13.52 to 61.37)	5 (2 to 9)	40.22 (17.51 to 77.41)	537.03 (253.61 to 982.37)	-0.04 (-0.19 to 0.13)	0.88 (0.76 to 1.01)
Bhutan	7 (2 to 15)	2.15 (0.73 to 4.5)	6 (3 to 12)	2.44 (1.13 to 4.47)	10.32 (4.84 to 18.05)	-0.08 (-0.5 to 1.03)	0.27 (0.13 to 0.41)
Bolivia (Plurinational State of)	2389 (966 to 4489)	70.48 (28.52 to 132.46)	1455 (647 to 2740)	29.13 (12.95 to 54.86)	118.1 (53.13 to 224.11)	-0.39 (-0.65 to 0.02)	-3.73 (-4.17 to -3.28)
Bosnia and Herzegovina	75 (31 to 147)	5.01 (2.09 to 9.83)	73 (31 to 140)	10.96 (4.71 to 21.14)	143.87 (64.83 to 269.36)	-0.03 (-0.2 to 0.18)	3.28 (2.99 to 3.56)

Botswana	34 (15 to 60)	4.65 (2.09 to 8.27)	92 (40 to 168)	10.05 (4.34 to 18.28)	34.25 (14.84 to 62.61)	1.75 (0.73 to 3.12)	3.07 (2.81 to 3.33)
Brazil	11919 (5394 to 21544)	17.64 (7.98 to 31.89)	18662 (8227 to 35221)	29.07 (12.81 to 54.86)	167.5 (76.27 to 313.34)	0.57 (0.22 to 1)	1.55 (1.39 to 1.72)
Brunei Darussalam	4 (2 to 8)	3.76 (1.64 to 7.24)	9 (4 to 18)	7.1 (3.06 to 14.06)	70.18 (31.32 to 132.93)	1.15 (0.71 to 1.7)	2.4 (2.23 to 2.56)
Bulgaria	213 (91 to 413)	9.03 (3.86 to 17.49)	130 (57 to 257)	9.95 (4.35 to 19.67)	109.45 (48.98 to 203.85)	-0.39 (-0.5 to -0.26)	0.34 (-0.18 to 0.87)
Burkina Faso	335 (140 to 635)	5.92 (2.47 to 11.21)	1399 (630 to 2558)	10.96 (4.93 to 20.03)	13.57 (5.86 to 25.18)	3.17 (1.56 to 5.76)	1.97 (1.7 to 2.24)
Burundi	637 (255 to 1258)	20.08 (8.03 to 39.66)	704 (309 to 1351)	10.57 (4.64 to 20.29)	20.78 (9.26 to 38.56)	0.1 (-0.38 to 1.13)	-2.18 (-2.44 to -1.93)
Cabo Verde	7 (3 to 12)	3.45 (1.57 to 6.46)	16 (7 to 31)	7.67 (3.32 to 14.92)	49.44 (21.78 to 94.67)	1.42 (0.73 to 2.23)	2.52 (2.43 to 2.61)
Cambodia	154 (60 to 288)	2.67 (1.04 to 4.99)	245 (113 to 451)	3.76 (1.73 to 6.92)	15.67 (6.95 to 28.46)	0.59 (-0.08 to 2.03)	1.27 (0.97 to 1.57)
Cameroon	490 (221 to 865)	8.31 (3.74 to 14.68)	1674 (771 to 3040)	10.97 (5.05 to 19.93)	22.65 (10.8 to 40.84)	2.42 (1.37 to 3.81)	0.63 (0.51 to 0.76)
Canada	1240 (541 to 2354)	16.18 (7.05 to 30.7)	1731 (750 to 3390)	21.29 (9.23 to 41.7)	287.78 (135.57 to 527.42)	0.4 (0.13 to 0.72)	0.69 (0.05 to 1.33)
Central African Republic	295 (114 to 570)	19.64 (7.59 to 37.97)	340 (143 to 685)	12.05 (5.07 to 24.3)	13.12 (5.38 to 25.75)	0.15 (-0.3 to 0.89)	-1.66 (-1.93 to -1.39)
Chad	126 (52 to 244)	3.58 (1.48 to 6.92)	571 (258 to 1051)	5.66 (2.55 to 10.41)	6.47 (2.91 to 11.71)	3.53 (1.76 to 6.43)	0.94 (0.5 to 1.38)
Chile	378 (162 to 714)	7.17 (3.07 to 13.52)	841 (365 to 1613)	17.29 (7.5 to 33.16)	201.56 (92.42 to 372.36)	1.22 (0.74 to 1.83)	2.82 (2.34 to 3.3)
China	7498 (3315 to 14152)	1.67 (0.74 to 3.15)	14775 (5965 to 29534)	4.93 (1.99 to 9.85)	54.74 (23.62 to 104.76)	0.97 (0.65 to 1.24)	3.68 (2.71 to 4.65)

Colombia	1542 (663 to 2950)	10.21 (4.39 to 19.52)	1972 (836 to 3877)	13.14 (5.57 to 25.83)	100.1 (43.9 to 190.22)	0.28 (0 to 0.62)	0.71 (0.49 to 0.93)
Comoros	69 (28 to 134)	26.16 (10.45 to 50.86)	59 (28 to 108)	19.34 (9.13 to 35.45)	56.77 (26.17 to 104.34)	-0.15 (-0.47 to 0.51)	-1.51 (-1.72 to -1.29)
Congo	150 (67 to 275)	10.92 (4.92 to 20.11)	266 (123 to 493)	10.66 (4.9 to 19.75)	34.31 (16.1 to 62.51)	0.78 (0.13 to 1.59)	-0.57 (-0.87 to -0.28)
Cook Islands	1 (1 to 3)	17.11 (7.77 to 32.49)	1 (1 to 3)	24.62 (10.94 to 47.61)	376.39 (178.33 to 676.18)	-0.09 (-0.26 to 0.11)	1.36 (1.25 to 1.48)
Costa Rica	413 (181 to 801)	29.13 (12.76 to 56.54)	607 (269 to 1197)	42.87 (19.01 to 84.49)	455.4 (212.24 to 841.39)	0.47 (0.17 to 0.83)	1.07 (0.73 to 1.41)
C 么 te d'Ivoire	279 (126 to 506)	4.01 (1.81 to 7.29)	1223 (568 to 2244)	9.18 (4.26 to 16.84)	16.78 (7.75 to 30.1)	3.39 (1.85 to 5.25)	2.64 (2.09 to 3.19)
Croatia	88 (37 to 171)	6.64 (2.79 to 12.91)	83 (34 to 160)	10.09 (4.16 to 19.43)	149.15 (65.06 to 273.56)	-0.06 (-0.22 to 0.16)	1.58 (1.34 to 1.81)
Cuba	1014 (438 to 1953)	27.89 (12.05 to 53.69)	976 (427 to 1898)	39.37 (17.24 to 76.54)	495.93 (234.18 to 885.39)	-0.04 (-0.19 to 0.15)	1.13 (0.93 to 1.33)
Cyprus	23 (10 to 45)	9.02 (3.83 to 17.34)	39 (17 to 73)	13.85 (6.06 to 26.12)	196.13 (89.43 to 353.67)	0.65 (0.34 to 1.03)	1.71 (1.55 to 1.87)
Czechia	175 (78 to 330)	5.76 (2.56 to 10.86)	186 (79 to 369)	8.56 (3.62 to 16.95)	140.95 (64.23 to 258.07)	0.06 (-0.14 to 0.3)	1.22 (1.13 to 1.31)
Democratic People's Republic of Korea	169 (72 to 329)	2.03 (0.86 to 3.94)	143 (60 to 279)	2.13 (0.89 to 4.17)	17.15 (7.27 to 33.15)	-0.16 (-0.4 to 0.14)	0.09 (-0.06 to 0.24)
Democratic Republic of the Congo	2934 (1049 to 6172)	13.29 (4.75 to 27.95)	4111 (1911 to 7650)	8.7 (4.04 to 16.18)	20.1 (9.29 to 36.97)	0.4 (-0.22 to 1.71)	-1.67 (-2.01 to -1.33)
Denmark	117 (52 to 229)	9.38 (4.18 to 18.29)	171 (76 to 332)	13.16 (5.79 to 25.47)	190.31 (89.32 to 347.98)	0.46 (0.21 to 0.76)	1.33 (1.11 to 1.55)
Djibouti	33 (14 to 59)	11.87 (5.18 to 21.32)	86 (40 to 156)	16.48 (7.6 to 29.71)	43.88 (20.21 to 79.78)	1.65 (0.77 to 2.85)	1.02 (0.69 to 1.34)

Dominica	8 (3 to 14)	23.67 (10.59 to 43.85)	9 (4 to 16)	43.83 (20.09 to 79.44)	242.68 (114.07 to 443.87)	0.16 (-0.03 to 0.4)	2.09 (1.73 to 2.46)
Dominican Republic	822 (377 to 1446)	23.13 (10.6 to 40.7)	863 (391 to 1562)	21.49 (9.74 to 38.91)	105.4 (47.72 to 189.63)	0.05 (-0.3 to 0.51)	0.21 (-0.29 to 0.72)
Ecuador	2153 (963 to 3807)	43.05 (19.27 to 76.14)	1614 (682 to 3160)	24.13 (10.2 to 47.26)	166.66 (73.68 to 319.17)	-0.25 (-0.5 to 0.13)	-2.12 (-2.96 to -1.27)
Egypt	8415 (3827 to 14580)	30.09 (13.68 to 52.13)	7645 (3516 to 13975)	18.24 (8.39 to 33.34)	128.49 (60.09 to 228.52)	-0.09 (-0.45 to 0.49)	-1.75 (-2.2 to -1.3)
El Salvador	766 (342 to 1375)	28.14 (12.56 to 50.53)	469 (206 to 926)	20.47 (8.98 to 40.4)	164.6 (74.13 to 312.8)	-0.39 (-0.6 to -0.1)	-1.2 (-1.45 to -0.96)
Equatorial Guinea	37 (13 to 77)	14.8 (5.21 to 31.1)	171 (81 to 320)	22.93 (10.87 to 42.87)	82.05 (37.17 to 154.23)	3.67 (1.39 to 8.59)	1.34 (1.05 to 1.62)
Eritrea	207 (82 to 409)	11.95 (4.72 to 23.54)	355 (159 to 636)	10.42 (4.68 to 18.7)	29.91 (13.46 to 53.55)	0.71 (-0.02 to 1.94)	-0.41 (-0.63 to -0.18)
Estonia	21 (9 to 39)	4.48 (1.97 to 8.48)	22 (9 to 43)	7.83 (3.25 to 15.69)	124.93 (56.11 to 236.37)	0.05 (-0.2 to 0.39)	1.5 (1.18 to 1.82)
Eswatini	82 (40 to 147)	17.29 (8.35 to 30.85)	83 (41 to 144)	15.59 (7.71 to 27.08)	42.79 (21.03 to 74.76)	0 (-0.31 to 0.39)	-0.5 (-0.62 to -0.37)
Ethiopia	2216 (872 to 4286)	7.39 (2.91 to 14.3)	4131 (1934 to 7547)	7.05 (3.3 to 12.87)	18.62 (8.62 to 33.54)	0.86 (0.11 to 2.45)	-0.3 (-0.76 to 0.15)
Fiji	58 (29 to 95)	16.01 (8.14 to 26.5)	68 (35 to 113)	19.91 (10.34 to 33.06)	108.97 (57.04 to 177.44)	0.18 (-0.16 to 0.57)	-0.08 (-0.63 to 0.47)
Finland	133 (58 to 254)	10.45 (4.59 to 20.01)	161 (69 to 320)	13.77 (5.93 to 27.28)	213.16 (99.41 to 381.62)	0.22 (-0.02 to 0.5)	1.01 (0.86 to 1.16)
France	1634 (714 to 3071)	10.13 (4.43 to 19.03)	2548 (1141 to 4933)	16 (7.16 to 30.97)	224.39 (104.88 to 406.1)	0.56 (0.19 to 1.04)	1.32 (1.13 to 1.51)
Gabon	42 (19 to 75)	8.12 (3.61 to 14.52)	90 (41 to 168)	11.86 (5.38 to 22.1)	47.68 (21.24 to 88.18)	1.14 (0.38 to 2.14)	1.17 (1.04 to 1.29)
Gambia	24 (10 to 44)	4.24 (1.84 to 7.78)	72 (32 to 131)	6.08 (2.74 to 11.09)	20.01 (8.94 to 36.62)	1.97 (0.92 to 3.47)	0.77 (0.52 to 1.02)

Georgia	162 (76 to 286)	9.03 (4.24 to 15.92)	112 (51 to 207)	12.11 (5.56 to 22.45)	113.06 (53.02 to 207.33)	-0.31 (-0.47 to -0.12)	1.24 (0.94 to 1.53)
Germany	1666 (768 to 3062)	9.61 (4.43 to 17.66)	1922 (841 to 3805)	12.23 (5.35 to 24.23)	171.56 (80.8 to 319.2)	0.15 (-0.11 to 0.44)	0.86 (0.66 to 1.06)
Ghana	296 (131 to 567)	3.56 (1.58 to 6.84)	1021 (475 to 1885)	6.99 (3.25 to 12.92)	18.81 (8.55 to 34.46)	2.45 (1.27 to 4.18)	1.98 (1.65 to 2.31)
Greece	271 (111 to 559)	9.66 (3.93 to 19.87)	315 (139 to 614)	15.98 (7.05 to 31.11)	218.84 (101.21 to 401.13)	0.16 (-0.12 to 0.78)	2.31 (1.88 to 2.75)
Greenland	5 (2 to 8)	25.65 (11.64 to 47.21)	4 (2 to 8)	26.77 (11.9 to 49.69)	242.29 (112.47 to 455.6)	-0.11 (-0.27 to 0.09)	-0.35 (-0.56 to -0.15)
Grenada	5 (2 to 10)	13.22 (5.82 to 25.41)	8 (3 to 15)	25.13 (11 to 49.16)	191.97 (86.82 to 362.15)	0.44 (0.16 to 0.81)	1.63 (1.37 to 1.88)
Guam	12 (5 to 23)	22.29 (9.85 to 42.32)	16 (7 to 30)	27.68 (12.35 to 51.8)	214.02 (98.4 to 399.16)	0.35 (0.18 to 0.55)	0.83 (0.66 to 0.99)
Guatemala	1689 (725 to 2872)	37.89 (16.27 to 64.43)	977 (443 to 1826)	12.64 (5.73 to 23.64)	67.84 (30.26 to 127.08)	-0.42 (-0.63 to -0.06)	-4.35 (-5.29 to -3.39)
Guinea	335 (145 to 633)	9.83 (4.25 to 18.59)	708 (321 to 1277)	10.06 (4.56 to 18.14)	14.42 (6.55 to 25.3)	1.12 (0.26 to 2.41)	-0.33 (-0.51 to -0.14)
Guinea-Bissau	42 (17 to 81)	7.13 (2.95 to 13.94)	68 (31 to 123)	6.78 (3.09 to 12.31)	13.09 (6.2 to 23.6)	0.63 (-0.05 to 1.79)	-0.66 (-1.24 to -0.07)
Guyana	54 (24 to 98)	14.48 (6.5 to 26.4)	70 (33 to 131)	24.73 (11.58 to 46.41)	122.88 (57.05 to 230.77)	0.29 (0.05 to 0.62)	1.86 (1.62 to 2.1)
Haiti	1193 (425 to 2471)	35.89 (12.77 to 74.32)	1240 (528 to 2294)	22.54 (9.59 to 41.7)	45.84 (19.61 to 85.42)	0.04 (-0.35 to 0.68)	-1.3 (-1.63 to -0.98)
Honduras	630 (270 to 1152)	23.25 (9.96 to 42.49)	663 (296 to 1232)	15.2 (6.78 to 28.25)	99.18 (45.66 to 184.55)	0.05 (-0.33 to 0.66)	-1.88 (-2.03 to -1.73)
Hungary	214 (92 to 423)	7.37 (3.18 to 14.6)	181 (76 to 358)	9.7 (4.09 to 19.14)	140.2 (61.8 to 262.52)	-0.15 (-0.3 to 0.03)	1.15 (0.74 to 1.55)

Iceland	19 (8 to 36)	22.22 (9.36 to 43)	19 (9 to 37)	21.82 (9.73 to 42.22)	342.82 (159.25 to 606.3)	0.03 (-0.29 to 0.33)	0.29 (-0.03 to 0.61)
India	9190 (4189 to 16084)	2.22 (1.01 to 3.88)	12525 (5413 to 23590)	2.45 (1.06 to 4.62)	10.12 (4.55 to 18.52)	0.36 (-0.1 to 1.01)	1.11 (0.48 to 1.75)
Indonesia	5552 (2462 to 9911)	6.32 (2.8 to 11.28)	8109 (3933 to 14412)	9.21 (4.47 to 16.38)	52.76 (26.03 to 90.63)	0.46 (-0.04 to 1.27)	1.61 (1.37 to 1.85)
Iran (Islamic Republic of)	2551 (1201 to 4514)	7.96 (3.74 to 14.08)	3388 (1514 to 6379)	13.1 (5.85 to 24.67)	105.84 (49.02 to 198.24)	0.33 (-0.02 to 0.72)	1.82 (1.71 to 1.93)
Iraq	1080 (514 to 1929)	10.77 (5.12 to 19.24)	2130 (929 to 4151)	11.71 (5.11 to 22.82)	77.15 (34.25 to 148.9)	0.97 (0.39 to 1.62)	0.52 (-0.03 to 1.07)
Ireland	272 (120 to 500)	20.46 (9.04 to 37.68)	297 (133 to 566)	22.16 (9.97 to 42.28)	319.57 (151.96 to 568.67)	0.09 (-0.13 to 0.33)	-0.06 (-0.36 to 0.24)
Israel	247 (109 to 460)	12.32 (5.44 to 22.95)	482 (212 to 969)	14.54 (6.38 to 29.23)	215.86 (101.57 to 403.61)	0.95 (0.58 to 1.38)	0.42 (0.36 to 0.49)
Italy	962 (436 to 1862)	7.06 (3.2 to 13.67)	1177 (502 to 2320)	10.86 (4.63 to 21.4)	161.1 (73.67 to 299.88)	0.22 (0 to 0.49)	1.58 (1.42 to 1.74)
Jamaica	266 (128 to 484)	24.43 (11.75 to 44.4)	232 (104 to 435)	26.92 (12.06 to 50.38)	214.84 (99.03 to 391.32)	-0.13 (-0.33 to 0.12)	-0.09 (-0.22 to 0.04)
Japan	3153 (1410 to 6056)	9.46 (4.23 to 18.16)	1679 (670 to 3333)	7.78 (3.1 to 15.43)	131.76 (56.57 to 241.1)	0.47 (-0.56 to -0.37)	-1.9 (-2.29 to -1.51)
Jordan	190 (86 to 343)	8.89 (4.05 to 16.09)	816 (361 to 1584)	16.62 (7.36 to 32.26)	129.06 (57.15 to 255.35)	3.31 (2.51 to 4.33)	2.41 (2.31 to 2.52)
Kazakhstan	263 (118 to 479)	3.98 (1.78 to 7.24)	420 (183 to 801)	6.71 (2.91 to 12.79)	54.17 (24.47 to 101.43)	0.6 (0.3 to 0.99)	1.69 (1.33 to 2.04)
Kenya	1225 (567 to 2199)	8.85 (4.1 to 15.89)	2296 (1077 to 4151)	9.17 (4.3 to 16.59)	32.24 (14.84 to 58.95)	0.87 (0.39 to 1.51)	0.28 (-0.14 to 0.7)
Kiribati	7 (3 to 11)	18.69 (9.02 to 31.41)	8 (4 to 13)	14.48 (6.88 to 24.65)	53.41 (25.61 to 91.49)	0.13 (-0.23 to 0.63)	-1.19 (-1.48 to -0.89)

Kuwait	147 (70 to 271)	21.06 (9.96 to 38.86)	309 (137 to 593)	28.16 (12.53 to 54.09)	274.45 (130.59 to 514.12)	1.1 (0.67 to 1.57)	1.74 (1.48 to 2.01)
Kyrgyzstan	111 (46 to 223)	5.29 (2.2 to 10.61)	152 (63 to 313)	5.83 (2.39 to 11.99)	39.64 (16.76 to 77.9)	0.37 (0.12 to 0.66)	-0.04 (-0.45 to 0.37)
Lao People's Democratic Republic	216 (72 to 445)	9.61 (3.19 to 19.77)	207 (98 to 364)	6.99 (3.32 to 12.32)	23.57 (11.65 to 39.39)	-0.04 (-0.49 to 1.27)	-1.11 (-1.28 to -0.94)
Latvia	42 (18 to 81)	5.58 (2.45 to 10.79)	36 (14 to 70)	9.09 (3.7 to 17.91)	129.71 (57.07 to 242.86)	-0.15 (-0.36 to 0.13)	1.88 (1.56 to 2.2)
Lebanon	151 (67 to 280)	9.68 (4.32 to 17.98)	280 (123 to 545)	16.18 (7.11 to 31.57)	147.85 (65.57 to 285)	0.85 (0.45 to 1.28)	1.57 (1.4 to 1.74)
Lesotho	53 (23 to 99)	5.54 (2.37 to 10.27)	74 (34 to 131)	8.36 (3.89 to 14.85)	17.98 (8.54 to 31.52)	0.38 (-0.08 to 1.06)	2.12 (1.75 to 2.49)
Liberia	141 (53 to 266)	12.82 (4.77 to 24.08)	145 (63 to 280)	6.03 (2.61 to 11.67)	14.31 (6.24 to 27.55)	0.02 (-0.44 to 0.94)	-2.09 (-2.81 to -1.36)
Libya	294 (134 to 538)	12.45 (5.7 to 22.78)	325 (147 to 604)	15.79 (7.15 to 29.37)	121.46 (57.41 to 226.58)	0.1 (-0.14 to 0.38)	1.02 (0.88 to 1.15)
Lithuania	39 (17 to 78)	3.56 (1.51 to 7.01)	43 (18 to 86)	7.8 (3.24 to 15.55)	109.59 (48.07 to 205.94)	0.1 (-0.16 to 0.47)	3.48 (3.06 to 3.9)
Luxembourg	12 (5 to 24)	13.87 (6.13 to 27.25)	19 (8 to 37)	14.46 (6.2 to 27.79)	219.8 (101.88 to 389.52)	0.56 (0.29 to 0.93)	-0.14 (-0.24 to -0.03)
Madagascar	2062 (992 to 3427)	30.43 (14.65 to 50.58)	2581 (1180 to 5058)	18.6 (8.51 to 36.46)	45.26 (20.97 to 87.74)	0.25 (-0.14 to 1.02)	-1.71 (-1.94 to -1.49)
Malawi	1591 (576 to 3079)	29.33 (10.62 to 56.77)	1707 (809 to 3070)	16.77 (7.95 to 30.18)	42.95 (20.09 to 77.31)	0.07 (-0.38 to 1.07)	-2.11 (-2.34 to -1.88)
Malaysia	524 (253 to 924)	6.28 (3.03 to 11.07)	1159 (519 to 2241)	11.12 (4.98 to 21.5)	116.01 (54.64 to 213.2)	1.21 (0.61 to 1.96)	1.98 (1.85 to 2.11)
Maldives	7 (3 to 14)	5.5 (2.29 to 10.9)	10 (4 to 18)	6.81 (2.99 to 12.77)	46.45 (21.18 to 87.97)	0.36 (-0.23 to 1.37)	0.77 (0.33 to 1.22)

Mali	92 (39 to 223)	1.86 (0.79 to 4.52)	594 (256 to 1130)	4.65 (2 to 8.85)	5.28 (2.26 to 10.1)	5.46 (3.09 to 7.84)	3.07 (2.87 to 3.26)
Malta	10 (4 to 18)	8.26 (3.67 to 15.58)	13 (6 to 25)	15.25 (6.73 to 29.78)	183.63 (81.25 to 338.92)	0.34 (0.03 to 0.74)	2.15 (1.79 to 2.51)
Marshall Islands	2 (1 to 3)	6.3 (2.91 to 11.28)	2 (1 to 4)	10.4 (4.98 to 18.15)	59.82 (28.7 to 104.21)	0.46 (0.1 to 0.93)	1.34 (1.06 to 1.63)
Mauritania	89 (42 to 160)	7.78 (3.69 to 13.95)	252 (113 to 480)	12.23 (5.47 to 23.27)	41.59 (18.49 to 81.71)	1.83 (1.03 to 2.79)	0.97 (0.75 to 1.2)
Mauritius	45 (22 to 76)	10.48 (5.15 to 17.69)	43 (20 to 76)	13.81 (6.52 to 24.7)	119.67 (58.36 to 204.79)	-0.06 (-0.3 to 0.23)	1 (0.89 to 1.11)
Mexico	7580 (3636 to 13841)	17.45 (8.37 to 31.86)	8416 (3677 to 16077)	19.47 (8.51 to 37.2)	150.23 (68.69 to 285.62)	0.11 (-0.13 to 0.38)	0.15 (-0.16 to 0.47)
Micronesia (Federated States of)	6 (3 to 10)	9.91 (4.72 to 16.7)	5 (2 to 9)	11.36 (5.32 to 20.57)	79.86 (36.61 to 140.3)	-0.17 (-0.41 to 0.12)	0.18 (0.03 to 0.33)
Monaco	1 (0 to 2)	19.22 (8.55 to 36.68)	2 (1 to 3)	23.51 (10.36 to 45.7)	353.85 (169.54 to 633.96)	0.71 (0.47 to 1.01)	0.65 (0.61 to 0.69)
Mongolia	66 (28 to 133)	5.82 (2.5 to 11.71)	86 (37 to 168)	6.98 (2.99 to 13.67)	41.12 (17.2 to 80.81)	0.3 (-0.23 to 0.87)	0.37 (-0.08 to 0.82)
Montenegro	20 (8 to 39)	9.25 (3.91 to 18.08)	18 (8 to 35)	12.1 (5.06 to 23.45)	177.85 (79.74 to 323.86)	-0.09 (-0.24 to 0.08)	1.26 (0.97 to 1.55)
Morocco	1072 (499 to 1914)	8.58 (3.99 to 15.32)	1457 (657 to 2731)	11.62 (5.24 to 21.78)	77.33 (35.43 to 146.82)	0.36 (-0.06 to 0.94)	1.03 (0.83 to 1.23)
Mozambique	1281 (467 to 2755)	17.25 (6.29 to 37.1)	2923 (1362 to 5342)	17.18 (8.01 to 31.4)	31.81 (14.85 to 56.58)	1.28 (0.17 to 3.82)	-0.2 (-0.37 to -0.02)
Myanmar	1116 (435 to 2224)	5.6 (2.18 to 11.16)	1663 (765 to 3026)	8.34 (3.84 to 15.18)	29.85 (14.47 to 49.81)	0.49 (-0.21 to 2.34)	1.77 (1.59 to 1.95)
Namibia	44 (19 to 80)	5.81 (2.49 to 10.62)	60 (28 to 108)	5.58 (2.58 to 10.01)	19.72 (9.24 to 36.27)	0.37 (-0.11 to 1.17)	0.22 (0 to 0.43)

Nauru	1 (1 to 2)	21.57 (10.2 to 38.78)	1 (0 to 2)	18.45 (9.04 to 32.78)	88.21 (43.28 to 157.87)	-0.21 (-0.44 to 0.1)	-1.27 (-1.62 to -0.92)
Nepal	96 (30 to 213)	0.92 (0.29 to 2.05)	119 (53 to 223)	0.95 (0.42 to 1.78)	4.55 (2.03 to 8.37)	0.24 (-0.41 to 1.97)	0.31 (-0.17 to 0.79)
Netherlands	340 (151 to 659)	8.88 (3.95 to 17.2)	439 (193 to 868)	11.64 (5.11 to 23.02)	168.78 (81.04 to 302.45)	0.29 (0.04 to 0.7)	0.61 (0.43 to 0.79)
New Zealand	412 (184 to 771)	37.37 (16.72 to 70.05)	398 (182 to 742)	33.63 (15.38 to 62.65)	396.46 (192.87 to 710.6)	-0.03 (-0.19 to 0.17)	-0.79 (-0.96 to -0.61)
Nicaragua	472 (212 to 846)	20.93 (9.39 to 37.52)	436 (197 to 834)	16.59 (7.51 to 31.72)	131.63 (61.31 to 239.92)	-0.08 (-0.37 to 0.36)	-0.95 (-1.22 to -0.68)
Niger	352 (135 to 716)	7.27 (2.78 to 14.79)	954 (413 to 1831)	6.55 (2.83 to 12.58)	7.77 (3.44 to 14.67)	1.71 (0.54 to 3.79)	-0.73 (-1.06 to -0.41)
Nigeria	2746 (1238 to 4917)	5.63 (2.54 to 10.09)	12228 (5565 to 22225)	10.38 (4.72 to 18.87)	15.1 (6.92 to 27.93)	3.45 (2.39 to 4.8)	2.12 (1.93 to 2.32)
Niue	0 (0 to 0)	15.03 (6.91 to 27.16)	0 (0 to 0)	22.49 (10.72 to 41.24)	147.93 (70.18 to 272.6)	-0.23 (-0.41 to -0.01)	1.55 (1.43 to 1.66)
North Macedonia	49 (21 to 96)	6.9 (2.91 to 13.51)	49 (21 to 96)	10.48 (4.51 to 20.55)	113.81 (50.3 to 210.59)	0 (-0.17 to 0.23)	1.69 (1.51 to 1.87)
Northern Mariana Islands	4 (2 to 7)	22.58 (10.2 to 43.31)	3 (1 to 5)	23.14 (10.39 to 43.92)	215.3 (100.67 to 385.82)	-0.27 (-0.39 to -0.14)	0.17 (0.03 to 0.31)
Norway	147 (63 to 279)	13.2 (5.65 to 25.07)	223 (95 to 433)	17.75 (7.55 to 34.52)	266.4 (126.77 to 483.55)	0.52 (0.3 to 0.8)	1.12 (0.66 to 1.59)
Oman	39 (16 to 75)	3.93 (1.62 to 7.53)	241 (100 to 473)	18.76 (7.79 to 36.91)	147.6 (63.89 to 280.93)	5.16 (3.74 to 7.29)	5.95 (5.66 to 6.24)
Pakistan	2036 (876 to 3905)	3.25 (1.4 to 6.24)	2390 (1145 to 4078)	2.14 (1.02 to 3.65)	4.84 (2.33 to 8.5)	0.17 (-0.25 to 0.84)	-1.13 (-1.28 to -0.97)
Palau	1 (0 to 2)	15.94 (7.28 to 30.19)	1 (0 to 2)	19.68 (8.93 to 37.02)	150.42 (69.22 to 279.19)	-0.12 (-0.34 to 0.13)	0.73 (0.62 to 0.84)

Palestine	52 (23 to 97)	4.26 (1.86 to 7.98)	207 (89 to 414)	8.72 (3.75 to 17.44)	70.54 (30.91 to 138.02)	2.98 (1.95 to 4.31)	2.71 (2.51 to 2.9)
Panama	173 (77 to 316)	15.9 (7.11 to 29.05)	287 (127 to 538)	19.24 (8.5 to 36)	146.39 (66.96 to 266.3)	0.66 (0.22 to 1.3)	0.58 (0.29 to 0.88)
Papua New Guinea	272 (121 to 492)	12.88 (5.74 to 23.31)	568 (270 to 1030)	12.26 (5.83 to 22.22)	28.22 (13.28 to 51.59)	1.09 (0.43 to 1.98)	-0.47 (-0.8 to -0.13)
Paraguay	311 (137 to 592)	14.98 (6.6 to 28.55)	765 (331 to 1483)	29.24 (12.65 to 56.72)	215.88 (97.27 to 406.41)	1.46 (0.96 to 2.14)	2.69 (2.52 to 2.85)
Peru	4863 (2152 to 8670)	45.25 (20.03 to 80.68)	3986 (1687 to 8090)	33.22 (14.06 to 67.43)	231.45 (98.99 to 460.09)	-0.18 (-0.46 to 0.18)	-1.47 (-2.02 to -0.92)
Philippines	2641 (1243 to 4549)	8.18 (3.85 to 14.09)	5330 (2605 to 9189)	11.51 (5.63 to 19.84)	59.18 (29.51 to 100.24)	1.02 (0.5 to 1.77)	1.6 (1.3 to 1.89)
Poland	939 (398 to 1846)	7.56 (3.2 to 14.86)	1014 (418 to 2011)	13.15 (5.43 to 26.09)	196.42 (86.95 to 360.96)	0.08 (-0.09 to 0.3)	1.55 (1.42 to 1.68)
Portugal	335 (140 to 649)	11.25 (4.7 to 21.77)	394 (176 to 771)	20 (8.91 to 39.14)	283.69 (134.37 to 504.92)	0.18 (-0.12 to 0.67)	2.08 (1.78 to 2.39)
Puerto Rico	576 (257 to 1080)	43.2 (19.26 to 81.05)	437 (191 to 850)	57.93 (25.37 to 112.68)	661.59 (317.39 to 1192.83)	-0.24 (-0.37 to -0.1)	0.95 (0.75 to 1.15)
Qatar	26 (12 to 48)	17.19 (8.01 to 31.72)	125 (56 to 242)	24.09 (10.76 to 46.65)	250.91 (116.14 to 462.56)	3.75 (2.74 to 4.96)	1.26 (1.16 to 1.37)
Republic of Korea	881 (395 to 1619)	5.51 (2.47 to 10.12)	1175 (505 to 2335)	12.33 (5.29 to 24.5)	197.41 (90.66 to 358.21)	0.33 (-0.01 to 0.78)	3.04 (2.56 to 3.53)
Republic of Moldova	56 (23 to 111)	3.55 (1.47 to 7.05)	47 (20 to 94)	6.05 (2.53 to 12.16)	56.52 (24.42 to 109.15)	-0.16 (-0.33 to 0.05)	2.04 (1.6 to 2.49)
Romania	393 (168 to 800)	5.24 (2.24 to 10.65)	463 (197 to 909)	11.42 (4.86 to 22.41)	126.84 (56.36 to 242.02)	0.18 (-0.1 to 0.7)	3.1 (2.74 to 3.47)
Russian Federation	3454 (1498 to 6617)	7.63 (3.31 to 14.62)	3262 (1341 to 6474)	9.49 (3.9 to 18.83)	109.44 (48.26 to 207.63)	-0.06 (-0.19 to 0.1)	0.22 (-0.13 to 0.57)
Rwanda	1777 (770 to 3306)	43.08 (18.67 to 80.13)	1754 (796 to 3203)	28.07 (12.73 to 51.24)	88.38 (40.39 to 164.2)	-0.01 (-0.38 to 0.55)	-1.45 (-1.81 to -1.1)

Saint Kitts and Nevis	4 (2 to 7)	20.67 (9.1 to 39.65)	5 (2 to 9)	28.64 (12.64 to 56.75)	214.62 (96.4 to 403.02)	0.23 (0.01 to 0.49)	0.77 (0.62 to 0.92)
Saint Lucia	12 (6 to 22)	18.1 (8.2 to 33.16)	11 (5 to 21)	24.95 (11.16 to 46.73)	193.81 (91.1 to 362.91)	-0.08 (-0.26 to 0.15)	0.44 (0.05 to 0.83)
Saint Vincent and the Grenadines	7 (3 to 14)	13.28 (5.93 to 25.11)	9 (4 to 18)	27.43 (12.12 to 52.47)	197.3 (89.42 to 374.97)	0.3 (0.07 to 0.59)	2.53 (2.41 to 2.66)
Samoa	11 (5 to 19)	13.03 (6.23 to 22.52)	12 (5 to 23)	12.3 (5.64 to 23.26)	107.57 (49.44 to 194.97)	0.07 (-0.22 to 0.41)	-0.14 (-0.35 to 0.07)
San Marino	1 (0 to 2)	15.8 (7.06 to 30.59)	1 (1 to 3)	19.45 (8.55 to 37.54)	272.31 (128.89 to 493.3)	0.41 (0.19 to 0.68)	0.83 (0.74 to 0.93)
Sao Tome and Principe	8 (4 to 14)	11.29 (5.21 to 19.89)	9 (4 to 17)	9.54 (4.45 to 17.51)	49.77 (22.96 to 89.63)	0.17 (-0.2 to 0.67)	-1.3 (-1.57 to -1.04)
Saudi Arabia	645 (299 to 1148)	7.73 (3.59 to 13.76)	1766 (798 to 3340)	18.26 (8.25 to 34.53)	211.05 (100.23 to 380.73)	1.74 (0.93 to 2.68)	2.91 (2.81 to 3)
Senegal	257 (116 to 474)	5.79 (2.62 to 10.68)	405 (185 to 756)	5.21 (2.38 to 9.72)	14.38 (6.62 to 26.84)	0.58 (-0.01 to 1.43)	-0.71 (-0.94 to -0.49)
Serbia	172 (77 to 329)	6.12 (2.73 to 11.71)	146 (63 to 292)	7.36 (3.15 to 14.64)	112.32 (51.65 to 210.49)	-0.15 (-0.31 to 0.04)	0.7 (0.59 to 0.8)
Seychelles	1 (1 to 2)	4.02 (1.83 to 7.43)	2 (1 to 4)	7.33 (3.27 to 14.12)	62.94 (28.95 to 119.14)	0.67 (0.31 to 1.1)	1.89 (1.7 to 2.08)
Sierra Leone	166 (69 to 321)	8.57 (3.56 to 16.54)	444 (196 to 842)	10.41 (4.59 to 19.72)	14.56 (6.53 to 26.92)	1.67 (0.53 to 3.6)	0.44 (0.2 to 0.67)
Singapore	43 (20 to 80)	4.72 (2.14 to 8.66)	97 (41 to 191)	9.4 (3.97 to 18.4)	171.95 (78.57 to 314.35)	1.24 (0.64 to 2.04)	2.36 (2.23 to 2.49)
Slovakia	70 (30 to 137)	3.99 (1.71 to 7.79)	75 (32 to 149)	6.71 (2.84 to 13.35)	87.78 (38.9 to 168)	0.07 (-0.14 to 0.31)	1.72 (1.67 to 1.77)
Slovenia	46 (20 to 91)	8.35 (3.57 to 16.32)	54 (22 to 104)	13.35 (5.54 to 25.86)	228.59 (101.9 to 415.78)	0.16 (-0.03 to 0.4)	1.78 (1.64 to 1.92)

Solomon Islands	8 (3 to 13)	3.85 (1.77 to 6.82)	22 (10 to 42)	6.84 (3.15 to 12.75)	28.78 (13.24 to 52.75)	1.95 (1.16 to 2.98)	1.85 (1.55 to 2.15)
Somalia	591 (217 to 1197)	13.88 (5.09 to 28.11)	768 (333 to 1473)	6.51 (2.82 to 12.49)	8.82 (3.92 to 16.95)	0.3 (-0.25 to 1.34)	-2.35 (-2.57 to -2.13)
South Africa	3262 (1490 to 5618)	18.89 (8.63 to 32.53)	3803 (1657 to 7157)	19.42 (8.46 to 36.54)	71.44 (31.18 to 135.47)	0.17 (-0.18 to 0.61)	-0.66 (-1.65 to 0.35)
South Sudan	1305 (515 to 2560)	39.85 (15.72 to 78.14)	1310 (616 to 2414)	24.74 (11.63 to 45.58)	37.06 (17.59 to 65.47)	0 (-0.41 to 0.67)	-1.6 (-1.95 to -1.24)
Spain	912 (414 to 1729)	8.18 (3.71 to 15.52)	1110 (496 to 2119)	12.28 (5.49 to 23.44)	183.05 (87.07 to 328.91)	0.22 (-0.05 to 0.6)	1.57 (1.26 to 1.88)
Sri Lanka	444 (218 to 743)	6.07 (2.98 to 10.16)	526 (239 to 967)	7.7 (3.51 to 14.17)	82 (38.75 to 149.89)	0.18 (-0.16 to 0.63)	0.96 (0.73 to 1.2)
Sudan	1013 (378 to 2057)	9.02 (3.37 to 18.32)	2160 (991 to 3912)	10.66 (4.89 to 19.32)	34.83 (15.7 to 64.2)	1.13 (0.21 to 3.21)	0.59 (0.23 to 0.94)
Suriname	27 (12 to 50)	15.64 (7.01 to 29.36)	43 (19 to 82)	22.09 (9.83 to 42.49)	114.05 (50.8 to 217.64)	0.6 (0.29 to 1)	1.13 (0.91 to 1.35)
Sweden	252 (109 to 488)	11.94 (5.19 to 23.14)	443 (194 to 896)	18.71 (8.18 to 37.79)	292.95 (138.15 to 538.94)	0.76 (0.47 to 1.14)	1.68 (1.4 to 1.96)
Switzerland	227 (104 to 431)	14.41 (6.57 to 27.37)	301 (134 to 580)	17.28 (7.71 to 33.32)	244.55 (116.48 to 438.98)	0.33 (0.1 to 0.62)	0.57 (0.52 to 0.62)
Syrian Arab Republic	1050 (513 to 1807)	13.97 (6.82 to 24.02)	1078 (526 to 1882)	18.31 (8.94 to 31.97)	129.77 (65.21 to 218.87)	0.03 (-0.24 to 0.38)	1.91 (1.34 to 2.49)
Taiwan (Province of China)	515 (225 to 997)	7.03 (3.07 to 13.63)	603 (255 to 1184)	14.11 (5.98 to 27.73)	212.82 (95.01 to 399.39)	0.17 (-0.11 to 0.61)	1.24 (0.75 to 1.73)
Tajikistan	61 (24 to 123)	2.13 (0.83 to 4.3)	142 (56 to 288)	3.45 (1.37 to 6.99)	14.33 (5.72 to 29.44)	1.33 (0.87 to 2)	1.63 (1.08 to 2.17)
Thailand	1164 (552 to 2063)	5.09 (2.41 to 9.01)	1626 (745 to 3018)	10.87 (4.98 to 20.18)	107.86 (52.08 to 189.03)	0.4 (0.05 to 0.83)	2.04 (1.7 to 2.39)

Timor-Leste	55 (16 to 126)	13.57 (3.86 to 30.91)	49 (21 to 87)	7.32 (3.15 to 13.05)	29.87 (12.93 to 52.87)	-0.12 (-0.6 to 1.62)	-1.81 (-2.29 to -1.34)
Togo	98 (46 to 173)	4.54 (2.14 to 7.98)	289 (131 to 549)	7.3 (3.3 to 13.88)	16.15 (7.41 to 30.53)	1.94 (0.99 to 3.04)	1.01 (0.67 to 1.36)
Tokelau	0 (0 to 0)	10.08 (4.63 to 18.54)	0 (0 to 0)	15.94 (7 to 29.81)	154.79 (70.58 to 281.18)	0.08 (-0.28 to 0.5)	1.79 (1.59 to 1.99)
Tonga	8 (4 to 15)	16.13 (7.31 to 28.97)	10 (4 to 18)	21.25 (9.68 to 39.74)	170.96 (81.07 to 312.29)	0.19 (-0.02 to 0.43)	0.57 (0.23 to 0.9)
Trinidad and Tobago	143 (70 to 251)	27.65 (13.52 to 48.51)	153 (71 to 283)	42.28 (19.69 to 78.23)	324.15 (153.48 to 582.55)	0.07 (-0.17 to 0.35)	1.38 (1.2 to 1.57)
Tunisia	344 (161 to 610)	8.45 (3.96 to 14.98)	494 (213 to 958)	14.3 (6.18 to 27.76)	129.35 (59.04 to 241.18)	0.43 (0.06 to 0.88)	2 (1.89 to 2.12)
Turkey	3511 (1610 to 6118)	12.53 (5.75 to 21.84)	3616 (1587 to 6723)	16.22 (7.12 to 30.16)	129.69 (61.16 to 235.01)	0.03 (-0.25 to 0.38)	0.31 (-0.08 to 0.69)
Turkmenistan	185 (86 to 328)	9.8 (4.57 to 17.39)	201 (86 to 395)	10.47 (4.48 to 20.6)	52.11 (22.53 to 103.93)	0.08 (-0.27 to 0.52)	0.26 (-0.18 to 0.7)
Tuvalu	1 (0 to 1)	15.65 (6.94 to 29.97)	1 (0 to 2)	19.91 (9.25 to 36.21)	149.77 (70.31 to 271.61)	0.42 (-0.17 to 1.44)	0.79 (0.43 to 1.16)
Uganda	2324 (1006 to 4274)	22.53 (9.75 to 41.43)	4090 (1923 to 7563)	17.1 (8.04 to 31.62)	38.8 (17.92 to 71.8)	0.76 (0.11 to 1.77)	-0.85 (-1.19 to -0.51)
Ukraine	1121 (451 to 2210)	7.46 (3 to 14.71)	696 (286 to 1431)	7.77 (3.2 to 15.97)	75.66 (31.86 to 147.05)	-0.38 (-0.49 to -0.23)	-0.07 (-0.49 to 0.35)
United Arab Emirates	178 (85 to 311)	25.07 (12.03 to 43.8)	479 (220 to 871)	31.59 (14.53 to 57.44)	388.33 (183.53 to 679.34)	1.69 (1.18 to 2.3)	1.78 (1.04 to 2.52)
United Kingdom	3720 (1692 to 6936)	24.98 (11.36 to 46.58)	3969 (1803 to 7606)	25.54 (11.6 to 48.95)	336.38 (164.54 to 588.94)	0.07 (-0.03 to 0.17)	-0.28 (-0.52 to -0.03)
United Republic of Tanzania	2971 (1349 to 5202)	19.83 (9 to 34.72)	6346 (2923 to 11463)	20.67 (9.52 to 37.34)	47.26 (21.71 to 85.19)	1.14 (0.46 to 1.99)	0.42 (0.05 to 0.79)

United States of America	21569 (9394 to 41630)	29.24 (12.73 to 56.44)	40829 (18700 to 73685)	49.92 (22.86 to 90.09)	520.25 (261.15 to 867.16)	0.89 (0.57 to 1.34)	2.42 (2.19 to 2.64)
United States Virgin Islands	10 (5 to 19)	24.23 (10.99 to 45.81)	12 (5 to 23)	44.75 (20.08 to 86.82)	478.66 (224.39 to 890.45)	0.18 (-0.02 to 0.41)	2.13 (2.03 to 2.24)
Uruguay	83 (36 to 159)	7.73 (3.32 to 14.77)	141 (62 to 265)	14.85 (6.52 to 27.8)	149.18 (68.3 to 272.29)	0.7 (0.34 to 1.15)	2.13 (1.91 to 2.34)
Uzbekistan	713 (310 to 1354)	6.68 (2.9 to 12.67)	1268 (521 to 2530)	9.65 (3.97 to 19.24)	53.78 (22.78 to 105.75)	0.78 (0.42 to 1.21)	1.03 (0.61 to 1.45)
Vanuatu	6 (3 to 12)	7.8 (3.64 to 14.06)	16 (8 to 29)	11.66 (5.41 to 20.67)	57.68 (26.64 to 101.66)	1.53 (0.82 to 2.42)	1.17 (0.97 to 1.38)
Venezuela (Bolivarian Republic of)	1928 (911 to 3422)	21.21 (10.02 to 37.64)	1737 (762 to 3390)	18.68 (8.2 to 36.45)	123.98 (55.17 to 238.77)	-0.1 (-0.33 to 0.18)	-0.86 (-1.31 to -0.42)
Viet Nam	460 (208 to 856)	1.38 (0.62 to 2.57)	1259 (555 to 2414)	4.48 (1.98 to 8.6)	38.8 (17.18 to 70.97)	1.74 (0.92 to 2.75)	4.65 (4.27 to 5.02)
Yemen	658 (258 to 1302)	7.74 (3.03 to 15.32)	716 (330 to 1329)	4.39 (2.02 to 8.15)	12.17 (5.64 to 22.16)	0.09 (-0.36 to 0.89)	-1.13 (-1.9 to -0.36)
Zambia	1405 (500 to 2734)	29.83 (10.62 to 58.07)	1765 (840 to 3111)	18.03 (8.58 to 31.79)	44.65 (20.83 to 79.71)	0.26 (-0.31 to 1.64)	-1.68 (-1.97 to -1.39)
Zimbabwe	239 (111 to 435)	3.96 (1.84 to 7.22)	334 (154 to 589)	4.39 (2.02 to 7.73)	11.21 (5.13 to 19.8)	0.4 (0.04 to 0.88)	0.58 (0.29 to 0.88)

DALYs, disability-adjusted life-years;
BMI, body mass index;
PAF, population attribuTable fraction;
EAPC, estimated annual percentage change;
95% UI, 95% uncertainty interval;
95% CI, certain interval.
